# Multifactorial impacts of blood culture process optimization on clinical outcomes and healthcare economics in bloodstream infection management

**DOI:** 10.3389/fcimb.2026.1699905

**Published:** 2026-03-13

**Authors:** Lipeng Wang, Fengzhen Yang, Chunling Zhou, Xiaochen Yang, Jincheng Rong, Xiaohui Chi, Rui Guo, Na Li, Li Sheng, Lihua Jiang, Qi Zhao, Maoli Yi

**Affiliations:** 1 Department of Laboratory Medicine, Yantai Yuhuangding Hospital, Yantai, Shandong, China; 2 The Second Clinical Medical College, Binzhou Medical University, Yantai, Shandong, China; 3 Department of Blood Transfusion, Yantai Affiliated Hospital of Binzhou Medical University, Yantai, Shandong, China

**Keywords:** blood culture, bloodstream infections, clinical outcome, healthcare economics, workflow optimization

## Abstract

**Objective:**

To investigate the clinical and health economic impacts of individual workflow components. The effects of phased process optimizations were quantified using empirical data.

**Methods:**

From August 2023 to October 2024, the outcomes before and after workflow optimization were compared. The control group comprised patients processed under conventional protocols, while the experimental group included those managed with optimized workflows.

**Results:**

Phase I revealed significant temporal reductions through MALDI-TOF enhanced biofilm identification. The Gram-stain reporting time decreased from 33.3 h to 25.7 h (*P* < 0.05), species identification from 72.5 h to 42.6 h (*P* < 0.05). Phase II implementation of 24/7 processing via BacT/Alert Virtuo achieved marked improvements across all metrics, namely Gram-stain (37.4 h vs, 17.9 h), identification (42.6 h vs. 36.4 h) and AST reporting (68.4 h vs. 63.4 h) (all *P* < 0.05). Phase III optimization through Advanced Expert System (AES)-enabled automated preliminary AST reporting during off-hours significantly accelerated therapeutic guidance (63.4 h vs. 47.2 h, *P* < 0.05).

**Conclusion:**

Quantitative analysis of these optimizations elucidated their differential impacts on blood culture workflows and prognostic determinants. These findings advocate for laboratory innovation as a driver of antimicrobial stewardship and precision medicine for management of microbial bloodstream infections.

## 1 Introduction

Microbial bloodstream infections (BSIs) are one of the most severe conditions and are a leading cause of mortality worldwide (Fabre et al., 2022; Destache et al., 2023). It is estimated that BSI affects approximately 30 million individuals annually, resulting in 6 million deaths (Dunbar et al., 2022). The presence of microorganisms in the bloodstream—whether persistent, intermittent or transient – poses a threat to every organ system (Tille, 2021), leading to severe complications such as septic shock, multiorgan failure and even death (Han et al., 2024). The annual incidence of BSI is 150 cases per 100,000 population, with an all-cause crude mortality rate of 17% within 30 days of positive blood culture (BC) (Holmes et al., 2024). Mortality associated with BSIs ranges between 30% and 50%, and are among the costliest conditions to treat in hospitals. Studies have indicated that BSI imposes an annual economic burden of up to US$25 billion in the United States alone (Kim et al., 2024).

As BSI typically constitute life-threatening infections, timely isolation and detection of the pathogenic microorganisms in the blood circulation is paramount. Rapid and accurate pathogen identification combined with antimicrobial susceptibility testing (AST) form the cornerstone for initiating appropriate targeted antimicrobial therapy, which is critical for improving patient outcomes (Nomura et al., 2020; Ai et al., 2024; Zeng et al., 2025). Although BC remains the gold standard for BSI diagnosis, it has inherent limitations, particularly regarding extended turnaround times (TATs) (Peri et al., 2024). The complete BC process encompasses a complex diagnostic cascade involving infection confirmation, microbial species identification and evaluation of antimicrobial susceptibility profiles (Kim et al., 2024). This comprehensive workflow typically requires a minimum 2–3 day TAT, necessitating empirical broad-spectrum antibiotic therapy initiation by clinicians. Such practice inevitably introduces treatment inaccuracy and deviations from personalized therapeutic approaches. Therefore, optimizing BC processes is crucial for ensuring accurate detection of true bacteremia (particularly in septic patients) while mitigating risks associated with unnecessary BC procedures, including inappropriate antibiotic treatment of false-positive results, delayed discharge and increased healthcare expenditures.

Current research remains limited on how BC process optimization delivers clinical and economic benefits for hospitalized patients. Over a one-year period, our microbiology laboratory implemented iterative enhancements to BC workflows through the introduction of novel equipment and advanced functionalities in information systems. In the present study, to investigate the clinical and health economic impacts of individual workflow components, the effects of phased process optimizations were quantified using empirical data. This approach elucidated the roles of specific optimization factors within the BC workflow process and their downstream effects on clinical outcomes and healthcare resource utilization.

## 2 Methods

### 2.1 Study design

This study was conducted at Yantai Yuhuangding Hospital Affiliated to Qingdao University, a 4,000-bed tertiary Grade-A general hospital. From August 2023 to October 2024, the clinical microbiology workflow for BSI management was optimized.

Patients meeting the following three conditions during each phase of the study were enrolled: (1) hospitalized adults (age ≥ 18 years) with suspected BSI and positive BC results; (2) pathogens were isolated from their BCs; and (3) availability of complete clinical and laboratory data. Exclusion criteria comprised: (1) patients discharged prior to microbiological reporting; and (2) cases with BC results indicating contaminating organisms.

Employing a retrospective pre-post controlled design, outcomes were compared before and after workflow optimization. The control group comprised patients processed under conventional protocols, while the experimental group included those managed with optimized workflows. Data were extracted from the Laboratory Information System (LIS), Hospital Information System (HIS), and health-economic databases.

The study protocol was approved by Yantai Yuhuangding Hospital Ethics Committee, and conducted in accordance with the Declaration of Helsinki and Declaration of Istanbul. Informed consent was waived as no personally identifiable information was used in the analyses.

### 2.2 Phase I workflow optimization

#### 2.2.1 Pre-optimization (June 1, 2023 – August 31, 2023)

BC specimen collection, transportation, receipt and loading were performed according to CLSI guidelines. Between 7:00 am and 5:00 pm, collected BC bottles (BacT/Alert FAN PLUS bottles with adsorbent polymeric beads) were transported to the microbiology laboratory and incubated in the BacT/Alert 3D microbial detection system (bioMérieux, France) for continuous monitoring. Upon positive detection by BacT/Alert 3D, a sterile syringe was immediately used to aspirate the positive culture for smear preparation and Gram-staining, with results reported to clinicians. Concurrently, 0.1 mL of the sample was inoculated onto blood agar plates, chocolate agar plates and MacConkey agar (MAC) plates, followed by overnight incubation in a 35 °C incubator with 5% CO_2_. Bacterial identification was performed using matrix-assisted laser desorption/ionization time-of-flight mass spectrometry (MALDI-TOF/MS) the following day and AST was conducted using Vitek 2 XL (bioMérieux, France). On the third day, bacterial identification and AST results were reported to clinicians via the LIS.

#### 2.2.2 Post-optimization (October 1, 2023 – December 31, 2023)

**Figure 1:**
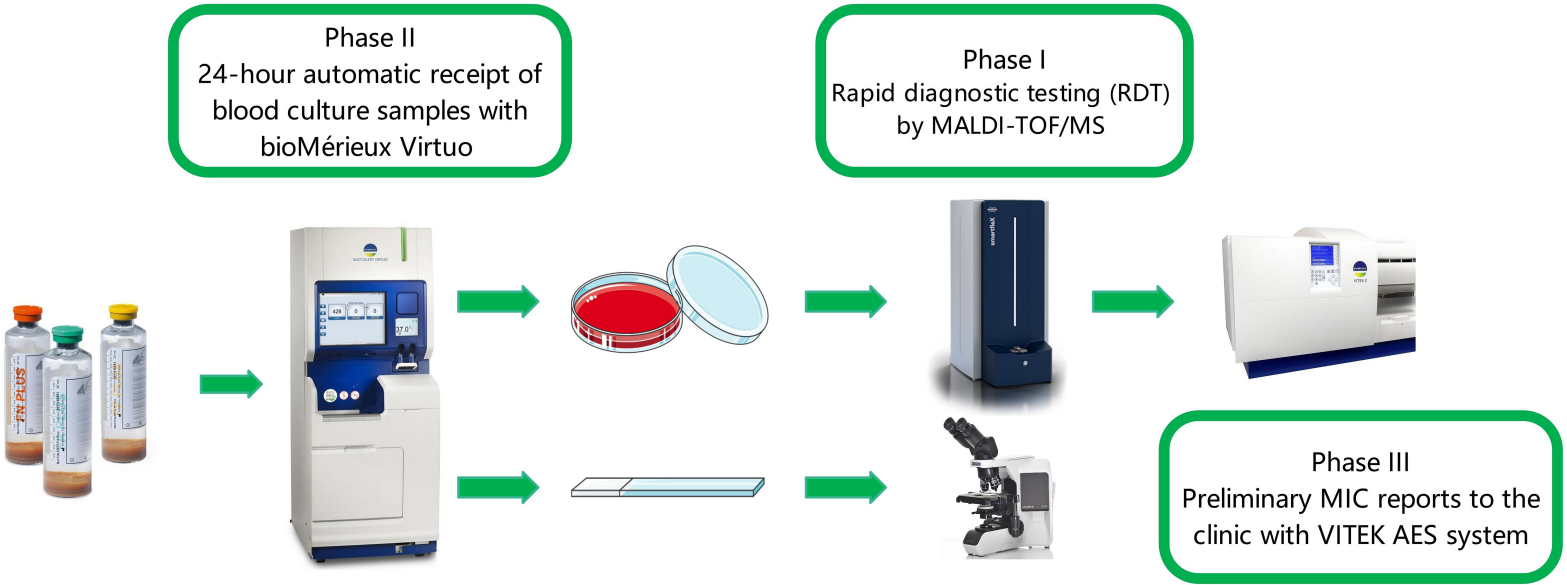
Overview of the study design and workflow (optimization measures for the blood culture process across three phases).

BC collection and processing remained unchanged. Starting at 7:00 am the next day, positive BCs were immediately subjected to smear preparation and Gram-staining, with results promptly communicated to clinicians. Subcultured plates from positive bottles were incubated for 8 h in a 35 °C incubator with 5% CO_2_, followed by MALDI-TOF/MS identification using visible bacterial colonies. Preliminary identification results were reported to clinicians and AST was performed using Vitek 2 XL (bioMérieux, France). Final identification and AST results were delivered via LIS on the third day **Figure 1**.

### 2.3 Phase II of process optimization (focused on nighttime specimens)

#### 2.3.1 Pre-optimization (January 1, 2024 – May 9, 2024)

BC collection and processing mirrored phase I post-optimization protocols.

#### 2.3.2 Post-optimization (May 10, 2024 – July 31, 2024)

The BacT/Alert 3D system was replaced with the BacT/Alert Virtuo system (bioMérieux, France). Training programs were implemented for clinicians, nurses and specimen transporters to enable 24-h BC bottle loading. Subsequent procedures remained consistent with previous phases (**Figure 1**).

### 2.4 Phase III of process optimization

#### 2.4.1 Pre-optimization (May 10, 2024 – July 31, 2024)

BC protocols aligned with phase II post-optimization.

#### 2.4.2 Post-optimization (August 1, 2024 – October 15, 2024)

24-h BC loading was maintained. Starting at 7:00 am the following day, positive cultures underwent immediate Gram-staining and reporting. Subcultured plates were incubated for 8 h at 35 °C with 5% CO_2_, followed by MALDI-TOF/MS identification using macroscopic bacterial colonies. Preliminary identification results were communicated to clinicians, while AST was performed using Vitek 2 XL (bioMérieux, France). Leveraging the integrated Advanced Expert System (AES) and Advanced Reporting Tools (ART), along with a blocking reagent kit to validate susceptibility results prone to Very Major Errors (VME) and Major Errors (ME) identified through laboratory data analysis, an automated LIS-based reporting mechanism was implemented. This enabled real-time nocturnal transmission of AST results to clinicians on the same day (**Figure 1**).

### 2.5 Study endpoints

#### 2.5.1 Laboratory endpoints

BC specimen collection time/BC loading time onto detection systems/time to positivity (TTP) of BCs/TAT for Gram-staining reports of positive cultures/time to microbial species identification for positive cultures/preliminary AST report time/final AST report time/total TAT from specimen collection to clinician receipt of reports.

#### 2.5.2 Clinical outcome endpoints

Total duration of antimicrobial therapy/length of hospital stay (LOS)/patient prognosis and clinical outcomes (e.g., survival, complications).

#### 2.5.3 Health economic endpoints

Antimicrobial drug costs/total hospitalization costs/laboratory testing expenses.

### 2.6 Statistical analysis

All continuous variables underwent normality testing (Shapiro-Wilk test) and homogeneity of variance analysis (Levene’s test). Normally distributed data are presented as the mean ± standard deviation and compared using Student’s *t*-test. Non-normally distributed data are expressed as median (interquartile range) and were compared using the Mann-Whitney U test. Categorical variables were analyzed using the χ² test or Fisher’s exact test, when appropriate. Statistical analyses were carried out using SPSS 26.0 (IBM Corp., USA), with a two-tailed *P* < 0.05 considered to be a statistically significant finding.

## 3 Results

This study was structured into three sequential phases, each comprising pre-optimization and post-optimization cohorts. Phase I included 83 and 90 patients in the pre- and post-optimization groups respectively, phase II included 83 and 78 and phase III included 78 and 75, respectively. As detailed in **Table 1**, the baseline characteristics analysis revealed no statistically significant differences in mean age or gender distribution between comparative groups across all phases. A comparative analysis of white blood cell/neutrophil counts, C-reactive protein, and procalcitonin levels between pre-optimization and post-optimization groups was performed across all three phases, and revealed no significant differences. The composition of primary infection foci in BSI is also presented in **Table 1**. Pathogen distribution analysis of BC isolates in the pre- and post-optimization groups across the three phases is detailed in **Table 2**. Notably, no statistically significant differences were detected in those inter-group comparisons.

**Table 1:** Baseline demographics and clinical characteristics of subjects.

	Phase I			Phase II			Phase III		
	Pre-op (n=83)	Post-op (n=90)	*p*	Pre-op (n=83)	Post-op (n=78)	*p*	Pre-op (n=78)	Post-op (n=75)	*p*
Age (years)									
median [IQR]	69.0 [60.0, 77.5]	68.0 [57.25, 75.0]	0.394	67.0 [57.5, 74.5]	63.5 [55.0, 74.5]	0.263	63.5 [55.0, 74.5]	62.0 [55.0, 71.0]	0.614
Gender, n(%)									
Male	55 (66.27)	58 (64.44)	0.802	50 (60.24)	47 (60.26)	0.998	47 (60.26)	45 (60.00)	0.974
Female	28 (33.73)	32 (35.56)		33 (39.76)	31 (39.74)		31 (39.74)	30 (40.00)	
Infection source, n(%)									
Digestive	64 (77.11)	60 (66.67)	0.128	36 (43.37)	28 (35.90)	0.333	28 (35.90)	25 (33.33)	0.739
Respiratory	11 (13.25)	11 (12.22)	0.839	16 (19.28)	20 (25.64)	0.333	20 (25.64)	16 (21.33)	0.530
Urinary	0	3 (3.33)	0.247*	4 (4.82)	7 (8.97)	0.296	7 (8.97)	10 (13.33)	0.391
Hematologic	0	5 (5.56)	0.060*	10 (12.05)	8 (10.26)	0.718	8 (10.26)	11 (14.67)	0.408
Nervous	0	0	NA	3 (3.61)	0	0.246*	0	0	NA
Surgical related	7 (8.43)	8 (8.89)	0.897	2 (2.41)	6 (7.69)	0.158*	6 (7.69)	4 (5.33)	0.793
Others	1 (1.20)	3 (3.33)	0.622*	12 (14.46)	9 (11.54)	0.583	9 (11.54)	9 (12.00)	0.929
Infection related testing, median [IQR]									
WBC, 10^9^/L	11.09 [6.89, 15.55]	9.95 [2.55, 14.48]	0.164	9.54 [4.55, 14.16]	11.15 [6.29, 15.55]	0.149	11.15 [6.29, 15.55]	8.91 [5.41, 12.37]	0.106
Neutrophil, 10^9^/L	10.10 [5.81, 14.24]	8.85 [4.53, 13.34]	0.103	8.57 [3.76, 13.24]	9.80 [4.90, 13.86]	0.129	9.80 [4.90, 13.86]	7.62 [4.45, 11.71]	0.094
CRP, mg/L	81.93 [24.09, 202.50]	63.35 [18.82, 161.10]	0.330	73.70 [20.37, 154.09]	114.00 [37.46, 188.42]	0.158	114.00 [37.46, 188.42]	89.63 [36.74, 204.52]	0.864
PCT, ng/mL	2.04 [0.37, 16.90]	1.45 [0.29, 5.61]	0.146	1.03 [0.22, 10.36]	0.79 [0.26, 13.66]	0.983	0.79 [0.26, 13.66]	0.92 [0.25, 4.68]	0.930

pre-op, pre-optimization; post-op, post-optimization; NA, not applicable; WBC, white blood cell; CRP, C-reactive protein; PCT, procalcitonin.

*Fisher’s exact test.

**Table 2:** Composition ratio of strains identified in BCs before and after optimization.

Organism	Phase I			Phase II			Phase III		
	Pre-op (n=83)	Post-op (n=90)	*p*	Pre-op (n=84)	Post-op (n=81)	*p*	Pre-op (n=81)	Post-op (n=76)	*p*
Gram-positive organisms, n(%)									
*Staphylococcus aureus*	4 (4.82)	4 (4.44)	1.000*	6 (7.14)	6 (7.41)	0.948	6 (7.41)	4 (5.26)	0.824
*MRSA*	1 (1.20)	2 (2.22)	1.000*	0	3 (3.70)	0.116*	3 (3.70)	0	0.246*
*Coagulase-negative Staphylococcus*	19 (22.89)	12 (13.33)	0.101	10 (11.90)	12 (14.81)	0.583	12 (14.81)	12 (15.79)	0.865
*Enterococcus* spp.	3 (3.61)	10 (11.11)	0.062	8 (9.52)	8 (9.88)	0.939	8 (9.88)	4 (5.26)	0.277
*Streptococcus* spp.	4 (4.82)	5 (5.56)	1.000*	9 (10.71)	6 (7.41)	0.460	6 (7.41)	0	0.029*
*Listeria monocytogenes*	0	1 (1.11)	1.000*	0	0	NA	0	0	NA
Gram-negative organisms, n(%)									
*Acinetobacter* spp.	4 (4.82)	2 (2.22)	0.429*	2 (2.38)	0	0.497*	0	3 (3.95)	0.111*
*Escherichia coli*	22 (26.51)	26 (28.89)	0.727	19 (22.62)	25 (30.86)	0.231	25 (30.86)	28 (36.84)	0.429
*Klebsiella* spp.	13 (15.66)	15 (16.67)	0.858	18 (21.43)	15 (18.52)	0.640	15 (18.52)	20 (26.32)	0.241
*Pseudomonas aeruginosa*	2 (2.41)	7 (7.78)	0.171*	2 (2.38)	0	0.497*	0	2 (2.63)	0.233*
*Enterobacter* spp.	1 (1.20)	1 (1.11)	1.000*	5 (5.95)	6 (7.41)	0.708	6 (7.41)	3 (3.95)	0.497*
*Aeromonas* spp.	3 (3.61)	3 (3.33)	1.000*	0	0	NA	0	0	NA
*Stenotrophomonas maltophilia*	0	0	NA	0	1 (1.23)	0.491*	1 (1.23)	0	1.000*
Others	5 (6.02)	3 (3.33)	0.483*	3 (3.57)	0	0.246*	0	0	NA
*CRE*	0	0	NA	0	2 (2.47)	0.239*	2 (2.47)	2 (2.63)	1.000*
*CRO*	2 (2.41)	2 (2.22)	1.000*	1 (1.19)	2 (2.47)	0.616*	2 (2.47)	3 (3.95)	0.674*
*Candida* spp., n(%)	3 (3.61)	1 (1.11)	0.351*	2 (2.38)	2 (2.47)	1.000*	2 (2.47)	0	0.497*

NA, not applicable; pre-op, pre-optimization; post-op, post-optimization; MRSA, methicillin-resistant Staphylococcus aureus; CRE, carbapenem-resistant Enterobacterales; CRO, carbapenemase-producing Organism​.

*Fisher’s exact test.

**Figure 2:**
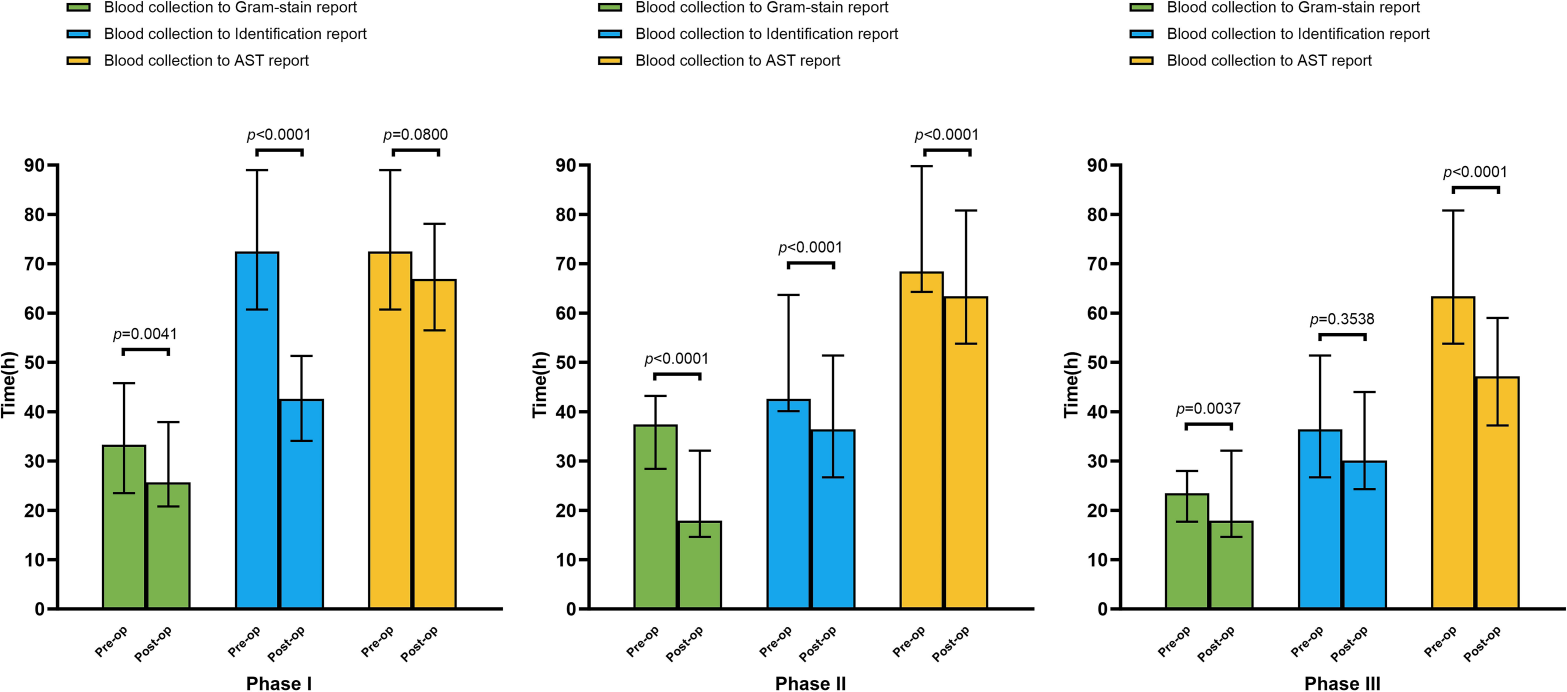
Results demonstrating reporting timelines (blood collection to gram-stain reporting, species identification reporting, and antimicrobial susceptibility testing reporting) before and after optimization across the three phases.

Using BC collection as the unified temporal reference, we analyzed time intervals from specimen acquisition to critical reporting milestones across optimization phases (**Figure 2**). Phase I demonstrated significant temporal reductions through MALDI-TOF-enhanced biofilm identification: Gram stain reporting time decreased from 33.3 h to 25.7 h (*P* < 0.05), species identification from 72.5 h to 42.6 h (*P* < 0.05). Though statistically non-significant, AST reporting showed an apparent downward trend (72.5 h vs. 66.9 h). Phase II implementation of 24/7 processing via BacT/Alert Virtuo achieved marked improvements across all metrics, namely Gram-staining (37.4 h vs. 17.9 h), identification (42.6 h vs. 36.4 h) and AST reporting (68.4 h vs. 63.4 h) (all *P* < 0.05). Phase III optimization through AES-enabled automated preliminary AST reporting during off-hours significantly accelerated therapeutic guidance (63.4 h vs. 47.2 h, *P* < 0.05), enabling earlier antimicrobial regimen adjustments.

**Figure 3:**
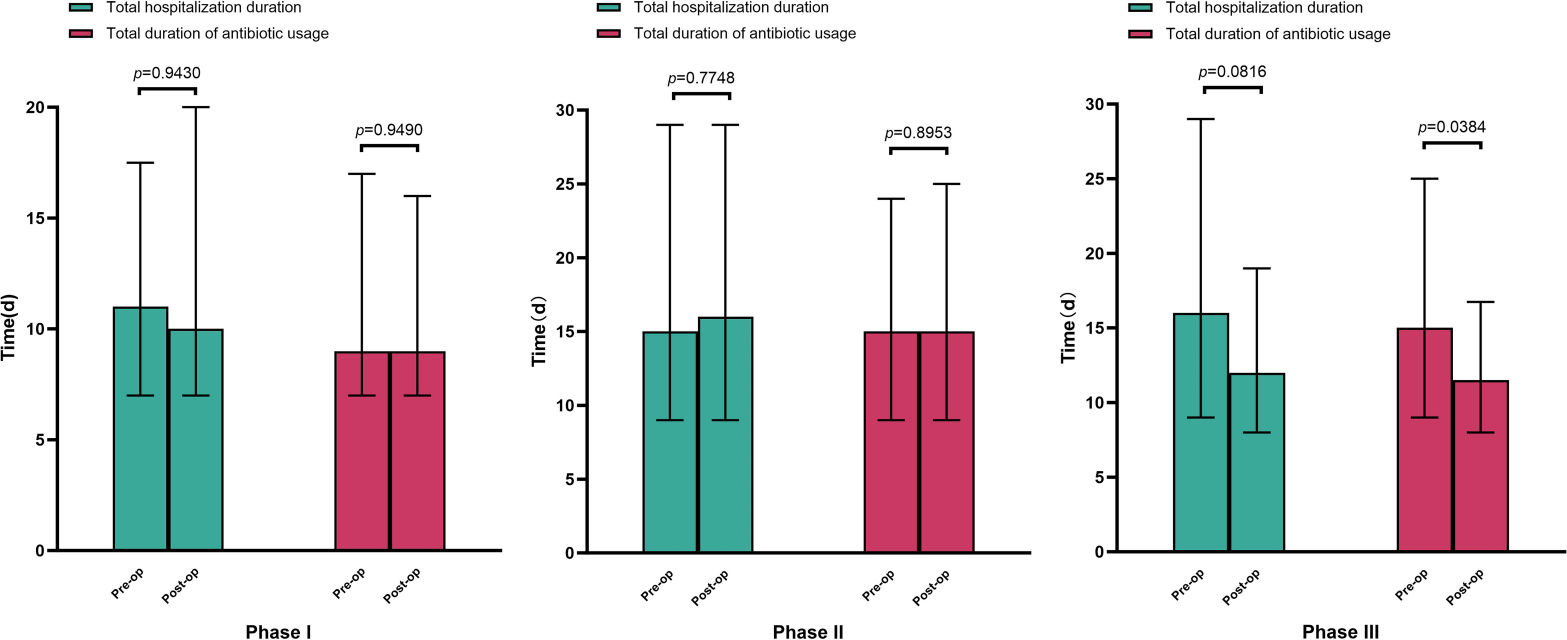
Total length of hospital stay and duration of antimicrobial use before and after optimization of the blood culture process across the three phases.

Clinical outcome analysis revealed differential phase impacts (**Figure 3**). Phases 1 and 2 showed no statistically significant reductions in LOS or antimicrobial therapy duration. However, phase III optimization achieved significant decreases in both therapeutic duration (median reduction, 2.8 days) and total LOS (median reduction, 3.5 days).

Survival outcomes demonstrated consistent clinical improvements across the phases (**Table 3**). Survival rates increased from 74.70% to 82.22% (phase I), 68.67% to 75.64% (phase II) and 75.64% to 86.67% (phase III), with corresponding mortality reductions of 25.30%→17.78%, 31.33%→24.36% and 24.36%→13.33%, though these differences did not reach statistical significance.

**Table 3:** Comparison of patient groups based on clinical outcomes and economic costs before and after optimization.

	Phase I			Phase II			Phase III		
	Pre-op (n=83)	Post-op (n=90)	*p*	Pre-op (n=83)	Post-op (n=78)	*p*	Pre-op (n=78)	Post-op (n=75)	*p*
Clinical outcomes, n(%)									
Recovery	62 (74.70)	74 (82.22)	0.228	57 (68.67)	59 (75.64)	0.325	59 (75.64)	65 (86.67)	0.082
Mortality	21 (25.30)	16 (17.78)		26 (31.33)	19 (24.36)		19 (24.36)	10 (13.33)	
Economic outcomes, median (IQR), CNY									
Total laboratory costs	5678.3 [4128.8, 10365.1]	4524.0 [3574.4, 9058.3]	0.069	6581.3 [4323.3, 16225.2]	8029.5 [5126.0, 14266.0]	0.387	8029.6 [5126.0, 14266.0]	4910.3 [3770.2, 8426.9]	< 0.001
Total antibacterial agents costs	2258.5 [1134.5, 5072.1]	1640.5 [611.9, 3819.5]	0.132	3828.5 [1076.4, 10037.4]	3127.3 [937.8, 9746.1]	0.516	3127.3 [937.8, 9746.1]	1181.1 [523.5, 6903.2]	0.025
Total hospitalization costs	34901.4 [25278.5, 74986.3]	30703.5 [19439.4, 48733.8]	0.035	44242.4 [18171.6, 145491.6]	41748.5 [26570.4, 91600.6]	0.949	41748.5 [26570.4, 91600.6]	28635.7 [17261.0, 62808.2]	0.003

pre-op, pre-optimization; post-op, post-optimization.

Health-economic evaluations revealed phase-specific financial impacts (**Table 3**). Phase I’s MALDI-TOF implementation reduced laboratory costs by 18.3% and total hospitalization expenses from ¥34,901.4 to ¥30,703.5 (*P* < 0.05). Phase II showed a neutral financial impact, with marginal laboratory cost increases. Phase III optimization through AES-driven automation achieved substantial cost reductions: laboratory expenses decreased by 38.8% (¥8,029.6→¥4,910.3); antimicrobial costs by 62.3% (¥3,127.3→¥1,181.1); and total hospitalization costs by 31.4% (¥41,748.5→¥28,635.7).

## 4 Discussion

BSI represent a critical clinical challenge due to their rapid progression and high mortality rates (Yin et al., 2022; Caspar et al., 2024; Dai et al., 2024; Kim et al., 2024). Epidemiological data indicate that BSI accounts for 40% of community-acquired and hospital-acquired sepsis/septic shock cases and affects 20% of intensive care unit patients (Timsit et al., 2020). Early diagnosis and precise antimicrobial therapy are pivotal to improving patient outcomes (Mahrous et al., 2020; Patel et al., 2021; Peri et al., 2022; Caspar et al., 2024). Studies have found that each hour of delay in BSI treatment correlates with increased mortality (Adamik et al., 2020; Peri et al., 2022) and that any postponement of antimicrobial therapy incrementally raises mortality risk between 12 and 72 h post-BC collection (Caspar et al., 2024). Consequently, clinical microbiology laboratories bear the responsibility to deliver faster, accurate and reliable BC identification and antimicrobial susceptibility reports.

In phase I of workflow optimization, we implemented MALDI-TOF mass spectrometry – a rapid diagnostic test – for preliminary pathogen identification and susceptibility profiling using bacterial colonies from 8-h subcultures of positive BCs. While direct identification methods from positive broth (e.g., Sepsityper^®^ kits and Vitek MS BC kits (Nomura et al., 2020)) exist, their high cost and suboptimal accuracy for Gram-positive bacteria remain limitations. Idelevich et al (Idelevich et al., 2014)found that short-term agar incubation (2, 4, 6, 8 or 12 h) yielded species-level identification rates for Gram-positive cocci of 1.2%, 18.6%, 64.0%, 96.5% and 98.8%, respectively and for Gram-negative bacilli 76.2%, 95.2%, 97.6%, 97.6% and 97.6%. Based on these findings, a protocol was adopted involving subculture to agar plates for short-term incubation followed by MALDI-TOF identification and AST – a balanced approach between costly/complex direct broth methods and time-consuming overnight cultures. The integration of MALDI-TOF reduced TATs from specimen collection to Gram-staining and species identification reports from 33.3/72.5 h to 25.7/42.6 h, aligning with the time reductions reported by Fumio Nomura (Nomura et al., 2020). From a health economic perspective, MALDI-TOF implementation reduced laboratory testing costs, antimicrobial expenditures and significantly lowered total hospitalization costs. Thus, MALDI-TOF has proven its efficacy in accelerating the optimization of antibiotic treatment of BSI.

In phase II, following the introduction of the BacT/Alert Virtuo BC system and comprehensive training for specimen transport personnel, automated loading of BC bottles was achieved during unmanned night shifts without extending laboratory working hours. As patients’ medical histories and potential pathogens are often unclear at the time of BC collection, immediate loading into the detection system is critical for rapid and accurate diagnosis (Adamik et al., 2020). Our results demonstrated significant reductions in time-to-Gram-stain reporting (37.4 h vs. 17.9 h) and species identification (42.6 h vs. 36.4 h, *P* < 0.05) post-implementation. This optimization reduced diagnostic delays, facilitated earlier de-escalation of broad-spectrum empirical therapy and mitigated unnecessary antimicrobial use – crucial advantages in combating antimicrobial resistance (Weinbren et al., 2018; Adamik et al., 2020; Daneman et al., 2025). However, the lack of 24/7 laboratory operations still caused inevitable loading delays. Previous studies have validated the superior detection speed of BacT/Alert Virtuo’s automated closed system and advanced algorithms compared to BacT/Alert 3D (Babowicz et al., 2021).

Notably, antimicrobial treatment duration remained unchanged post-optimization (*P* > 0.05), likely attributable to the clinical necessity for prolonged therapy of severe BSI and the masking effect of empirical broad-spectrum regimens. Nevertheless, antimicrobial costs decreased significantly from ¥3,828.5 to ¥3,127.27, reflecting earlier targeted therapy implementation. Reduced transport delays minimized therapeutic optimization lag time, consistent with Yvan Caspar’s findings (Caspar et al., 2024). Total hospitalization costs showed a modest reduction from ¥44,242.4 to ¥41,748.5, suggesting limited but measurable health economic impacts of the implementation off BacT/Alert Virtuo.

During phase III, we innovatively integrated VITEK 2 XL’s AES with MIC distribution-based fingerprinting technology to characterize precisely bacterial resistance phenotypes from short-term cultures. Coupled with the ART for susceptibility report verification, this achieved automatic pre-reporting to clinicians, reducing TAT to < 2 days (47.2 h). Without extending staff hours, earlier availability of susceptibility data for common antimicrobials enabled timely therapeutic adjustments, significantly shortening empirical treatment duration (15.0 d vs. 11.5 d, *P* < 0.05). Post-optimization laboratory testing costs, antimicrobial expenditures and total hospitalization expenses all demonstrated marked reductions. Existing evidence confirms that targeted antibiotic therapy success directly correlates with rapid susceptibility reporting, as delayed results reduced the modification likelihood of initial regimens (Halperin et al., 2022). These findings underscore the critical importance of expedited AST in stewardship programs.

Our findings revealed that despite implementing three distinct optimization measures, no significant differences emerged in total hospitalization duration across the intervention phases. These results align with the observations of Ai et al (Ai et al., 2024), but contrast with the study of Dai et al. which reported reduced hospitalization through BC workflow improvements (Dai et al., 2024). This discrepancy may stem from the rapid progression and high mortality of BSI, which compel clinicians to initiate broad-spectrum empirical therapy early (Babowicz et al., 2021; Kim et al., 2024), potentially obscuring length-of-stay variations. However, prolonged empirical broad-spectrum regimens risk selecting resistant pathogens, necessitating strict duration control (Adamik et al., 2020). Evidence highlights the critical balance between aggressive empirical therapy and timely de-escalation for optimizing outcomes (Babowicz et al., 2021), where microbial identification and AST form the cornerstone of stewardship-driven therapy adjustments. Although mortality reductions across the three phases (25.30% vs. 17.78%, 31.33% vs. 24.36%, 24.36% vs. 13.33%) lacked statistical significance, these trends mirror prognostic patterns reported by Ai et al. and Dai et al (Ai et al., 2024; Dai et al., 2024). Notably, multiple studies have emphasized that expedited positive BC reporting is pivotal for optimizing BSI management, shortening hospitalization, improving prognosis and reducing costs (Dunbar et al., 2022; Fabre et al., 2022; Halperin et al., 2022).

The present study had some limitations. First, its single-center design and modest sample size limit generalizability to diverse healthcare settings, necessitating multicenter validation. Second, residual methodological constraints persist despite workflow optimizations. For instance, certain Gram-positive cocci remain challenging to speciate even after short-term incubation (Nomura et al., 2020), and MALDI-TOF struggles with polymicrobial culture identification (Nomura et al., 2020). Future advancements in rapid diagnostics may address these technical barriers.

In summary, we sequentially implemented three interventions: MALDI-TOF for rapid pathogen identification; BacT/Alert Virtuo for 24/7 automated bottle loading; and VITEK 2 XL’s AES with automated susceptibility pre-reporting. Quantitative analysis of these optimizations elucidated their differential impacts on BC workflows and prognostic determinants. Continuous improvement of microbiology laboratory processes can enable MIC reports to clinicians within 48 hours. While hospitalization metrics remained stable, mortality trends and cost reductions underscore the clinical value of accelerated diagnostics. These findings advocate for laboratory innovation as a driver of antimicrobial stewardship and precision medicine in the management of BSI.

## Data Availability

The original contributions presented in the study are included in the article/**Supplementary Material**. Further inquiries can be directed to the corresponding author/s.
