# Supplementary material for: Multifactorial impacts of blood culture process optimization on clinical outcomes and healthcare economics in bloodstream infection management
Source: Front Cell Infect Microbiol. 2026 Mar 13;16:1699905. doi: 10.3389/fcimb.2026.1699905 (PMC13021603; doi:10.3389/fcimb.2026.1699905)
Supplement: Supplementary file 1 [file DataSheet1.pdf]

Table 1 Specimen information of Phase I Pre-op

ID	Admission number	Gender	Age	WBC (10^9/L)	Neutrophil (10^9/L)	CRP (μg/mL)	PCT (ng/mL)	Admission date	Date of discharge	Organism	Clinical outcomes	Total hospitalization costs	Total laboratory costs	Total antibacterial agents costs	Length of hospital stay
1	2574911	F	69	12.21	11.66	219.84	37.27	2023/6/9	2023/6/27	*Klebsiella pneumoniae*	Death	147422.48	28856.3	7511.49	18
2	423856	F	77	16.11	14.74	158.74	0.401	2023/8/13	2023/9/4	*Non-fermented rods*	Recovery	109129.99	14604	12776.19	22
3	2572959	F	67	4.22	2.85	18.21	0.107	2023/6/5	2023/7/5	*Acinetobacter baumannii*	Recovery	121745.41	11686.3	3380.72	30
6	2585263	M	62	23.77	22.65	72.29	55.66	2023/7/2	2023/7/25	*Candida glabra*	Death	455024.11	44569.8	52422.86	23
8	2486645	F	37	15.10	14.32	40.91	3.13	2023/6/16	2023/7/14	*Acinetobacter baumannii*	Death	453618.65	41109.6	46015.16	28
10	1061373	M	60	7.22	6.48	322.74	89.48	2023/7/24	2023/8/4	*Staphylococcus aureus*	Death	134472.17	12156.5	2258.49	11
12	429943	M	94	9.32	7.71	110.91	8.77	2023/3/28	2023/6/19	*Escherichia coli*	Death	220674.44	15345.3	7450	83
13	2608003	M	49	4.37	2.98	186.87	25.37	2023/8/14	2023/10/13	*Candida glabra*	Death	554368.52	37956.9	95082.35	60
14	2587009	M	72	12.19	10.71	NA	4.72	2023/7/5	2023/8/16	*Mycoplasma hominis*	Death	332682.65	49637.8	22640.07	42
15	2068125	M	54	15.55	12.78	NA	0.404	2023/7/28	2023/8/30	*Staphylococcus haemolyticus*	Death	108343.5	10753.9	3569.76	33
17	2600529	M	69	2.4	1.99	173.29	1.86	2023/7/30	2023/9/3	*Klebsiella pneumoniae*	Death	309149.65	31957.4	55441.55	35
19	583280	F	76	11.09	9.57	NA	16.41	2023/8/2	2023/8/20	*Staphylococcus aureus*	Recovery	50797.16	11826.5	1649.06	18
22	1936185	M	64	17.84	16.42	NA	0.655	2023/7/19	2023/7/28	*Staphylococcus hominis*	Death	71193.94	12868	12205.79	9
23	2156877	M	36	9.13	8.44	5.91	0.063	2023/5/23	2023/7/24	*Staphylococcus capitis*	Death	243278.18	26553.3	22211	62
24	1463379	M	91	12.97	11.91	NA	6.2	2023/5/14	2023/6/9	*Staphylococcus hominis*	Death	181340.11	28599.4	7359.55	26
25	1344868	F	83	33.85	32.7	247.96	43.71	2023/7/27	2023/8/6	*Staphylococcus haemolyticus*	Death	123088.22	21724.7	3445.75	10
26	2585235	M	74	10.83	10.1	161.54	0.388	2023/7/2	2023/8/23	*Acinetobacter baumannii*	Death	259767	28044.3	51315.44	52
28	2578282	M	29	54.42	50.83	367.94	1.94	2023/6/17	2023/7/10	*Staphylococcus aureus*	Death	65668.43	9139.5	13352.34	23
29	2346870	M	53	12.24	11.41	23.5	3.85	2023/6/21	2023/7/7	*Klebsiella pneumoniae*	Recovery	64527.02	11980.5	2069.35	16
30	472318	M	93	6.64	5.49	133.62	0.329	2023/5/18	2023/6/15	*Pseudomonas aeruginosa*	Death	80438.17	8725.3	2769.25	28
31	2608109	F	60	20.91	18.95	290.5	88.23	2023/8/14	2023/8/19	*Streptococcal pharyngitis*	Death	19953.73	6914.5	490	5
32	2194718	M	69	8.71	7.36	74.7	0.186	2023/6/14	2023/10/6	*Staphylococcus hominis*	Recovery	376147.72	66967.4	25214.17	114
33	2591402	M	79	8.46	7.85	11.49	0.253	2023/7/12	2023/7/29	*Acinetobacter calcoaceticus*	Recovery	75320.5	8396	2834.87	17
36	2542320	M	59	44.95	43.7	149.71	100	2023/8/4	2023/8/12	*Klebsiella pneumoniae*	Recovery	36699.96	4918.8	1925.84	8
37	2474821	M	75	13.97	13.44	25.86	1.48	2023/8/17	2023/8/23	*Streptococcal pharyngitis*	Recovery	35714.12	4671	3185.9	6
38	2298599	M	82	13.32	12.34	54.28	24.84	2023/6/3	2023/6/7	*Enterococcus faecium*	Recovery	25728.94	3568	29.47	4

39	713080	F	85	13.44	11.98	221.65	0.056	2023/8/21	2023/9/5	*Staphylococcus epidermidis*	Recovery	44276.29	6684.3	6145.41	15
40	2569136	F	67	9.75	7.52	71.65	0.767	2023/5/29	2023/6/25	*Candida tropicalis*	Recovery	80735.52	26199.3	25899.18	27
41	2249108	M	61	5.87	4.52	1.95	0.132	2023/8/1	2023/8/12	*Aeromonas veronii*	Recovery	22799.84	4904.3	1365.72	11
42	2605502	F	65	12.10	11.48	74.79	7.73	2023/8/9	2023/8/15	*Escherichia coli*	Recovery	32479.03	3708	574.91	6
44	2595657	F	71	10.51	9.26	71.58	2.01	2023/7/19	2023/7/26	*Staphylococcus hominis*	Recovery	14567.01	4425.5	1582.13	7
45	2615009	F	55	14.89	14.16	53.67	8.51	2023/8/27	2023/9/1	*Escherichia coli*	Recovery	30352.07	3644	1486.28	5
46	2610657	M	83	16.17	15.77	18.29	52.53	2023/8/17	2023/8/22	*Escherichia coli*	Recovery	44021.92	4114.5	1260.25	5
47	2232830	M	67	3.43	2.24	NA	0.151	2023/8/4	2023/8/12	*Staphylococcus hominis*	Recovery	23595.55	5209.5	1583.2	8
48	2531913	F	65	5.58	4.84	36.27	6.72	2023/8/26	2023/8/31	*Staphylococcus hominis*	Recovery	25540.84	4235	1074.87	5
49	2585279	M	80	6.17	4.24	110.5	1.11	2023/7/3	2023/7/10	*Staphylococcus epidermidis*	Recovery	11477.14	3667	605.93	7
50	2282373	M	70	4.71	3.26	23	0.312	2023/6/10	2023/6/17	*Shewanella algae*	Recovery	33232.75	4221.8	551.42	7
51	2586841	M	84	13.81	12.96	225.76	19.13	2023/7/5	2023/7/18	*Escherichia coli*	Recovery	44481.9	6666.5	4936.38	13
52	2555325	F	70	7	5.34	42.11	0.106	2023/6/7	2023/6/22	*Staphylococcus hominis*	Recovery	21416.05	4810	92.43	15
53	2206763	F	71	13.67	12.94	1.26	0.364	2023/7/12	2023/7/18	*Escherichia coli*	Recovery	32097.46	3599	594.08	6
54	1430146	F	86	6.17	4.69	7.48	0.987	2023/6/28	2023/7/4	*Escherichia coli*	Recovery	27713.44	4143	498.08	6
55	2587007	M	64	5.23	3.92	6.97	0.049	2023/7/5	2023/7/18	*Klebsiella pneumoniae*	Recovery	20521.13	5610.5	298.46	13
56	2603450	M	60	19.94	18.28	205.06	0.278	2023/8/4	2023/8/18	*Staphylococcus hominis*	Recovery	22407.69	6096.5	4668.8	14
57	1156225	F	81	9.08	8.33	34.35	18.36	2023/6/26	2023/7/4	*Escherichia coli*	Recovery	30832.38	4703.5	643.5	8
58	2585739	F	83	6.25	5.2	32.96	0.101	2023/8/10	2023/8/15	*Klebsiella aerogenes*	Recovery	27858.52	2542	2663.87	5
59	1854120	F	89	17.9	16.18	283.81	9.03	2023/7/22	2023/7/26	*Escherichia coli*	Recovery	33915.77	3840	2279.71	4
61	2019574	M	75	23.46	24.8	96.41	7.33	2023/6/27	2023/6/30	*Klebsiella pneumoniae*	Recovery	25016.13	3008	1053.5	3
62	2505503	M	76	13.67	11.54	NA	NA	2023/7/18	2023/7/26	*Escherichia coli*	Recovery	32530.66	3055.8	1311.58	8
63	2612806	F	56	4.31	2.41	5.64	0.238	2023/8/22	2023/9/5	*Staphylococcus capitis*	Recovery	16559.78	7205.8	61.62	14
64	2587660	M	43	7.29	6.73	297.88	0.832	2023/7/7	2023/7/24	*Klebsiella pneumoniae*	Recovery	28695.79	7130.1	7212.71	17
65	2602235	F	56	4.33	3.01	0.13	NA	2023/8/17	2023/8/31	*Micrococcus luteus*	Recovery	15078.19	1340.5	14.31	14
66	1140735	M	85	17.92	17.41	170.62	52.23	2023/7/16	2023/7/24	*Escherichia coli*	Recovery	38044.28	4899.3	1194.21	8
67	2600482	M	65	5.09	4.26	39.67	1.6	2023/7/30	2023/8/8	*Salmonella enteritidis*	Recovery	37283.2	8207.3	1526.05	9
68	2611726	M	72	6.86	5.87	3.24	0.128	2023/8/20	2023/8/31	*Aeromonas caviae*	Recovery	10248.58	3646.3	803.52	11
69	778031	M	71	10	9.52	1.19	0.104	2023/7/8	2023/7/22	*Aeromonas veronii*	Recovery	56422.89	7285.5	6081.33	14
70	2598061	M	69	10.07	9.22	4.33	0.604	2023/7/24	2023/8/1	*Clostridium perfringens*	Recovery	15131.43	3889.5	1935.05	8
71	2432655	M	66	17.81	17.39	203.31	100	2023/8/5	2023/8/16	*Staphylococcus epidermidis*	Recovery	33132.32	5957.3	2431.81	11
72	2610906	M	87	12.45	11.7	283.08	2.07	2023/8/18	2023/8/25	*Pseudomonas aeruginosa*	Recovery	35760.68	4491.5	1727.96	7

73	2453744	M	63	12.89	12.05	200.06	38.47	2023/7/23	2023/7/29	*Klebsiella pneumoniae*	Recovery	25886.88	5412.5	677.98	6
74	2616201	F	68	0.52	0.03	270.03	NA	2023/8/30	2023/9/11	*Staphylococcus hominis*	Recovery	40412.27	817.5	1473.11	12
75	2603193	F	85	18.71	18.04	19.28	12.12	2023/8/3	2023/8/12	*Escherichia coli*	Recovery	27488.35	4267	3123.56	9
76	2613342	M	45	34.32	31.4	131.2	31.79	2023/8/23	2023/9/5	*Escherichia coli*	Recovery	33462.64	9976.3	5207.75	13
77	2230416	M	60	8.03	7.07	40.71	0.438	2023/8/21	2023/9/4	*Escherichia coli*	Recovery	38088.17	5678.3	4387.41	14
78	333170	M	60	7.68	6.26	112.41	0.289	2023/7/5	2023/7/15	*Streptococcus salivarius*	Recovery	16998.65	3693.8	504.14	10
79	1673013	M	60	7.69	5.87	18.34	0.735	2023/7/5	2023/7/11	*Escherichia coli*	Recovery	32350.5	4045.8	1470.44	6
81	2436897	M	71	13.66	14.24	18.61	0.343	2023/8/3	2023/8/12	*Staphylococcus hominis*	Recovery	25783.61	4238	655.19	9
82	945990	F	78	21.38	20.34	130.9	14.41	2023/8/11	2023/8/17	*Escherichia coli*	Recovery	31053.54	3871.5	1563.73	6
83	2614276	M	73	14.76	13.92	223.03	100	2023/8/25	2023/9/7	*Enterococcus faecium*	Recovery	39417.9	7225	2376.16	13
85	2587470	M	90	22.91	21.9	58.03	7.12	2023/7/6	2023/7/13	*Escherichia coli*	Recovery	32396.01	4727.5	2870.9	7
86	2583117	M	59	7.13	5.85	NA	NA	2023/6/27	2023/7/8	*Streptococcus mitis*	Recovery	35513.09	6600.8	85.89	11
87	2587936	M	73	6.36	5.51	151.38	1.12	2023/7/7	2023/7/18	*Escherichia coli*	Recovery	15856.41	5528.8	332.95	11
88	2168475	M	74	5.07	4.78	36.82	28.27	2023/6/28	2023/7/7	*Escherichia coli*	Recovery	34901.39	4307	3916.51	9
90	2615964	M	54	12.23	10.88	89.06	11.26	2023/8/29	2023/9/20	*Escherichia coli*	Recovery	74652.02	6838.8	4431.97	22
91	2575080	F	79	8.68	7.78	141.02	36.83	2023/6/10	2023/6/18	*Staphylococcus hominis*	Death	20721.59	4223.5	3867.57	8
92	1256506	M	56	8.84	7.17	NA	NA	2023/8/12	2023/8/15	*Escherichia coli*	Death	7664.78	3163	140.71	3
93	2571625	F	90	15.6	14	281.1	0.598	2023/6/2	2023/6/9	*Staphylococcus hominis*	Recovery	12351.47	3054	2088.65	7
94	2600529	M	69	6.89	6.34	234.47	1.86	2023/7/30	2023/9/3	*Klebsiella pneumoniae*	Death	309149.65	31957.4	55441.55	35
95	2599494	M	39	20.35	19.03	325.73	7.9	2023/7/27	2023/8/3	*Staphylococcus aureus*	Death	36738.55	9047.5	3470.87	7
96	835333	M	70	7.64	5.81	102.38	0.372	2023/6/30	2023/7/8	*Escherichia coli*	Recovery	11529.11	4006.8	1715.37	8
97	761061	F	45	18.16	17.14	208.57	56.5	2023/8/7	2023/8/14	*Klebsiella pneumoniae*	Recovery	15316.36	6012	2204.86	7
98	2577499	F	67	11.94	10.85	62.78	3.43	2023/6/15	2023/7/10	*Enterococcus faecalis*	Recovery	73990.09	9527.8	6575.23	25
99	601261	M	59	5.16	4.79	NA	3.08	2023/7/24	2023/8/10	*Klebsiella oxytoca*	Recovery	78042.16	7843.3	3989.99	17
100	2585522	M	73	13.6	12.06	NA	2.17	2023/7/3	2023/7/9	*Klebsiella pneumoniae*	Recovery	15785.28	3771.8	2060.19	6

Table 2 Specimen information of Phase I Post-op

ID	Admission number	Gender	Age	WBC (10^9/L)	Neutrophil (10^9/L)	CRP (μg/mL)	PCT (ng/mL)	Admission date	Date of discharge	Organism	Clinical outcomes	Total hospitalization costs	Total laboratory costs	Total antibacterial agents costs	Length of hospital stay
1	2133796	F	53	9.03	5.43	2.51	0.102	2023/12/22	2024/2/15	*Escherichia coli*	Recovery	196845.86	32720.3	22807.2	55
3	2643805	M	67	20.18	18.73	257.51	50.15	2023/11/4	2023/11/9	*Klebsiella pneumoniae*	Death	83660.14	15629.8	6084.9	5
4	2648162	F	61	16.06	15.33	118.84	77.3	2023/11/13	2023/11/23	*Escherichia coli*	Recovery	23371.35	4514.5	2890.77	10
6	2629935	M	46	20.56	15.95	8.24	0.139	2023/10/5	2023/10/24	*Aeromonas veronii*	Recovery	35885.81	9361.5	1209.24	19
7	2659898	M	48	3.89	3.12	323.54	NA	2023/12/10	2024/1/21	*Staphylococcus aureus*	Death	835973.33	89134.5	88742.8	42
8	2321424	M	56	6.64	4.61	NA	0.5	2023/5/30	2023/12/26	*Enterococcus faecalis*	Death	755296.11	72888.7	114135.12	210
9	2650342	M	72	12.23	11.49	18.68	5.61	2023/11/18	2023/11/24	*Enterococcus faecalis*	Death	49682.3	8352.5	2418.13	6
10	885820	M	77	14.37	12.3	167.47	0.505	2023/9/23	2023/10/24	*Acinetobacter baumannii*	Death	223078.79	41782.3	24154.46	31
11	1147461	F	67	3.59	3.37	48.26	3.07	2023/11/22	2023/12/27	*Pseudomonas aeruginosa*	Death	207667.76	22494.8	27528.22	35
13	1294946	F	65	3.96	2.05	46.26	1.93	2023/8/29	2023/12/8	*Klebsiella pneumoniae*	Death	1857263.63	109682.1	172507.57	101
14	2591640	M	90	6.99	3.97	NA	0.068	2023/11/21	2023/12/26	*Staphylococcus hominis*	Death	100832.12	14527.1	5743.89	35
15	2111820	M	59	6.86	5.87	NA	3.5	2023/9/12	2023/10/26	*Staphylococcus hominis*	Death	411188.18	57581.8	54170.03	44
16	2655692	M	56	9.54	8.85	45.98	0.064	2023/11/30	2024/1/6	*Staphylococcus epidermidis*	Recovery	227636.2	17559	13142.95	37
17	400634	M	57	2.55	2.19	24.29	0.052	2023/11/1	2023/11/7	*Streptococcus constellatus*	Recovery	7565.14	4059.5	606.66	6
18	2200718	M	74	8.23	7.22	43.09	0.378	2023/11/13	2023/12/2	*Staphylococcus hominis*	Recovery	22477.94	3980.5	139.76	19
19	1664630	M	88	14.68	14.35	63.35	1.62	2023/12/20	2023/12/27	*Escherichia coli*	Recovery	32053.8	3606	2356.5	7
20	2631981	M	75	26.42	23.03	102.79	8.44	2023/10/9	2023/10/18	*Pseudomonas aeruginosa*	Recovery	31587.75	3305.5	2936.89	9
21	2360965	M	71	7.32	6.8	2.39	0.109	2023/12/2	2023/12/23	*Streptococcus salivarius*	Recovery	26077.92	4862.3	2638.24	21
23	2629312	M	45	12.14	11.39	343.05	29.43	2023/10/3	2023/10/12	*Klebsiella pneumoniae*	Recovery	15708.19	5035.5	3234.76	9
24	2665360	M	70	21.27	20.29	67.06	0.657	2023/12/20	2023/12/27	*Enterococcus faecium*	Recovery	32558.06	3754.5	2099.62	7
25	2632474	F	72	15.2	14.3	285.01	1.23	2023/10/10	2023/10/18	*Escherichia coli*	Recovery	27409.14	3679.5	3866.77	8
26	2636564	M	76	7.16	6.42	222.3	1.96	2023/10/19	2023/10/25	*Escherichia coli*	Recovery	34299.19	4165	3046.89	6
27	2626225	F	65	9.81	7.86	57.11	0.69	2023/11/3	2023/11/8	*Escherichia coli*	Recovery	30420.79	3685.5	393.2	5
28	1681995	M	50	15.23	11.66	18.96	0.479	2023/11/3	2023/11/16	*Streptococcus agalactiae*	Recovery	21807.86	3335.8	724.91	13
29	487966	M	81	6.46	5.55	98.01	3.31	2023/11/27	2023/12/2	*Enterococcus faecalis*	Recovery	25487.72	3074	375.53	5
31	2644482	M	60	8.49	6.95	6.29	3.76	2023/11/6	2023/11/11	*Klebsiella pneumoniae*	Recovery	23076.02	1772.5	480.11	5

32	2335924	M	54	3.68	3.11	39.3	0.286	2023/9/26	2023/10/16	*Escherichia coli*	Recovery	29392.4	4533.5	881.4	20
33	2628903	M	90	16.79	15.66	5.49	2.14	2023/10/2	2023/10/10	*Achromobacter denitrificans*	Recovery	34451.07	2401	3289.33	8
34	1313730	M	83	6.92	5.74	21.51	0.287	2023/11/21	2023/11/30	*Streptococcus mitis*	Recovery	32753.39	5964.3	685.4	9
35	2505503	M	76	8.72	7.22	286.56	3.26	2023/10/4	2023/10/15	*Escherichia coli*	Recovery	14712.95	4775.5	721.27	11
36	2488902	M	61	7.1	6.52	9.95	0.045	2023/11/21	2023/11/27	*Escherichia coli*	Recovery	27324.15	3579.3	553.84	6
37	2631410	M	54	11.04	9.34	10.46	0.18	2023/10/8	2023/10/22	*Staphylococcus capitis*	Recovery	28440.54	3864.3	756.54	14
38	2645140	F	52	11.25	9.57	68.74	5.56	2023/11/30	2023/12/4	*Klebsiella pneumoniae*	Recovery	8894.02	4368.8	1046.71	4
39	754375	F	66	13.67	13.15	16.23	NA	2023/12/13	2023/12/18	*Klebsiella pneumoniae*	Recovery	3032.73	3420.5	569.14	5
40	393660	M	92	15.29	14.71	15.8	1.55	2023/11/13	2023/11/20	*Escherichia coli*	Recovery	28362.68	4220	627.57	7
41	2476586	M	71	13.97	13.11	NA	7.97	2023/10/16	2023/10/20	*Escherichia coli*	Recovery	25517.57	3308	485.08	4
42	1755757	M	68	13.94	11.91	10.71	0.132	2023/11/12	2023/11/16	*staphylococcus cohnii*	Death	59550.18	14018.3	1058.82	4
43	2662048	M	66	11.53	11.15	3.26	1.45	2023/12/12	2023/12/18	*Streptococcus constellatus*	Recovery	38402.93	4311.3	504.57	6
44	2265042	M	27	8.92	7.31	160.21	0.348	2023/10/18	2023/10/25	*Staphylococcus hominis*	Recovery	16143.43	4434	1702.13	7
45	2654459	F	71	9.82	7.89	0.54	0.048	2023/11/27	2023/12/2	*Escherichia coli*	Recovery	31031.19	4254	402.25	5
46	1959285	M	44	4.67	3.39	NA	NA	2023/10/6	2023/10/20	*Staphylococcus hominis*	Recovery	42547.12	5597.8	303.78	14
47	2648276	M	82	2.54	0.15	87.67	38.19	2023/11/13	2023/11/20	*Escherichia coli*	Recovery	31635.04	3763	321.83	7
48	1386035	M	74	12.1	11.26	23.2	5.57	2023/12/4	2023/12/11	*Escherichia coli*	Recovery	20752.99	3895	677.98	7
50	2666250	M	28	14.41	14.05	74.08	72.58	2023/12/23	2023/12/30	*Klebsiella pneumoniae*	Recovery	9089.33	2699.8	642.16	7
51	2660775	M	55	7.92	6.22	49.13	0.329	2023/12/11	2023/12/31	*Klebsiella pneumoniae*	Recovery	92064.11	6581.3	1824.96	20
52	2605343	F	88	28.18	27	82.25	30.01	2023/12/30	2024/1/7	*Enterococcus faecium*	Recovery	24410.48	6287	3170.81	8
53	1621670	M	67	5.14	4.52	340.62	1.32	2023/11/5	2023/11/10	*Escherichia coli*	Recovery	9043.72	3416.5	490	5
54	2633059	F	67	6.19	5.2	89.37	2.55	2023/10/11	2023/10/18	*Proteus mirabilis*	Recovery	7354.53	3101.5	155.8	7
55	2650338	M	68	13.76	12.81	206.55	7.33	2023/11/18	2023/11/23	*Klebsiella pneumoniae*	Recovery	9847.27	3012.8	821.94	5
56	769348	F	65	15.35	14.63	93.82	100	2023/10/13	2023/10/21	*Escherichia coli*	Recovery	13078.33	4298	3417.53	8
57	2650323	F	73	15.15	13.74	NA	52.38	2023/11/18	2023/11/25	*Klebsiella pneumoniae*	Recovery	11085.23	3572.8	2207.85	7
58	2651784	F	62	12.13	10.85	NA	42.59	2023/11/21	2023/12/14	*Klebsiella pneumoniae*	Recovery	29666.08	7059.8	6281.43	23
59	2649582	F	83	15.45	14.74	NA	100	2023/11/16	2023/11/28	*Escherichia coli*	Recovery	38694.41	9090	2273.73	12
60	2649448	F	74	10.19	9.1	85.41	2.49	2023/11/16	2023/11/30	*Klebsiella aerogenes*	Recovery	43257.42	7077.3	1036.73	14
61	2592914	M	29	23.59	20.68	NA	0.105	2023/10/17	2023/10/25	*Staphylococcus capitis*	Recovery	5877.62	1946	656.03	8
62	2664456	M	76	16.32	15.66	73.7	2.4	2023/12/18	2023/12/22	*Escherichia coli*	Recovery	5896.4	2396	382.97	4
63	2629111	F	81	6.73	1.56	NA	0.242	2023/10/3	2023/10/19	*Enterococcus casseliflavus*	Recovery	55785.59	8963	499.74	16
64	2136459	M	57	12.47	11.18	NA	64.2	2023/10/17	2023/10/25	*Staphylococcus hominis*	Recovery	21227.44	3085.3	3886.5	8

65	2543009	F	69	10.22	9.62	17.12	0.322	2023/12/11	2023/12/18	*Aeromonas caviae*	Recovery	8925.49	3951	1565.6	7
66	1634988	F	89	16.11	14.74	7.23	5.05	2023/11/5	2023/11/13	*Pseudomonas aeruginosa*	Recovery	6402.76	2603.8	1782.21	8
67	2649770	F	77	11.3	10.65	170.42	22.31	2023/11/17	2023/12/5	*Escherichia coli*	Recovery	74971.6	9897.3	3664.54	18
68	2202764	F	58	1.89	0.89	110	2.8	2023/10/19	2023/11/17	*Escherichia coli*	Recovery	35231.58	3264.5	2175.1	29
70	658510	M	70	2.57	0.11	NA	0.96	2023/10/7	2023/11/8	*Pseudomonas aeruginosa*	Recovery	68855.63	13753.8	15062.93	32
71	2637049	M	79	4.02	2.49	NA	1.45	2023/10/20	2023/11/2	*Escherichia coli*	Death	27174.84	11010.5	3677.58	13
72	2663434	F	73	19.79	17.73	NA	0.28	2023/12/15	2023/12/23	*Escherichia coli*	Death	24737.39	9369.3	1641.75	8
73	2535399	F	60	0.45	0.01	NA	0.11	2023/9/30	2023/10/14	*Klebsiella pneumoniae*	Recovery	21357.05	4625.3	589.59	14
75	2646918	M	68	3.75	2.69	NA	0.65	2023/11/10	2023/11/26	*Aeromonas veronii*	Recovery	32906.87	6584.5	4792.95	16
76	2650643	F	81	0.35	0.03	NA	0.309	2023/11/19	2023/12/18	*Escherichia coli*	Recovery	66648.18	15056.8	12477.23	29
77	667481	F	60	0.21	0.01	8.08	0.141	2023/9/29	2023/10/31	*Klebsiella pneumoniae*	Recovery	69649.59	14871.5	20925.3	32
78	2650223	F	75	3.8	1.5	NA	NA	2023/11/18	2023/12/16	*Micrococcus luteus*	Recovery	36433.59	14712.3	1172.68	28
79	2554300	M	43	0.26	0.24	229	NA	2023/11/25	2023/12/12	*Bacteroides polymorphus*	Death	44772.1	3263.1	21044.41	17
80	2533844	F	39	10.1	9.33	39.5	0.29	2023/11/11	2023/11/29	*Enterococcus faecium*	Recovery	39563.93	4870.3	9968.95	18
82	2329849	M	47	0.43	0.23	NA	NA	2023/11/30	2023/12/26	*listeria monocytogenes*	Recovery	32178.7	9375	7290.01	26
83	1666952	M	80	24.73	20.45	167.1	0.555	2023/11/25	2023/11/30	*Pseudomonas aeruginosa*	Death	10517.53	3338	459.66	5
84	1775531	M	66	8.33	7.84	282.24	100	2023/10/18	2023/10/23	*Escherichia coli*	Recovery	11694.14	3522.5	1052.74	5
85	2390590	M	71	22.34	21.39	178.84	NA	2023/10/23	2023/11/2	*Enterococcus faecium*	Recovery	30986.17	3107.8	1639.25	10
86	2628828	F	73	10.08	9.13	234.13	7.95	2023/10/1	2023/10/9	*Escherichia coli*	Recovery	16127.69	4200.3	1206.31	8
88	2629125	M	68	10.89	10.01	8.02	0.046	2023/10/3	2023/10/24	*Pseudomonas aeruginosa*	Recovery	45888.33	4615.5	570.8	21
89	2622400	M	75	31.46	30.97	59.08	0.221	2023/9/14	2023/10/21	*Enterococcus faecium*	Recovery	85132.02	8597.5	4204.01	37
90	2641961	M	79	3.61	2.31	67.19	0.155	2023/10/31	2023/11/17	*Acinetobacter baumannii*	Recovery	64665.56	4002.8	729.78	17
91	2439508	M	65	2.51	2.05	161.98	1.89	2023/9/18	2023/10/18	*Candida albicans*	Recovery	92837.17	7217	20513.06	30
92	2539361	F	73	7.03	5.29	41.18	41.18	2023/11/24	2023/12/11	*Klebsiella pneumoniae*	Recovery	19274.28	3993.1	284.18	17
93	1066771	F	78	5.2	4.77	40.56	12.3	2023/11/6	2023/11/20	*Escherichia coli*	Recovery	16469.73	7766.3	807.6	14
94	2542942	M	55	11.88	10.39	228.79	9.68	2023/10/30	2023/11/5	*Staphylococcus aureus*	Death	23652.94	6179.5	3041.89	6
95	2631259	M	77	7.45	5.98	NA	0.417	2023/10/8	2023/10/25	*Staphylococcus epidermidis*	Recovery	33735.77	7329.8	466.76	17
96	905588	F	67	5.92	4.85	NA	0.811	2023/11/17	2023/12/2	*Enterococcus faecalis*	Recovery	19934.72	8806.1	1152.19	15
97	1869874	F	72	19.96	17.66	61.6	0.455	2023/9/21	2023/11/19	*Staphylococcus aureus*	Death	180845.33	21959.5	2451.74	59
98	2663227	M	10	11.95	8.84	154.94	0.221	2023/12/15	2023/12/25	*Staphylococcus aureus*	Recovery	4516.21	1656.5	426.24	10
99	2665316	M	6	6.79	4.53	54.06	0.501	2023/12/20	2023/12/26	*Staphylococcus epidermidis*	Recovery	5303.15	3270.3	51.76	6
100	2154602	M	71	13.78	13.21	75.82	1.83	2023/10/16	2023/12/25	*Pseudomonas aeruginosa*	Recovery	170625.63	9808	8430.15	70

Table 3 Specimen information of Phase II Pre-op

ID	Admission number	Gender	Age	WBC (10^9/L)	Neutrophil (10^9/L)	CRP (μg/mL)	PCT (ng/mL)	Admission date	Date of discharge	Species identification	Organism	Clinical outcomes	Total hospitalization costs	Total laboratory costs	Total antibacterial agents costs	Length of hospital stay
1	2659898	M	48	3.89	3.12	323.54	NA	2023/12/10	2024/1/21	2023/12/11 14:46	*Staphylococcus aureus*	Death	835973.33	89134.5	8874.28	42
2	2660775	M	55	7.92	6.22	49.13	0.329	2023/12/11	2023/12/31	2023/12/13 8:55	*Klebsiella pneumoniae*	Recovery	92064.11	6581.3	1824.96	20
4	2665006	F	85	15.21	13.22	8.13	0.128	2023/12/19	2023/12/28	2023/12/22 14:54	*Staphylococcus hominis*	Recovery	22358.45	3745.1	1384.57	9
5	2655692	M	56	9.54	8.85	45.98	0.064	2023/11/30	2024/1/6	2023/12/22 14:55	*Staphylococcus epidermidis*	Death	227636.2	17559	13142.95	37
6	689901	F	79	20.34	18.53	115.43	0.862	2024/1/1	2024/1/11	2024/1/4 9:43	*Klebsiella pneumoniae*	Recovery	9698.07	3358.8	1186.59	10
7	554034	M	65	3.37	2.83	200.35	100	2024/1/1	2024/1/24	2024/1/6 13:57	*Enterococcus faecium*	Recovery	147027.96	19358.3	12109.07	23
8	2653098	F	63	11.79	9.36	93.88	0.244	2023/12/23	2024/2/6	2024/1/4 13:51	*Enterococcus faecium*	Death	143955.24	15701.8	22096.86	45
9	1264419	M	68	14.04	11.83	NA	17.4	2024/1/3	2024/1/20	NA	*Staphylococcus aureus*	Recovery	35461.25	7877.5	7868.39	17
10	1188427	M	72	4.51	3.13	50.91	0.647	2023/12/29	2024/1/17	NA	*Hospital Acinetobacter*	Recovery	47009.62	9472.1	748.85	19
11	703684	M	83	5.64	4.27	21.48	0.174	2024/1/4	2024/1/18	2024/1/15 8:58	*Staphylococcus haemolyticus*	Death	15266.16	1882.3	1119.72	14
12	2679696	M	55	7.71	5.84	163.08	14.79	2024/1/13	2024/1/22	2024/1/18 14:50	*Enterobacter cloacae*	Recovery	32087.58	5815.8	1885.3	9
13	2610503	M	58	0.12	0.04	3.64	10.05	2024/1/3	2024/1/26	2024/1/16 14:08	*Pseudomonas aeruginosa*	Recovery	54304.48	7824.1	4580.03	23
14	2681068	F	66	11.06	10.45	252.55	4.39	2024/1/16	2024/1/21	2024/1/18 14:00	*Enterobacter cloacae*	Recovery	13586067	3421.3	2124.3	5
15	1030285	M	85	5.72	5.54	3.74	1.48	2024/1/18	2024/1/23	2024/1/20	*Klebsiella*	Recover	10517.89	6080.8	534.27	5

										9:11	*oxytoca*	y				
17	2417240	F	65	14.16	13.41	187.69	25.87	2024/1/27	2024/5/2	2024/1/29 15:14	*Streptococcus agalactiae*	Recover y	331499.63	26543.6	12543.99	96
18	935527	M	81	10.68	9.75	128.66	0.729	2024/1/16	2024/2/22	2024/2/2 8:44	*Staphylococcu s haemolyticus Acinetobacter baumannii*	Death	123436.03	25117.4	2718.21	37
19	464237	M	59	8.13	7.81	272.77	9.48	2024/2/1	2024/2/16	2024/2/6 9:59	*Staphylococcu s aureus*	Recover y	23965.18	5688.3	2318.73	15
20	2269888	M	75	11.9	10.21	222.78	3.62	2024/2/1	2024/2/17	NA	*Klebsiella oxytoca*	Recover y	14822.92	4827.3	1660.12	16
21	690740	M	77	14.39	12.69	NA	17.78	2024/2/3	2024/2/8	2024/2/6 14:53	*Escherichia coli*	Recover y	11844.19	5122.5	461.25	5
22	2688790	F	44	10.93	9.63	NA	16.73	2024/2/4	2024/2/27	2024/2/8 8:13	*Staphylococcu s aureus*	Death	159981.73	30030.4	23575.29	23
23	2688795	M	56	9.89	9.14	93.48	8.39	2024/2/4	2024/3/4	2024/2/8 7:29	*Streptococcus parasanguis*	Recover y	140010.41	20365.8	28181.83	29
24	2688480	M	72	12.19	10.68	205.63	1.14	2024/2/3	2024/2/20	2024/2/7 13:43	*Klebsiella pneumoniae*	Recover y	38998.62	4919.3	4745.97	17
25	2011299	M	70	27.73	26.7	61.69	6.32	2024/2/5	2024/2/12	2024/2/7 13:44	*Escherichia coli*	Recover y	44242.41	4351	2342.8	7
26	2666969	F	70	0.23	0.14	45.69	0.154	2024/2/7	2024/2/20	2024/2/9 14:16	*Escherichia coli*	Recover y	23837.1	4347.3	6278.94	13
27	2689774	M	66	13.88	13.42	1.7	0.364	2024/2/8	2024/2/14	2024/2/10 9:34	*Escherichia coli*	Recover y	31603.08	4299.3	1825.61	6
28	2689867	F	70	21.03	20.1	273.81	100	2024/2/9	2024/2/17	2024/2/12 9:04	*Escherichia coli*	Death	28337.92	6128.5	929.09	8
29	609743	M	91	15.95	13.73	114.81	0.875	2024/2/5	2024/2/28	2024/2/12 13:48	*Enterococcus faecium*	Death	148024.67	25927.6	3846.4	23
30	1746477	M	58	9	8.14	40.54	1.17	2024/2/4	2024/2/18	2024/2/13 14:41	*Proteus mirabilis*	Recover y	46490.05	5684.8	396.12	14
31	2606574	M	59	10	9.05	110.58	0.366	2024/2/14	2024/2/24	2024/2/18 10:07	*Streptococcus angina*	Recover y	16222.96	4368.3	582.74	10

32	2690856	F	62	2.71	2.35	1.53	0.039	2024/2/13	2024/2/27	NA	*Streptococcus angina*	Recovery	14577.59	4556.3	849.53	14
33	2689378	F	80	28.18	24.55	105.19	1.14	2024/2/6	2024/3/5	2024/2/21 9:57	*Clostridium tertium*	Death	142148.89	16264	11935.5	28
34	2541501	F	66	11.16	8.4	27.2	0.079	2024/2/18	2024/3/24	2024/2/22 9:18	*Defective anatrophic bacteria*	Death	160294.09	18201.4	32631.95	35
35	2692911	F	72	21.45	19.2	239.76	100	2024/2/18	2024/2/26	2024/2/2314:5:21	*Escherichia coli*	Recovery	15823.76	4549	1845.67	8
36	2693547	M	92	5.41	3.85	171.17	10.12	2024/2/19	2024/2/25	2024/2/21 10:24	*Klebsiella pneumoniae*	Recovery	29853.01	4241.5	1475.68	6
37	947030	M	70	4.67	4.43	55.47	0.57	2024/2/23	2024/3/9	2024/2/25 8:55	*Klebsiella pneumoniae*	Recovery	40303.28	3983	4375.44	15
38	2696494	F	52	10.74	10.28	266.81	3.78	2024/2/26	2024/3/19	2024/2/28 9:14	*Klebsiella pneumoniae*	Recovery	41302.08	12372.5	8542.21	22
39	2685308	F	60	0.21	0.04	8.57	0.089	2024/2/29	2024/3/7	2024/3/4 14:14	*Streptococcus angina*	Recovery	15094.13	3502	987.47	7
40	1598055	M	88	6.52	5.72	89.47	1.01	2023/12/7	2024/3/22	2024/3/5 8:24	*Leuconostoc lactis*	Death	441574.01	76326.3	52831.21	106
41	2699622	F	56	16.92	14.39	NA	7.39	2024/3/4	2024/3/15	2024/3/7 9:24	*Escherichia coli*	Recovery	16571.42	5593.8	945.1	11
43	1006435	F	93	17.22	15.4	NA	2.58	2023/12/14	2024/3/21	2024/3/7 13:46	*Escherichia coli*	Death	444871.72	63244.6	7921.79	98
44	2700551	M	44	8	4.6	48.39	0.036	2024/3/6	2024/3/17	2024/3/11 10:02	*Staphylococcus hominis*	Recovery	14891.48	4452	922.4	11
46	2681024	F	67	0.52	0.09	212.23	0.173	2024/2/15	2024/3/16	2024/3/10 8:04	*Enterococcus avium*	Death	48045.58	7214.5	9590	30
47	2699627	M	67	5.39	4.94	216.86	17.2	2024/3/4	2024/8/6	2024/3/12 14:11	*Proteus mirabilis*	Recovery	245768.09	15258.5	4224.73	155
48	2447661	M	39	5.4	4.91	19.52	1.53	2024/3/2	2024/3/30	NA	*Staphylococcus aureus*	Recovery	123592.59	8995.1	1391.53	28
49	496832	M	67	11.42	9.42	40.69	0.062	2023/12/14	2024/7/8	2024/3/11 9:45	*Staphylococcus hominis*	Death	515172.18	38641.2	3877.81	207

50	2686687	M	53	0.02		128.75	0.255	2024/2/20	2024/4/4	2024/3/11 15:15	*Streptococcus oralis*	Death	235896.07	13707.5	24113.3	44
51	2699721	M	70	0.65	0.18	4.26	0.25	2024/3/5	2024/4/3	2024/3/16 9:00	*Escherichia coli*	Recovery	43010.85	13453.3	10391.12	29
52	1476119	F	71	1.73	1.14	10.52	0.031	2024/3/16	2024/3/26	NA	*Escherichia coli*	Recovery	13686.09	3080.8	4911.96	10
53	2687832	M	55	4.55	3.5	NA	0.388	2024/2/1	2024/4/24	NA	*Candida parapsilosis*	Recovery	354899.67	28592.8	56319.78	83
54	2705595	F	74	20.29	19.85	53.36	20.98	2024/3/16	2024/3/30	2024/3/18 14:24	*Klebsiella pneumoniae*	Recovery	50164.93	4989.5	3828.48	14
55	2705916	F	96	29.08	26.64	6.42	2.14	2024/3/17	2024/3/23	2024/3/20 8:55	*Enterococcus faecium*	Death	38254.65	1903	1707.55	6
56	2705825	M	62	15.61	13.35	203.43	67.46	2024/3/17	2024/4/1	2024/3/19 15:56	*Klebsiella pneumoniae*	Recovery	66611.75	12705.3	9030.19	15
57	2706316	M	70	10.39	9.52	116.98	44.82	2024/3/18	2024/3/26	NA	*Klebsiella pneumoniae*	Recovery	11094.95	3348.3	716.71	8
58	2692340	F	53	0.17	0.12	19.2	0.037	2024/3/6	2024/4/3	2024/3/21 14:08	*Streptococcus mitis*	Recovery	161918.78	14126.5	8340.48	28
59	2707798	M	57	14.94	13.3	NA	82.96	2024/3/21	2024/8/14	2024/3/23 14:21	*Klebsiella pneumoniae*	Recovery	174025.33	26715.9	22550.89	146
60	2250990	F	68	6.68	5.48	29.84	0.073	2024/3/19	2024/4/1	2024/3/24 8:43	*Klebsiella pneumoniae*	Recovery	23645.72	3896.3	656.7	13
61	2701814	F	69	0.34	0.03	7.85	0.3	2024/3/22	2024/4/1	2024/3/24 8:40	*Escherichia coli*	Recovery	15693.47	3842.3	5329.56	10
62	2314177	M	55	1.17	0.02	130.32	100	2024/3/24	2024/4/8	2024/3/25 8:32	*Escherichia coli*	Death	87470.84	8510.9	23159.43	15
64	2710060	M	44	10.54	7.99	89.77	NA	2024/3/26	2024/4/15	2024/3/29 9:05	*Candida albicans*	Recovery	97267.15	8902.8	23459.38	20
65	2659720	M	44	2.71	1.91	21.22	0.111	2024/3/25	2024/4/7	2024/3/30 8:40	*Klebsiella pneumoniae*	Recovery	18805.59	3159.3	672.48	13
66	1556422	F	66	14.88	14.11	67.97	21.98	2024/3/31	2024/4/9	2024/4/2 14:50	*Enterococcus faecium*	Recovery	11262.76	2585.8	1033.1	9

67	1383682	M	87	11.48	10.82	305.69	25.14	2024/4/1	2024/4/27	NA	*Klebsiella pneumoniae*	Death	165004.99	30052.8	9683.75	26
68	2114109	M	60	3.01	2.4	9.46	0.324	2024/4/1	2024/4/29	2024/4/4 9:10	*Enterococcus faecalis*	Death	130987.44	16186.3	53003.65	28
69	2708146	M	36	0.11	0.03	88.1	0.556	2024/3/22	2024/4/21	2024/4/4 9:05	*Escherichia coli*	Recovery	99166.03	24231.3	16629.54	30
71	2712765	M	58	8.71	7.3	NA	0.715	2024/4/1	2024/4/8	2024/4/7 9:09	*Streptococcus oralis*	Death	10396.57	3793.5	352.63	7
72	2636923	F	68	4.18	3.82	18.08	0.104	2024/3/4	2024/4/13	2024/4/7 8:59	*Staphylococcus epidermidis*	Death	46714.37	7284.3	2493.94	40
73	2716214	F	90	16.38	15.94	10.41	10.44	2024/4/9	2024/4/17	2024/4/8 8:53	*Escherichia coli*	Recovery	21718.71	4504.8	2265.81	8
74	2716190	F	53	31.93	31.49	119.2	4.07	2024/4/9	2024/4/24	2024/4/11 13:58	*Staphylococcus epidermidis*	Recovery	75311.58	7176.8	3843.69	15
76	2396543	F	79	7.3	6.8	24.21	9.55	2024/4/12	2024/4/17	2024/4/14 14:06	*Escherichia coli*	Recovery	10710.1	3943.3	943.25	5
77	2554070	M	81	14.07	11.43	NA	91.44	2024/4/14	2024/5/7	2024/4/19 14:18	*Enterococcus faecium*	Death	388537.82	42808.3	59008.24	23
78	2719470	M	84	11.71	10.61	176.24	1.04	2024/4/17	2024/6/11	2024/4/19 14:18	*Klebsiella pneumoniae*	Death	244946.18	31344	37574.64	55
79	2720334	M	63	9.52	8.93	72.05	6.6	2024/4/18	2024/4/24	2024/4/20 14:18	*Escherichia coli*	Recovery	30597.84	3836.3	577.4	6
80	2719976	F	76	10.38	8.74	NA	NA	2024/4/18	2024/4/25	2024/4/2014:20	*Staphylococcus hominis*	Recovery	29433.81	3482.3	490	7
81	2713841	M	42	5.48	3.58	17.3	0.083	2024/4/4	2024/5/8	NA	*Staphylococcus epidermidis*	Recovery	162309.06	29261.3	21106.26	34
82	1533443	M	67	20.24	19.7	146.44	54.74	2024/4/20	2024/4/26	2024/4/22 9:31	*Klebsiella pneumoniae*	Recovery	9503.09	2710.8	625.69	6
83	2720995	M	71	20.59	19.46	12.82	0.143	2024/4/20	2024/4/25	2024/4/23 13:41	*Klebsiella pneumoniae*	Death	17537.59	5899.5	874.58	5
84	668141	M	70	0.5	0.17	125.59	0.901	2024/4/22	2024/5/21	2024/4/24 14:18	*Pseudomonas aeruginosa*	Recovery	60088.87	18538.6	17638.55	29

85	2648811	F	71	4.32	3.47	151.84	0.325	2024/4/22	2024/5/9	2024/4/24 14:17	*Escherichia coli*	Death	26163.09	4600.8	5676.77	17
86	2653445	F	61	5.06	4.16	156.33	0.541	2024/4/12	2024/5/11	2024/4/27 13:59	*Escherichia coli*	Recovery	106910.62	9919	4358.95	29
87	2723150	F	86	12.15	9.59	13.46	0.208	2024/4/25	2024/5/3	2024/4/27 8:19	*Streptococcus gallinolyticus*	Death	35810.99	9551	3533.88	8
88	2723511	M	54	8.52	5.86	73.7	0.14	2024/4/26	2024/5/10	NA	*Staphylococcus aureus*	Recovery	14720.38	4137.3	2334.19	14
89	2724210	F	78	23.89	22.43	150.64	77.88	2024/4/28	2024/5/10	2024/5/1 14:56	*Escherichia coli*	Recovery	17376.1	5840.3	3045.09	12
90	2721700	M	67	8.7	7.36	49.82	0.162	2024/4/22	2024/6/20	2024/5/1 9:30	*Enterobacter cloacae*	Recovery	166504.94	10804.5	902	59

Table 4 Specimen information of Phase II Post-op

ID	Admission number	Gender	Age	WBC (10^9/L)	Neutrophil (10^9/L)	CRP (mg/L)	PCT (ng/mL)	Admission date	Date of discharge	Species identification	Organism	Clinical outcomes	Total hospitalization costs	Total laboratory costs	Total antibacterial agents costs	Length of hospital stay
1	2727581	M	81	24.52	22.14	21.22	0.475	2024/5/8	2024/5/11	2024/5/9 14:52	*Escherichia coli*	Death	24389.23	8317.8	165.16	3
2	1515608	M	60	21.65	18.58	NA	1.61	2024/5/7	2024/5/18	2024/5/12 9:01	*Staphylococcus epidermidis*	Death	72370.46	16994.1	2367.25	11
3	2726402	M	53	2.81	2.56	30.63	0.247	2024/5/5	2024/5/20	NA	*Streptococcus angina*	Recovery	22274.23	5277.5	375.6	15
4	2068125	M	55	10.32	8.61	134.62	0.27	2024/3/22	2024/5/20	2024/5/14 9:05	*Enterococcus faecium*	Death	288404.4	31616.4	56208.17	59
5	2729973	F	66	15.55	13.5	NA	0.296	2024/5/13	2024/5/20	2024/5/15 9:06	*Escherichia coli*	Recovery	18261.78	6563.5	3805.09	7
6	2605903	M	54	0.24	0.17	12.47	0.064	2024/5/5	2024/5/24	2024/5/14 14:51	*Escherichia coli*	Recovery	64579.71	8595.1	11264.98	19
7	2114109	M	60	4.90	4.14	16.58	0.114	2024/4/29	2024/5/27	NA	*Enterococcus faecalis*	Recovery	69856.62	8264.3	40056.83	28
8	2731034	M	81	29.44	28.03	243.29	7.04	2024/5/15	2024/5/25	2024/5/16 16:06	*Klebsiella pneumoniae*	Recovery	12037.05	5176	748.5	10
9	2730233	F	69	9.37	8	83.81	0.099	2024/5/14	2024/6/5	2024/5/18 9:36	*Klebsiella pneumoniae*	Recovery	18638.71	5403.8	1123.71	22
10	2098329	F	60	21.67	20.32	324.84	16.22	2024/5/16	2024/6/14	2024/5/18 9:27	*Staphylococcus aureus*	Recovery	69210.74	11743.8	16341.56	29
11	2730092	F	37	12.84	11.51	134.58	0.314	2024/5/13	2024/5/25	2024/5/19 8:42	*Escherichia coli*	Recovery	17367.07	3525.5	376.76	12
12	2732113	F	78	10.02	8.59	252.58	0.337	2024/5/18	2024/6/5	2024/5/21 10:39	*Staphylococcus hominis*	Recovery	39757.14	11004.1	2402.83	18
13	1674759	F	62	2.35	1.83	NA	NA	2024/5/20	2024/5/28	2024/5/24 9:42	*Staphylococcus hominis*	Recovery	40358.16	6494	0	8
14	2733037	F	43	20.25	17.03	223.27	2.01	2024/5/20	2024/6/4	2024/5/24	*Staphylococcu*	Recover	31376.76	7794.8	4251.57	15

										9:12	*s aureus*	y				
15	2726465	M	40	28.69	26.48	152.4	14.59	2024/5/5	2024/6/30	2024/5/23 14:18	*Enterococcus faecium*	Death	632516.18	71808.5	43795.98	56
16	2733717	F	65	11.67	9.65	273.64	2.6	2024/5/22	2024/6/3	2024/5/25 10:36	*Klebsiella pneumoniae*	Recovery	26647.6	5083	2559.53	12
17	677708	F	67	8.73	7.61	161.62	5.75	2024/5/25	2024/5/29	2024/5/27 9:22	*Morganella Morganella*	Recovery	26544.69	3194.8	470.62	4
20	534939	F	89	8.60	7.8	NA	0.819	2024/5/27	2024/6/6	2024/5/29 9:43	*Escherichia coli*	Recovery	22348.99	9381.1	2763.17	10
21	2736301	M	92	8.21	5.8	39.92	0.434	2024/5/28	2024/6/3	2024/5/30 8:23	*Enterococcus casseliflavus*	Recovery	27999.41	4803	906.48	6
22	2737127	M	73	23.30	21.97	59.85	0.251	2024/5/30	2024/7/14	2024/6/1 11:32	*Staphylococcus aureus*	Recovery	55272.5	9932.2	21945.9	45
23	2735922	M	68	26.99	23.81	119.06	26.08	2024/5/28	2024/6/21	2024/6/2 9:04	*Streptococcus constellatus*	Death	104879.37	18266.4	10476.94	24
24	1166970	F	51	7.52	6.86	42.8	100	2024/5/31	2024/6/29	2024/6/2 15:01	*Proteus mirabilis*	Recovery	135521.26	26708.8	2427.56	29
25	2476396	F	84	13.65	13	228.01	18.24	2024/6/1	2024/6/10	2024/6/2 14:58	*Escherichia coli*	Recovery	34388.52	7425.5	3033.38	9
26	1598055	M	88	11.01	9.51	145.07	10.88	2024/4/19	2024/7/2	2024/6/6 16:42	*Klebsiella pneumoniae*	Death	298617.62	54463	19732.75	74
27	2319636	F	55	4.44	2.81	NA	0.741	2024/6/3	2024/6/13	2024/6/5 15:17	*Escherichia coli*	Recovery	13553.13	5100.3	645.1	10
28	496832	M	68	6.40	5.06	61.73	0.082	2023/12/14	2024/7/8	2024/6/7 8:47	*Staphylococcus epidermidis*	Death	515172.18	38641.2	3877.81	207
29	2739189	M	75	26.68	25.51	253.72	100	2024/6/5	2024/6/10	2024/6/6 9:16	*Morganella Morganella*	Recovery	25230.19	10303.3	1422.85	5
30	1880629	M	88	12.51	11.24	4.88	0.177	2024/6/5	2024/6/13	2024/6/6 16:41	*Escherichia coli*	Recovery	30935.82	4283.3	2943.01	8
31	2739955	M	84	12.37	11.36	82.98	3.77	2024/6/6	2024/6/11	2024/6/9 9:33	*Cedisicillus Niger*	Recovery	27137.41	3837.5	1038.91	5
32	2736777	M	37	15.56	13.24	NA	0.255	2024/5/30	2024/6/13	2024/6/9 9:11	*Enterococcus*	Recover	44142.28	7524.8	503.22	14

											faecalis	y				
33	2740794	F	75	7.26	6.25	127.6	65.88	2024/6/9	2024/6/28	2024/6/11 15:13	*Klebsiella pneumoniae*	Recovery	93132.92	15688	4022.42	19
34	2068125	M	55	2.63	1.78	NA	0.428	2024/5/20	2024/6/19	2024/6/14 9:09	*Candida parapsilosis*	Death	109567.34	16614.8	6970.15	30
35	2742331	M	82	14.10	13.39	140.27	100	2024/6/12	2024/6/21	2024/6/13 15:01	*Klebsiella pneumoniae*	Recovery	28621.04	6885.3	6488.56	9
36	2739130	F	38	3.26	1.13	NA	0.077	2024/6/4	2024/6/25	2024/6/16 9:09	*Streptococcus pneumoniae*	Recovery	25517.45	12973.1	2717.31	21
37	2701751	M	52	6.50	4.7	NA	0.167	2024/6/13	2024/7/20	2024/6/17 14:43	*Klebsiella pneumoniae*	Recovery	43138.84	5691.3	1109.92	37
39	2704385	F	64	3.67	3.37	8.17	0.268	2024/5/6	2024/6/27	2024/6/20 9:34	*Escherichia coli*	Recovery	59151.52	1931	119.59	52
40	2744294	M	53	10.59	9.8	24.32	1.33	2024/6/18	2024/7/17	2024/6/20 9:05	*Staphylococcus capitis*	Recovery	152764.61	9486.6	2273.81	29
41	2744747	M	46	12.19	10.07	NA	0.07	2024/6/18	2024/7/6	2024/6/24 9:43	*Streptococcus kurtosus*	Death	34119.64	7640.8	4731.46	18
42	1472678	M	61	13.44	11.81	399.02	100	2024/6/19	2024/7/29	2024/6/20 15:07	*Klebsiella pneumoniae*	Recovery	125724.14	15692.1	23587.12	40
43	2720521	F	56	7.59	6.25	207.38	0.191	2024/6/18	2024/7/1	2024/6/21 9:10	*Escherichia coli*	Recovery	16919.81	4578	573.96	13
45	2742261	F	67	13.69	12.76	136.44	46.19	2024/6/12	2024/6/28	2024/6/21 8:56	*Escherichia coli*	Recovery	17149.09	3458.8	736.44	16
46	2571720	F	60	16.47	15.64	43.61	0.047	2024/6/20	2024/6/27	2024/6/24 11:05	*Escherichia coli*	Recovery	31325.75	4529.8	2812.75	7
47	2068125	M	55	2.63	1.69	51.48	0.569	2024/6/19	2024/7/18	2024/6/23 5:52	*Candida parapsilosis*	Death	163764.89	20481.8	18097	29
48	839459	M	63	9.68	9.15	NA	91.67	2024/6/21	2024/6/30	2024/6/22 9:03	*Klebsiella pneumoniae*	Recovery	23676.68	12025.6	3735.82	9
49	2746311	M	58	14.00	11.79	105.55	1.63	2024/6/22	2024/8/24	2024/6/24 9:32	*Staphylococcus aureus*	Recovery	56457.3	12559.7	7097.36	63
50	2746324	M	65	16.11	14.46	128.01	24.81	2024/6/22	2024/7/2	2024/6/24	*Escherichia*	Recover	65788.28	16559.9	1550.87	10

										14:46	*coli*	y				
51	2746104	M	68	3.37	2.28	35	0.662	2024/6/22	2024/7/11	2024/6/24 9:31	*Escherichia coli*	Recovery	46760.97	11292.8	9750.27	19
52	2703582	M	52	0.13	0.06	207.66	18.78	2024/6/23	2024/6/30	2024/6/24 14:49	*Klebsiella pneumoniae*	Death	33390.05	5109.3	188.69	7
53	1101486	F	71	5.96	4.73	3.11	0.046	2024/6/19	2024/7/18	2024/6/27 11:37	*Staphylococcus hominis*	Recovery	113580.4	13802.6	13848.07	29
55	1970178	M	68	0.20	0.03	15.89	0.425	2024/5/28	2024/7/7	2024/6/24 14:51	*Escherichia coli*	Death	59506.7	9380.9	21364.14	40
57	2747567	F	76	11.84	10.4	114	20.69	2024/6/25	2024/7/12	2024/6/26 14:47	*Escherichia coli*	Recovery	39203.51	7629.4	5186.25	17
58	1693385	F	78	20.89	18.66	NA	2.66	2024/6/26	2024/7/25	2024/6/28 14:46	*Klebsiella pneumoniae*	Recovery	115861.67	27932.8	14826.2	29
59	2619593	M	62	4.10	2.9	85.76	0.088	2024/6/11	2024/7/5	2024/6/28 9:26	*Staphylococcus epidermidis* *Staphylococcus aureus* *Staphylococcus capitis*	Recovery	54883.62	3174.5	546.63	24
60	2747539	M	60	10.75	7.27	94.81	0.08	2024/6/25	2024/8/27	2024/6/28 9:07	*Staphylococcus epidermidis* *Staphylococcus hominis*	Recovery	256889.35	35304.6	26089.41	63
61	2739928	M	43	0.01	NA	43.5	0.546	2024/6/6	2024/7/3	2024/6/28 10:00	*Enterococcus faecium* *Staphylococcus epidermidis*	Death	123503.39	19985.2	10388.12	27
62	2748115	M	83	14.62	13.66	113.68	21.45	2024/6/26	2024/7/6	2024/6/28 9:27	*Escherichia coli*	Recovery	31954.75	5307.3	648.06	10
64	2748874	M	87	15.34	14.04	214.23	0.321	2024/6/29	2024/7/6	2024/7/1 8:57	*Streptococcus intermedius*	Recovery	55507.01	5320.3	2982.9	7
65	1960381	F	34	15.00	13.68	10.23	0.064	2024/6/28	2024/7/9	2024/7/1 8:59	*Streptococcus agalactiae*	Recovery	19225.84	4692	818.21	11

											Escherichia coli					
66	2748780	F	71	7.57	5.87	20.29	15.43	2024/6/28	2024/7/6	2024/7/1 14:34	*Klebsiella pneumoniae*	Recovery	20749.86	4082	3443.53	8
67	2749142	F	36	7.96	7.73	96.41	6.21	2024/6/29	2024/7/9	2024/7/1 9:01	*Escherichia coli*	Recovery	21623.49	5849.5	4159.54	10
68	1693385	F	78	12.11	10.97	30.62	0.399	2024/6/26	2024/7/25	2024/7/2 14:55	*Klebsiella pneumoniae*	Recovery	115861.67	27932.8	14826.2	29
71	2752958	M	49	19.76	17.15	135.94	0.174	2024/7/8	2024/7/19	2024/7/9 14:42	*Staphylococcus hominis*	Recovery	22480.98	5075.8	4563.12	11
72	2753019	M	71	18.85	17.77	7.38	10.34	2024/7/8	2024/7/16	2024/7/914:41:37	*Escherichia coli*	Recovery	33347.28	3951.5	1684.07	8
73	2385072	M	85	0.41	0.28	210.77	NA	2024/6/14	2024/7/19	2024/7/15 8:52	*Stenotrophomonas maltophilia*	Recovery	87003.47	14420.4	19483.65	35
75	1064137	M	56	9.15	8.02	23.65	2.28	2024/7/12	2024/7/24	2024/7/14 8:49	*Escherichia coli*	Recovery	14338.64	7712.6	350.68	12
76	2755007	M	51	32.24	30.14	279.34	2.26	2024/7/13	2024/8/11	2024/7/16 9:26	*Proteus mirabilis*	Recovery	75392.33	1244.2	5770.02	29
78	2449336	F	62	12.86	11.51	134.21	0.357	2024/7/1	2024/7/28	2024/7/15 14:59	*Klebsiella pneumoniae*	Death	54261.35	8706.5	1031.86	27
79	2757741	M	62	4.83	4.54	117	7.89	2024/7/19	2024/7/26	2024/7/20 14:24	*Citrobacter freundii*	Recovery	29044.39	4304.3	711.1	7
80	2758078	F	75	40.41	37.83	197.99	40.1	2024/7/21	2024/7/25	2024/7/21 14:45	*Escherichia coli*	Death	141845.62	14847	3221.16	4
81	2756282	F	52	5.88	4.16	NA	0.352	2024/7/16	2024/8/28	2024/7/23 8:55	*Staphylococcus aureus*	Death	160476.87	21014.2	11718.93	43
82	2378914	M	63	11.29	10.71	157.06	4.56	2024/7/21	2024/7/30	2024/7/23 10:37	*Klebsiella pneumoniae*	Death	152486.63	17222.6	9733.54	9
83	2587431	M	57	0.24	0.04	207.8	2.53	2024/7/6	2024/8/9	2024/7/26 9:26	*Enterococcus faecium*	Recovery	127640.75	13368.6	54014.3	34
84	2634722	M	71	18.99	16.41	232.67	0.768	2024/7/21	2024/8/7	2024/7/26 14:14	*Escherichia coli*	Recovery	34557.54	4394.8	5224.48	17

85	2760399	F	80	11.99	11.49	120.59	23.18	2024/7/25	2024/7/31	2024/7/27 9:11	*Escherichia coli*	Death	39342.49	8757	1330.76	6
86	2710535	F	73	24.38	21.79	178.85	74.63	2024/7/27	2024/8/10	2024/7/28 14:22	*Escherichia coli*	Death	61958.67	12313.1	6133.86	14
87	2761135	M	73	13.51	10.89	41.2	0.511	2024/7/27	2024/8/4	2024/7/31 10:06	*Staphylococcus epidermidis*	Recovery	17229.15	5460.3	873.36	8
88	2761975	M	67	6.73	5.89	57.38	1.97	2024/7/29	2024/8/19	2024/7/31 9:10	*Enterococcus faecalis*	Recovery	44993.23	7637.8	5917.23	21
89	2760431	M	49	9.97	8.77	11.34	0.153	2024/7/26	2024/8/11	2024/8/2 9:00	*Staphylococcus hominis*	Recovery	35842.76	5270.8	870.49	16

Table 5 Specimen information of Phase III Pre-op

ID	Admission number	Gender	Age	WBC (10^9/L)	Neutrophil (10^9/L)	CRP (mg/L)	PCT (ng/mL)	Admission date	Date of discharge	Species identification	Organism	Clinical outcomes	Total hospitalization costs	Total laboratory costs	Total antibacterial agents costs	Length of hospital stay
1	2727581	M	81	24.52	22.14	21.22	0.475	2024/5/8	2024/5/11	2024/5/9 14:52	*Escherichia coli*	Death	24389.23	8317.8	165.16	3
2	1515608	M	60	21.65	18.58	NA	1.61	2024/5/7	2024/5/18	2024/5/12 9:01	*Staphylococcus epidermidis*	Death	72370.46	16994.1	2367.25	11
3	2726402	M	53	2.81	2.56	30.63	0.247	2024/5/5	2024/5/20	NA	*Streptococcus angina*	Recovery	22274.23	5277.5	375.6	15
4	2068125	M	55	10.32	8.61	134.62	0.27	2024/3/22	2024/5/20	2024/5/14 9:05	*Enterococcus faecium*	Death	288404.4	31616.4	56208.17	59
5	2729973	F	66	15.55	13.5	NA	0.296	2024/5/13	2024/5/20	2024/5/15 9:06	*Escherichia coli*	Recovery	18261.78	6563.5	3805.09	7
6	2605903	M	54	0.24	0.17	12.47	0.064	2024/5/5	2024/5/24	2024/5/14 14:51	*Escherichia coli*	Recovery	64579.71	8595.1	11264.98	19
7	2114109	M	60	4.90	4.14	16.58	0.114	2024/4/29	2024/5/27	NA	*Enterococcus faecalis*	Recovery	69856.62	8264.3	40056.83	28
8	2731034	M	81	29.44	28.03	243.29	7.04	2024/5/15	2024/5/25	2024/5/16 16:06	*Klebsiella pneumoniae*	Recovery	12037.05	5176	748.5	10
9	2730233	F	69	9.37	8	83.81	0.099	2024/5/14	2024/6/5	2024/5/18 9:36	*Klebsiella pneumoniae*	Recovery	18638.71	5403.8	1123.71	22
10	2098329	F	60	21.67	20.32	324.84	16.22	2024/5/16	2024/6/14	2024/5/18 9:27	*Staphylococcus aureus*	Recovery	69210.74	11743.8	16341.56	29
11	2730092	F	37	12.84	11.51	134.58	0.314	2024/5/13	2024/5/25	2024/5/19 8:42	*Escherichia coli*	Recovery	17367.07	3525.5	376.76	12
12	2732113	F	78	10.02	8.59	252.58	0.337	2024/5/18	2024/6/5	2024/5/21 10:39	*Staphylococcus hominis*	Recovery	39757.14	11004.1	2402.83	18
13	1674759	F	62	2.35	1.83	NA	NA	2024/5/20	2024/5/28	2024/5/24 9:42	*Staphylococcus hominis*	Recovery	40358.16	6494	0	8
14	2733037	F	43	20.25	17.03	223.27	2.01	2024/5/20	2024/6/4	2024/5/24	*Staphylococcu*	Recover	31376.76	7794.8	4251.57	15

										9:12	*s aureus*	y				
15	2726465	M	40	28.69	26.48	152.4	14.59	2024/5/5	2024/6/30	2024/5/23 14:18	*Enterococcus faecium*	Death	632516.18	71808.5	43795.98	56
16	2733717	F	65	11.67	9.65	273.64	2.6	2024/5/22	2024/6/3	2024/5/25 10:36	*Klebsiella pneumoniae*	Recover y	26647.6	5083	2559.53	12
17	677708	F	67	8.73	7.61	161.62	5.75	2024/5/25	2024/5/29	2024/5/27 9:22	*Morganella Morganella*	Recover y	26544.69	3194.8	470.62	4
20	534939	F	89	8.60	7.8	NA	0.819	2024/5/27	2024/6/6	2024/5/29 9:43	*Escherichia coli*	Recover y	22348.99	9381.1	2763.17	10
21	2736301	M	92	8.21	5.8	39.92	0.434	2024/5/28	2024/6/3	2024/5/30 8:23	*Enterococcus casseliflavus*	Recover y	27999.41	4803	906.48	6
22	2737127	M	73	23.30	21.97	59.85	0.251	2024/5/30	2024/7/14	2024/6/1 11:32	*Staphylococcu s aureus*	Recover y	55272.5	9932.2	21945.9	45
23	2735922	M	68	26.99	23.81	119.06	26.08	2024/5/28	2024/6/21	2024/6/2 9:04	*Streptococcus constellatus*	Death	104879.37	18266.4	10476.94	24
24	1166970	F	51	7.52	6.86	42.8	100	2024/5/31	2024/6/29	2024/6/2 15:01	*Proteus mirabilis*	Recover y	135521.26	26708.8	2427.56	29
25	2476396	F	84	13.65	13	228.01	18.24	2024/6/1	2024/6/10	2024/6/2 14:58	*Escherichia coli*	Recover y	34388.52	7425.5	3033.38	9
26	1598055	M	88	11.01	9.51	145.07	10.88	2024/4/19	2024/7/2	2024/6/6 16:42	*Klebsiella pneumoniae*	Death	298617.62	54463	19732.75	74
27	2319636	F	55	4.44	2.81	NA	0.741	2024/6/3	2024/6/13	2024/6/5 15:17	*Escherichia coli*	Recover y	13553.13	5100.3	645.1	10
28	496832	M	68	6.40	5.06	61.73	0.082	2023/12/14	2024/7/8	2024/6/7 8:47	*Staphylococcu s epidermidis*	Death	515172.18	38641.2	3877.81	207
29	2739189	M	75	26.68	25.51	253.72	100	2024/6/5	2024/6/10	2024/6/6 9:16	*Morganella Morganella*	Recover y	25230.19	10303.3	1422.85	5
30	1880629	M	88	12.51	11.24	4.88	0.177	2024/6/5	2024/6/13	2024/6/6 16:41	*Escherichia coli*	Recover y	30935.82	4283.3	2943.01	8
31	2739955	M	84	12.37	11.36	82.98	3.77	2024/6/6	2024/6/11	2024/6/9 9:33	*Cedisicillus Niger*	Recover y	27137.41	3837.5	1038.91	5
32	2736777	M	37	15.56	13.24	NA	0.255	2024/5/30	2024/6/13	2024/6/9 9:11	*Enterococcus*	Recover	44142.28	7524.8	503.22	14

											faecalis	y				
33	2740794	F	75	7.26	6.25	127.6	65.88	2024/6/9	2024/6/28	2024/6/11 15:13	*Klebsiella pneumoniae*	Recovery	93132.92	15688	4022.42	19
34	2068125	M	55	2.63	1.78	NA	0.428	2024/5/20	2024/6/19	2024/6/14 9:09	*Candida parapsilosis*	Death	109567.34	16614.8	6970.15	30
35	2742331	M	82	14.10	13.39	140.27	100	2024/6/12	2024/6/21	2024/6/13 15:01	*Klebsiella pneumoniae*	Recovery	28621.04	6885.3	6488.56	9
36	2739130	F	38	3.26	1.13	NA	0.077	2024/6/4	2024/6/25	2024/6/16 9:09	*Streptococcus pneumoniae*	Recovery	25517.45	12973.1	2717.31	21
37	2701751	M	52	6.50	4.7	NA	0.167	2024/6/13	2024/7/20	2024/6/17 14:43	*Klebsiella pneumoniae*	Recovery	43138.84	5691.3	1109.92	37
39	2704385	F	64	3.67	3.37	8.17	0.268	2024/5/6	2024/6/27	2024/6/20 9:34	*Escherichia coli*	Recovery	59151.52	1931	119.59	52
40	2744294	M	53	10.59	9.8	24.32	1.33	2024/6/18	2024/7/17	2024/6/20 9:05	*Staphylococcus capitis*	Recovery	152764.61	9486.6	2273.81	29
41	2744747	M	46	12.19	10.07	NA	0.07	2024/6/18	2024/7/6	2024/6/24 9:43	*Streptococcus kurtosus*	Death	34119.64	7640.8	4731.46	18
42	1472678	M	61	13.44	11.81	399.02	100	2024/6/19	2024/7/29	2024/6/20 15:07	*Klebsiella pneumoniae*	Recovery	125724.14	15692.1	23587.12	40
43	2720521	F	56	7.59	6.25	207.38	0.191	2024/6/18	2024/7/1	2024/6/21 9:10	*Escherichia coli*	Recovery	16919.81	4578	573.96	13
45	2742261	F	67	13.69	12.76	136.44	46.19	2024/6/12	2024/6/28	2024/6/21 8:56	*Escherichia coli*	Recovery	17149.09	3458.8	736.44	16
46	2571720	F	60	16.47	15.64	43.61	0.047	2024/6/20	2024/6/27	2024/6/24 11:05	*Escherichia coli*	Recovery	31325.75	4529.8	2812.75	7
47	2068125	M	55	2.63	1.69	51.48	0.569	2024/6/19	2024/7/18	2024/6/23 5:52	*Candida parapsilosis*	Death	163764.89	20481.8	18097	29
48	839459	M	63	9.68	9.15	NA	91.67	2024/6/21	2024/6/30	2024/6/22 9:03	*Klebsiella pneumoniae*	Recovery	23676.68	12025.6	3735.82	9
49	2746311	M	58	14.00	11.79	105.55	1.63	2024/6/22	2024/8/24	2024/6/24 9:32	*Staphylococcus aureus*	Recovery	56457.3	12559.7	7097.36	63
50	2746324	M	65	16.11	14.46	128.01	24.81	2024/6/22	2024/7/2	2024/6/24	*Escherichia*	Recover	65788.28	16559.9	1550.87	10

										14:46	*coli*	y				
51	2746104	M	68	3.37	2.28	35	0.662	2024/6/22	2024/7/11	2024/6/24 9:31	*Escherichia coli*	Recovery	46760.97	11292.8	9750.27	19
52	2703582	M	52	0.13	0.06	207.66	18.78	2024/6/23	2024/6/30	2024/6/24 14:49	*Klebsiella pneumoniae*	Death	33390.05	5109.3	188.69	7
53	1101486	F	71	5.96	4.73	3.11	0.046	2024/6/19	2024/7/18	2024/6/27 11:37	*Staphylococcus hominis*	Recovery	113580.4	13802.6	13848.07	29
55	1970178	M	68	0.20	0.03	15.89	0.425	2024/5/28	2024/7/7	2024/6/24 14:51	*Escherichia coli*	Death	59506.7	9380.9	21364.14	40
57	2747567	F	76	11.84	10.4	114	20.69	2024/6/25	2024/7/12	2024/6/26 14:47	*Escherichia coli*	Recovery	39203.51	7629.4	5186.25	17
58	1693385	F	78	20.89	18.66	NA	2.66	2024/6/26	2024/7/25	2024/6/28 14:46	*Klebsiella pneumoniae*	Recovery	115861.67	27932.8	14826.2	29
59	2619593	M	62	4.10	2.9	85.76	0.088	2024/6/11	2024/7/5	2024/6/28 9:26	*Staphylococcus epidermidis* *Staphylococcus aureus* *Staphylococcus capitis*	Recovery	54883.62	3174.5	546.63	24
60	2747539	M	60	10.75	7.27	94.81	0.08	2024/6/25	2024/8/27	2024/6/28 9:07	*Staphylococcus epidermidis* *Staphylococcus hominis*	Recovery	256889.35	35304.6	26089.41	63
61	2739928	M	43	0.01	NA	43.5	0.546	2024/6/6	2024/7/3	2024/6/28 10:00	*Enterococcus faecium* *Staphylococcus epidermidis*	Death	123503.39	19985.2	10388.12	27
62	2748115	M	83	14.62	13.66	113.68	21.45	2024/6/26	2024/7/6	2024/6/28 9:27	*Escherichia coli*	Recovery	31954.75	5307.3	648.06	10
64	2748874	M	87	15.34	14.04	214.23	0.321	2024/6/29	2024/7/6	2024/7/1 8:57	*Streptococcus intermedius*	Recovery	55507.01	5320.3	2982.9	7
65	1960381	F	34	15.00	13.68	10.23	0.064	2024/6/28	2024/7/9	2024/7/1 8:59	*Streptococcus agalactiae*	Recovery	19225.84	4692	818.21	11

											Escherichia coli					
66	2748780	F	71	7.57	5.87	20.29	15.43	2024/6/28	2024/7/6	2024/7/1 14:34	*Klebsiella pneumoniae*	Recovery	20749.86	4082	3443.53	8
67	2749142	F	36	7.96	7.73	96.41	6.21	2024/6/29	2024/7/9	2024/7/1 9:01	*Escherichia coli*	Recovery	21623.49	5849.5	4159.54	10
68	1693385	F	78	12.11	10.97	30.62	0.399	2024/6/26	2024/7/25	2024/7/2 14:55	*Klebsiella pneumoniae*	Recovery	115861.67	27932.8	14826.2	29
71	2752958	M	49	19.76	17.15	135.94	0.174	2024/7/8	2024/7/19	2024/7/9 14:42	*Staphylococcus hominis*	Recovery	22480.98	5075.8	4563.12	11
72	2753019	M	71	18.85	17.77	7.38	10.34	2024/7/8	2024/7/16	2024/7/914:41:37	*Escherichia coli*	Recovery	33347.28	3951.5	1684.07	8
73	2385072	M	85	0.41	0.28	210.77	NA	2024/6/14	2024/7/19	2024/7/15 8:52	*Stenotrophomonas maltophilia*	Recovery	87003.47	14420.4	19483.65	35
75	1064137	M	56	9.15	8.02	23.65	2.28	2024/7/12	2024/7/24	2024/7/14 8:49	*Escherichia coli*	Recovery	14338.64	7712.6	350.68	12
76	2755007	M	51	32.24	30.14	279.34	2.26	2024/7/13	2024/8/11	2024/7/16 9:26	*Proteus mirabilis*	Recovery	75392.33	1244.2	5770.02	29
78	2449336	F	62	12.86	11.51	134.21	0.357	2024/7/1	2024/7/28	2024/7/15 14:59	*Klebsiella pneumoniae*	Death	54261.35	8706.5	1031.86	27
79	2757741	M	62	4.83	4.54	117	7.89	2024/7/19	2024/7/26	2024/7/20 14:24	*Citrobacter freundii*	Recovery	29044.39	4304.3	711.1	7
80	2758078	F	75	40.41	37.83	197.99	40.1	2024/7/21	2024/7/25	2024/7/21 14:45	*Escherichia coli*	Death	141845.62	14847	3221.16	4
81	2756282	F	52	5.88	4.16	NA	0.352	2024/7/16	2024/8/28	2024/7/23 8:55	*Staphylococcus aureus*	Death	160476.87	21014.2	11718.93	43
82	2378914	M	63	11.29	10.71	157.06	4.56	2024/7/21	2024/7/30	2024/7/23 10:37	*Klebsiella pneumoniae*	Death	152486.63	17222.6	9733.54	9
83	2587431	M	57	0.24	0.04	207.8	2.53	2024/7/6	2024/8/9	2024/7/26 9:26	*Enterococcus faecium*	Recovery	127640.75	13368.6	54014.3	34
84	2634722	M	71	18.99	16.41	232.67	0.768	2024/7/21	2024/8/7	2024/7/26 14:14	*Escherichia coli*	Recovery	34557.54	4394.8	5224.48	17

85	2760399	F	80	11.99	11.49	120.59	23.18	2024/7/25	2024/7/31	2024/7/27 9:11	*Escherichia coli*	Death	39342.49	8757	1330.76	6
86	2710535	F	73	24.38	21.79	178.85	74.63	2024/7/27	2024/8/10	2024/7/28 14:22	*Escherichia coli*	Death	61958.67	12313.1	6133.86	14
87	2761135	M	73	13.51	10.89	41.2	0.511	2024/7/27	2024/8/4	2024/7/31 10:06	*Staphylococcus epidermidis*	Recovery	17229.15	5460.3	873.36	8
88	2761975	M	67	6.73	5.89	57.38	1.97	2024/7/29	2024/8/19	2024/7/31 9:10	*Enterococcus faecalis*	Recovery	44993.23	7637.8	5917.23	21
89	2760431	M	49	9.97	8.77	11.34	0.153	2024/7/26	2024/8/11	2024/8/2 9:00	*Staphylococcus hominis*	Recovery	35842.76	5270.8	870.49	16

Table 6 Specimen information of Phase III Post-op

ID	Admission number	Gender	Age	WBC (10^9/L)	Neutrophil (10^9/L)	CRP (μg/mL)	PCT (ng/mL)	Admission date	Date of discharge	Species identification	Organism	Clinical outcomes	Total hospitalization costs	Total laboratory costs	Total antibacterial agents costs	Length of hospital stay
1	2759818	F	58	3.7	3.4	38.04	0.117	2024/7/24	2024/8/9	2024/8/2 9:15	*Klebsiella pneumoniae*	Recovery	65035.89	16770	602.76	16
2	2763190	M	54	11.24	7.78	282.66	0.641	2024/8/1	2024/8/6	2024/8/3 15:00	*Pseudomonas aeruginosa*	Death	70235.98	9710.8	1008.54	5
3	2761991	F	30	7.69	6.87	NA	NA	2024/7/29	2024/8/27	2024/8/8 15:36	*Klebsiella pneumoniae*	Recovery	102065.41	2467.57	1509.62	29
5	2766222	F	22	10.72	9.67	NA	27.33	2024/8/8	2024/9/2	2024/8/12 9:01	*Klebsiella pneumoniae*	Recovery	137060.41	15134.8	794.28	25
6	2763399	M	70	6.84	5.02	37.38	1.04	2024/8/2	2024/10/15	2024/8/15 14:26	*Escherichia coli*	Death	406508.62	55525.6	55612.05	74
7	2771837	F	70	17.21	15.42	107.15	0.164	2024/8/22	2024/9/21	2024/8/24 9:01	*Staphylococcus aureus*	Recovery	84257.59	15469.3	8512.68	30
8	2777258	F	31	16.56	15.75	71.01	11.22	2024/9/5	2024/9/15	2024/9/10 8:57	*Klebsiella mutabilis*	Recovery	22071.07	5441.5	341.2	10
9	2758590	M	49	6.23	4.5	160.94	0.167	2024/9/1	2024/9/18	2024/9/15 14:30	*Enterococcus faecium*	Death	105874.53	23753	3257.84	17
10	2763220	F	91	10.42	9.44	143.68	100	2024/8/1	2024/10/15	2024/9/16 13:50	*Acinetobacter baumannii*	Recovery	214485.93	35272	17366.67	75
11	2778164	F	98	8.45	5.98	NA	0.146	2024/9/8	2024/9/26	2024/9/17 15:09	*Acinetobacter baumannii*	Death	128842.15	15072	28712.76	18
15	2264591	F	59	20.19	19.26	13.37	12.93	2024/8/26	2024/8/31	2024/8/29 9:01	*Escherichia coli*	Recovery	25222.99	4613.3	514.07	5
16	2778701	F	73	5.41	4.9	41.69	0.173	2024/9/9	2024/9/23	2024/9/14 10:13	*Escherichia coli*	Recovery	18527.78	6505.8	714.26	14
18	2767941	M	55	4.11	3.15	31.93	0.19	2024/8/12	2024/8/27	2024/8/15 8:58	*Staphylococcus hominis*	Recovery	29064.97	6416.8	588.42	15
19	1355432	F	77	11.61	10.19	10.09	0.29	2024/8/3	2024/8/6	2024/8/4	*Escherichia*	Recover	24215.62	3503	304.23	3

										14:48	*coli*	y				
20	611745	F	68	24.86	21.83	335.85	1.07	2024/8/3	2024/8/11	2024/8/6 10:36	*Klebsiella pneumoniae*	Recovery	10660.96	3757.5	800.67	8
22	777143	M	60	11.83	10.73	37.55	0.209	2024/8/12	2024/8/20	2024/8/14 14:52	*Escherichia coli*	Recovery	17896.22	5150.5	618.64	8
23	2703425	M	62	17.15	15.85	NA	25.38	2024/8/12	2024/8/31	2024/8/14 8:24	*Escherichia coli*、*Klebsiella pneumoniae*	Recovery	56885.83	8993.5	9089.76	19
24	704880	F	75	9.41	7.57	NA	0.308	2024/8/22	2024/9/10	2024/8/29 9:15	*Staphylococcus hominis*	Recovery	38516.81	4475.1	1363.66	19
26	2769623	M	52	9.98	9.27	273.7	30.06	2024/8/16	2024/9/2	2024/8/19 9:10	*Staphylococcus aureus*	Death	113228.86	12448.7	13530.06	17
27	2782322	F	69	5.08	3.92	NA	1.05	2024/9/20	2024/10/1	2024/9/27 14:39	*Enterococcus faecium*	Recovery	49224.91	8517.8	6785.9	11
28	1392545	F	58	10.04	8.25	207.66	88.86	2024/8/14	2024/8/21	2024/8/16 15:11	*Staphylococcus hominis*	Recovery	20936.84	4091.8	823.45	7
29	1090243	M	50	16.9	16.33	52.93	3.31	2024/9/10	2024/9/25	2024/9/17 15:08	*Klebsiella pneumoniae*	Death	98217.74	8734.8	18810.66	15
30	2619921	M	71	22.07	20.79	259.09	100	2024/10/11	2024/10/20	2024/10/13 9:11	*Escherichia coli*	Recovery	25080.26	4604.5	2019.36	9
31	2648439	M	61	3.72	2.17	92.9	5.4	2024/8/28	2024/9/3	2024/8/29 15:02	*Escherichia coli*	Recovery	31073.82	3782.8	4260.98	6
32	2764553	M	74	8.79	7.28	53.69	0.12	2024/8/5	2024/8/29	2024/8/8 8:48	*Staphylococcus hominis*	Recovery	29911.33	8855.1	7020.59	24
33	778829	M	55	11.84	10.79	NA	0.502	2024/10/4	2024/10/23	2024/10/6 14:11	*Klebsiella pneumoniae*	Recovery	63504.57	8175.4	14066.56	19
34	2534097	M	66	0.17	0.03	278	2.61	2024/8/9	2024/8/18	2024/8/10 14:52	*Klebsiella pneumoniae*	Death	29749	4749.2	11458.71	9
35	2781017	M	68	104.22	56.91	39.41	4.69	2024/9/17	2024/10/16	2024/9/19 14:48	*Pseudomonas aeruginosa*	Recovery	72693.62	14395.9	18904.77	29
36	2775571	M	71	7.3	6.11	310.97	26.9	2024/9/1	2024/9/10	2024/9/3 8:56	*Klebsiella pneumoniae*	Recovery	20278.06	7864.3	5733.4	9

37	2786752	M	52	0.16	0	120.3	0.802	2024/10/4	2024/10/16	2024/10/5 14:13	*Escherichia coli*	Recovery	46715.15	10972.8	15575.12	12
38	2528602	F	67	7.51	5.61	217.2	24.21	2024/10/8	2024/10/16	2024/10/9 13:51	*Escherichia coli*	Recovery	11862.09	3326.1	5253.6	8
39	2526435	F	77	7.46	3.05	59.4	0.494	2024/10/6	2024/11/4	2024/10/8 9:10	*Staphylococcus aureus*	Recovery	62111.74	8209.6	19091.74	29
40	2327002	F	54	2.9	1.98	6.17	0.175	2024/9/27	2024/10/18	2024/9/30 14:37	*Escherichia coli*	Recovery	32505	6733.8	12031.63	21
43	2767920	M	55	10.55	9	10.72	0.131	2024/8/12	2024/8/19	2024/8/14 10:01	*Escherichia coli*	Recovery	25708.38	4120	628.42	7
44	2769461	M	71	8.43	7.12	22.84	0.574	2024/8/16	2024/8/27	2024/8/17 14:44	*Klebsiella oxytoca*	Recovery	28635.68	4910.3	559.37	11
45	1046170	M	51	11.27	9.44	264.39	1.12	2024/9/5	2024/10/22	2024/9/6 14:56	*Escherichia coli*	Recovery	115023.5	3029.5	698.37	47
46	2166825	M	59	4.06	3.57	16.42	0.108	2024/8/11	2024/8/22	2024/8/16 8:46	*Staphylococcus hominis*	Recovery	9939.41	2808.3	696.48	11
47	2726904	M	64	0.11	0.05	59.87	0.529	2024/7/16	2024/8/20	2024/8/5 15:31	*Enterobacter cloacae*	Recovery	91859.22	4012.5	3103.68	35
48	2767854	F	40	6.59	5.54	203.47	0.094	2024/8/12	2024/9/1	2024/8/27 9:01	*Staphylococcus aureus*	Recovery	40823.56	5947.3	213.1	20
49	2778178	M	77	8.47	7.29	50.29	0.376	2024/9/25	2024/10/24	2024/10/10 9:49	*Klebsiella pneumoniae*	Recovery	57812.53	12658.9	14425.84	29
50	2772022	F	70	16.84	15.74	115.16	0.487	2024/8/23	2024/9/14	2024/9/8 15:06	*Escherichia coli*	Recovery	43367.04	5171.3	365.13	22
51	730119	F	60	3.61	3.29	NA	2.28	2024/9/15	2024/9/26	2024/9/17 15:21	*Escherichia coli*	Recovery	52050.35	5460	5040.85	11
55	2701751	M	52	2.43	1.64	10.92	0.237	2024/9/12	2024/9/27	2024/9/14 14:42	*Escherichia coli*	Recovery	12381.51	3408.1	914.08	15
56	590168	M	55	9.45	8.49	256.09	1.43	2024/9/12	2024/9/21	2024/9/14 14:44	*Klebsiella pneumoniae*	Recovery	13033.2	3444.8	413.09	9
57	2783509	F	57	2.17	1.42	NA	25.66	2024/9/24	2024/10/7	2024/9/25 14:33	*Klebsiella pneumoniae*	Recovery	19852.83	4165	1474.54	13

58	2786629	M	62	14.33	12.99	90.3	4.6	2024/10/4	2024/10/12	2024/10/7 14:02	*Klebsiella pneumoniae*	Recovery	14745.72	3700.3	296.29	8
59	2760611	M	89	16.43	16.02	269.27	65.57	2024/8/5	2024/8/11	2024/8/8 14:43	*Escherichia coli*	Recovery	8721.41	4008	342.24	6
60	2764260	M	61	9.1	8.21	11	1.53	2024/8/4	2024/8/12	2024/8/7 9:31	*Staphylococcus hominis*，*Staphylococcus epidermidis*	Recovery	15559.74	3598.3	2290.74	8
61	906072	M	48	17.09	16.4	NA	NA	2024/9/21	2024/9/27	2024/9/22 14:56	*Klebsiella pneumoniae*	Recovery	16625.84	3351.3	451.28	6
62	2167849	M	58	14.43	13.51	NA	NA	2024/8/25	2024/9/5	2024/8/27 8:53	*Enterococcus faecium*	Recovery	32403.49	3853.5	471.76	11
63	2767039	F	37	0.23	0.17	34.8	0.659	2024/9/1	2024/9/27	2024/9/17 15:08	*Klebsiella pneumoniae*	Recovery	185863.03	10896.5	11769.29	26
64	2376632	F	62	0.03	NA	18.55	0.084	2024/9/23	2024/10/22	2024/10/7 14:08	*Klebsiella pneumoniae*	Recovery	98509.09	7550.8	14618.76	29
65	2752918	F	63	9.45	7.54	79.72	9.04	2024/7/8	2024/8/23	2024/8/3 10:04	*Staphylococcus epidermidis*	Recovery	127727.93	15196.5	18046.95	46
66	864272	M	77	4.04	3.31	8.01	0.054	2024/8/2	2024/8/13	2024/8/5 8:57	*Staphylococcus hominis*	Recovery	11386.14	3169.5	509.2	11
67	2761707	M	78	6.93	6.2	25.97	0.463	2024/7/29	2024/8/11	2024/8/5 8:43	*Enterobacter asheri*	Recovery	23716.64	4895	170.16	13
68	2783778	F	59	6.62	5.46	88.95	0.521	2024/9/25	2024/10/9	2024/9/30 14:37	*Escherichia coli*	Recovery	18353.23	7961.8	1531.1	14
69	2764085	M	78	9.43	8.62	38.06	0.33	2024/8/3	2024/8/9	2024/8/5 8:59	*Staphylococcus epidermidis*	Recovery	8474.88	4572.3	533.01	6
70	2765827	M	70	8.73	7	64.44	0.181	2024/8/7	2024/8/14	2024/8/9 15:06	*Staphylococcus hominis*	Recovery	10612.41	4382.3	501.5	7
71	1493671	F	88	10.41	9.45	125.04	3.05	2024/8/31	2024/9/11	2024/9/1 14:46	*Escherichia coli*	Recovery	12865.07	4492.5	569.48	11
72	2736203	M	65	1.41	1.23	275.09	3.21	2024/9/9	2024/9/13	2024/9/10 15:18	*Klebsiella pneumoniae*	Death	23717.8	4342.5	438.2	4
75	2606574	M	60	4.96	4.29	113.12	4.33	2024/9/6	2024/9/20	2024/9/16	*Escherichia*	Recover	31022.8	3058.3	194.36	14

										13:52	*coli*	y				
76	1862461	M	60	23.13	21.95	107.36	1.35	2024/8/19	2024/8/28	2024/8/20 15:23	*Escherichia coli*	Recovery	20349.71	3752.6	365.24	9
77	2713090	M	71	9.02	8.08	122.19	0.595	2024/8/6	2024/8/20	2024/8/12 14:46	*Acinetobacter Pitti*	Recovery	26979.61	4124.3	658.11	14
78	2738031	F	68	4.85	3.33	220.67	0.302	2024/9/15	2024/9/27	2024/9/17 15:08	*Escherichia coli*	Recovery	27480.08	3413.8	427.96	12
79	1665049	F	31	9.98	7.68	40.65	0.049	2024/8/19	2024/8/30	2024/8/22 15:45	*Staphylococcus hominis*	Recovery	8261.58	3373	483.02	11
80	1148238	M	63	7.65	6.45	8.59	0.096	2024/10/8	2024/10/16	2024/10/12 8:14	*Escherichia coli*	Recovery	10421.02	2448.8	411.7	8
81	2488372	M	47	8.91	8.38	305.4	4.47	2024/9/19	2024/9/27	2024/9/20 14:29	*Escherichia coli*	Recovery	8543.75	2943.3	3177.12	8
82	2785350	M	69	14.97	12.27	185.22	0.371	2024/9/29	2024/10/7	2024/10/1 15:04	*Escherichia coli*	Recovery	15502.23	4919.3	1181.1	8
83	563229	M	84	8.88	7.95	NA	4.66	2024/8/19	2024/10/3	2024/9/17 9:13	*Staphylococcus haemolyticus*	Death	292825.16	36135.2	28883.64	45
84	2017592	M	74	17.47	16.16	163.34	1.13	2024/8/15	2024/8/22	2024/8/17 14:12	*Enterobacter cloacae*	Recovery	20573.88	4422	3581.91	7
85	2785890	M	81	12.37	11.59	202.85	49.39	2024/10/1	2024/10/10	2024/10/2 14:49	*Escherichia coli*	Recovery	15638.18	5072.3	780	9
86	2786019	M	65	8.52	7.52	NA	1.03	2024/10/1	2024/10/16	2024/10/12 14:10	*Enterococcus faecium*	Recovery	31631.41	6701.5	3044.04	15
87	2782142	F	61	12.87	12.07	174.92	100	2024/9/20	2024/9/27	2024/9/21 14:32	*Escherichia coli*	Recovery	26256.86	8336	2636.62	7
88	2598710	F	60	8.14	7.43	30.32	0.542	2024/9/10	2024/9/24	2024/9/12 14:55	*Escherichia coli*	Recovery	9692.54	3613.6	588.06	14
89	2764105	M	60	22.73	21.77	267.56	94.53	2024/8/3	2024/8/20	2024/8/4 14:48	*Klebsiella pneumoniae*	Death	33798.05	5166.6	3852.36	17

NA = not applicable
