# Supplementary material for: Multifactorial impacts of blood culture process optimization on clinical outcomes and healthcare economics in bloodstream infection management
Source: Front Cell Infect Microbiol. 2026 Mar 13;16:1699905. doi: 10.3389/fcimb.2026.1699905 (PMC13021603; doi:10.3389/fcimb.2026.1699905)
Supplement: Supplementary file 2 [file DataSheet2.pdf]

Table 1 Laboratory endpoints of Phase I Pre-op

ID	Admission number	Blood culture collection	Blood culture loading time	Gram staining report	Final AST report time	TAT for Gram-staining reports of positive cultures	Time to microbial species identification for positive cultures	Total TAT from specimen collection to clinician receipt of reports
1	2574911	2023/6/11 0:41	2023/6/11 8:11	2023/6/14 9:15	2023/6/15 15:43	80.6	111.0	111.0
2	423856	2023/8/20 11:02	2023/8/20 11:12	2023/8/23 16:12	2023/8/25 11:29	77.2	120.5	120.5
3	2572959	2023/6/9 22:04	2023/6/10 8:06	2023/6/10 17:34	2023/6/12 14:35	19.5	64.5	64.5
4	2579313	2023/7/4 15:18	2023/7/4 16:06	2023/7/5 9:29	2023/7/6 11:58	18.2	44.7	44.7
5	2583086	2023/6/27 22:54	2023/6/28 8:20	2023/6/30 16:47	2023/7/1 11:27	65.9	84.5	84.5
6	2585263	2023/7/9 23:56	2023/7/10 8:13	2023/7/12 15:29	2023/7/14 16:23	63.6	112.5	112.5
7	848591	2023/8/11 16:43	2023/8/11 17:28	2023/8/12 9:27	2023/8/14 14:20	16.7	69.6	69.6
8	2486645	2023/6/16 11:51	2023/6/16 15:58	2023/6/18 15:19	2023/6/19 11:31	51.5	71.7	71.7
9	1638711	2023/8/14 16:31	2023/8/14 16:59	2023/8/15 8:56	2023/8/17 11:11	16.4	66.7	66.7
10	1061373	2023/7/24 19:50	2023/7/25 7:52	2023/7/25 16:28	2023/7/27 11:11	20.6	63.3	63.3
11	425915	2023/7/28 23:22	2023/7/29 9:20	2023/7/30 10:17	2023/8/1 11:28	34.9	84.1	84.1
12	429943	2023/6/8 18:19	2023/6/9 7:58	2023/6/9 16:53	2023/6/11 10:03	22.6	63.7	63.7
13	2608003	2023/8/15 15:36	2023/8/15 16:49	2023/8/17 9:36	2023/8/19 10:49	42.0	91.2	91.2
14	2587009	2023/7/15 9:39	2023/7/15 10:54	2023/7/21 8:37	2023/7/21 11:14	143.0	145.6	145.6
15	2068125	2023/8/15 7:52	2023/8/15 9:50	2023/8/16 16:15	2023/8/18 8:51	32.4	73.0	73.0
16	2584548	2023/7/1 2:20	2023/7/1 8:11	2023/7/2 16:22	2023/7/4 16:18	38.0	86.0	86.0
17	2600529	2023/8/7 23:36	2023/8/8 8:53	2023/8/10 15:16	2023/8/12 11:27	63.7	107.9	107.9
18	2572281	2023/6/28 11:19	2023/6/28 11:37	2023/6/29 8:55	2023/7/1 11:41	21.6	72.4	72.4
19	583280	2023/8/3 19:00	2023/8/4 9:13	2023/8/4 16:33	2023/8/6 11:56	21.6	64.9	64.9
20	662115	2023/8/9 17:31	2023/8/10 9:00	2023/8/11 16:17	2023/8/13 10:52	46.8	89.3	89.3
21	1119804	2023/8/25 15:27	2023/8/25 16:15	2023/8/26 17:49	2023/8/28 10:06	26.4	66.6	66.6
22	1936185	2023/7/24 9:24	2023/7/24 11:01	2023/7/25 12:30	2023/7/27 11:03	27.1	73.6	73.6
23	2156877	2023/7/16 13:41	2023/7/16 16:19	2023/7/17 15:31	2023/7/19 15:42	25.8	74.0	74.0
24	1463379	2023/6/3 7:53	2023/6/3 9:20	2023/6/5 9:26	2023/6/6 14:49	49.5	78.9	78.9
25	1344868	2023/7/27 21:34	2023/7/28 8:17	2023/7/29 15:52	2023/7/31 14:53	42.3	89.3	89.3
26	2585235	2023/7/9 10:22	2023/7/9 10:32	2023/7/10 14:36	2023/7/11 10:25	28.2	48.1	48.1
27	2583580	2023/6/27 7:27	2023/6/29 8:59	2023/6/30 9:25	2023/7/1 11:38	74.0	100.2	100.2
28	2578282	2023/6/17 12:41	2023/6/17 14:14	2023/6/19 9:29	2023/6/21 15:10	44.8	98.5	98.5
29	2346870	2023/6/21 22:50	2023/6/22 8:19	2023/6/24 9:41	2023/6/25 11:17	58.8	84.4	84.4
30	472318	2023/6/7 14:32	2023/6/7 16:26	2023/6/9 8:08	2023/6/10 3:42	41.6	61.2	61.2
31	2608109	2023/8/14 19:42	2023/8/15 8:11	2023/8/16 9:19	2023/8/18 14:58	37.6	91.3	91.3
32	2194718	2023/7/7 17:14	2023/7/7 17:27	2023/7/9 9:41	2023/7/11 10:41	40.5	89.5	89.5
33	2591402	2023/7/12 4:44	2023/7/12 8:07	2023/7/13 14:46	2023/7/14 11:19	34.0	54.6	54.6
34	951693	2023/7/7 3:12	2023/7/7 8:03	2023/7/8 9:53	2023/7/10 14:49	30.7	83.6	83.6
35	2591726	2023/7/13 23:52	2023/7/14 8:59	2023/7/16 9:08	2023/7/18 11:00	57.3	107.1	107.1
36	2542320	2023/8/4 18:22	2023/8/5 8:04	2023/8/7 8:55	2023/8/7 14:28	62.6	68.1	68.1
37	2474821	2023/8/17 23:54	2023/8/18 7:58	2023/8/20 15:38	2023/8/22 11:26	63.7	107.5	107.5
38	2298599	2023/6/3 11:30	2023/6/3 11:42	2023/6/4 10:04	2023/6/5 14:45	22.6	51.3	51.3
39	713080	2023/8/22 0:01	2023/8/22 8:14	2023/8/23 9:44	2023/8/24 14:49	33.7	62.8	62.8

40	2569136	2023/6/6 15:50	2023/6/7 8:55	2023/6/8 9:08	2023/6/10 8:47	41.3	89.0	89.0
41	2249108	2023/8/2 14:23	2023/8/2 16:15	2023/8/3 9:17	2023/8/4 15:29	18.9	49.1	49.1
42	2605502	2023/8/9 7:40	2023/8/9 8:49	2023/8/10 11:14	2023/8/12 11:34	27.6	75.9	75.9
43	1751286	2023/7/23 5:54	2023/7/23 8:15	2023/7/25 9:16	2023/7/27 10:56	51.4	101.0	101.0
44	2595657	2023/7/20 1:16	2023/7/20 8:14	2023/7/21 10:01	2023/7/23 14:44	32.8	85.5	85.5
45	2615009	2023/8/28 1:00	2023/8/28 8:21	2023/8/29 9:12	2023/8/30 16:15	32.2	63.3	63.3
46	2610657	2023/8/18 0:02	2023/8/18 8:01	2023/8/19 9:09	2023/8/21 10:18	33.1	82.3	82.3
47	2232830	2023/8/5 6:32	2023/8/5 8:04	2023/8/6 16:04	2023/8/8 15:46	33.5	81.2	81.2
48	2531913	2023/8/26 13:58	2023/8/26 16:23	2023/8/28 9:40	2023/8/29 11:15	43.7	69.3	69.3
49	2585279	2023/7/5 5:49	2023/7/5 8:05	2023/7/6 16:46	2023/7/8 11:13	35.0	77.4	77.4
50	2282373	2023/6/10 7:19	2023/6/10 9:30	2023/6/11 9:10	2023/6/12 14:16	25.9	55.0	55.0
51	2586841	2023/7/5 11:33	2023/7/5 14:15	2023/7/7 8:57	2023/7/7 10:58	45.4	47.4	47.4
52	2555325	2023/6/9 5:52	2023/6/9 8:01	2023/6/10 9:04	2023/6/12 14:15	27.2	80.4	80.4
53	2206763	2023/7/12 15:03	2023/7/12 16:10	2023/7/13 14:46	2023/7/14 11:17	23.7	44.2	44.2
54	1430146	2023/6/28 23:35	2023/6/29 9:00	2023/6/29 16:56	2023/7/1 11:18	17.3	59.7	59.7
55	2587007	2023/7/9 15:27	2023/7/9 16:11	2023/7/10 10:14	2023/7/11 10:25	18.8	43.0	43.0
56	2603450	2023/8/4 11:07	2023/8/4 16:27	2023/8/6 11:18	2023/8/8 15:46	48.2	100.7	100.7
57	1156225	2023/6/26 21:09	2023/6/27 8:11	2023/7/1 9:51	2023/7/2 15:06	108.7	138.0	138.0
58	2585739	2023/8/10 10:43	2023/8/10 16:09	2023/8/11 9:23	2023/8/12 11:47	22.7	49.1	49.1
59	1854120	2023/7/22 10:08	2023/7/22 11:06	2023/7/23 10:16	2023/7/24 14:35	24.1	52.5	52.5
60	2606283	2023/8/10 16:06	2023/8/11 8:18	2023/8/12 9:50	2023/8/14 14:31	41.7	94.4	94.4
61	2019574	2023/6/27 9:07	2023/6/27 10:59	2023/6/28 9:51	2023/6/29 11:16	24.7	50.2	50.2
62	2505503	2023/7/18 11:28	2023/7/18 11:53	2023/7/19 8:43	2023/7/20 10:58	21.2	47.5	47.5
63	2612806	2023/8/22 11:54	2023/8/22 16:06	2023/8/23 17:31	2023/8/25 13:23	29.6	73.5	73.5
64	2587660	2023/7/7 9:05	2023/7/7 10:52	2023/7/9 9:23	2023/7/10 14:59	48.3	77.9	77.9
65	2602235	2023/8/21 7:12	2023/8/21 8:49	2023/8/23 14:48	2023/8/25 15:02	55.6	103.8	103.8
66	1140735	2023/7/16 16:38	2023/7/17 8:06	2023/7/18 9:25	2023/7/19 15:08	40.8	70.5	70.5
67	2600482	2023/7/30 1:32	2023/7/30 8:12	2023/7/31 14:11	2023/8/1 14:28	36.7	60.9	60.9
68	2611726	2023/8/26 9:30	2023/8/26 11:10	2023/8/27 9:13	2023/8/28 9:50	23.7	48.3	48.3
69	778031	2023/7/8 2:13	2023/7/8 8:06	2023/7/9 9:28	2023/7/11 10:17	31.2	80.1	80.1
70	2598061	2023/7/24 18:12	2023/7/25 7:55	2023/7/27 8:54	2023/7/27 9:07	62.7	62.9	62.9
71	2432655	2023/8/5 17:37	2023/8/6 9:13	2023/8/7 9:31	2023/8/8 15:41	39.9	70.1	70.1
72	2610906	2023/8/18 11:18	2023/8/18 12:16	2023/8/20 9:36	2023/8/21 10:49	46.3	71.5	71.5
73	2453744	2023/7/23 12:28	2023/7/23 16:02	2023/7/24 9:28	2023/7/26 10:36	21.0	70.1	70.1
74	2616201	2023/8/31 14:54	2023/8/31 16:04	2023/9/1 14:55	2023/9/4 11:18	24.0	92.4	92.4
75	2603193	2023/8/3 23:56	2023/8/4 9:11	2023/8/4 16:36	2023/8/6 11:59	16.7	60.0	60.0
76	2613342	2023/8/23 15:40	2023/8/23 16:32	2023/8/24 10:13	2023/8/25 13:33	18.5	45.9	45.9
77	2230416	2023/8/28 5:56	2023/8/28 8:10	2023/8/29 9:37	2023/8/31 14:43	27.7	80.8	80.8
78	333170	2023/7/7 17:12	2023/7/8 9:46	2023/7/9 9:32	2023/7/11 10:41	40.3	89.5	89.5
79	1673013	2023/7/6 23:06	2023/7/7 8:54	2023/7/7 16:36	2023/7/9 10:49	17.5	59.7	59.7
80	2612836	2023/8/29 12:57	2023/8/29 16:12	2023/8/30 16:57	2023/9/1 10:51	28.0	69.9	69.9
81	2436897	2023/8/4 12:41	2023/8/4 16:27	2023/8/6 10:22	2023/8/9 11:23	45.7	118.7	118.7
82	945990	2023/8/11 13:58	2023/8/11 16:20	2023/8/12 9:33	2023/8/14 14:36	19.6	72.6	72.6
83	2614276	2023/8/25 21:02	2023/8/26 8:30	2023/8/27 15:03	2023/8/28 9:53	42.0	60.9	60.9
84	904962	2023/6/20 10:40	2023/6/20 14:18	2023/6/21 15:37	2023/6/23 14:17	28.9	75.6	75.6
85	2587470	2023/7/6 22:40	2023/7/7 8:58	2023/7/9 8:29	2023/7/9 10:51	57.8	60.2	60.2
86	2583117	2023/6/28 6:13	2023/6/28 8:20	2023/6/30 9:54	2023/7/2 15:00	51.7	104.8	104.8

87	2587936	2023/7/14 6:16	2023/7/14 8:14	2023/7/15 9:01	2023/7/16 11:33	26.8	53.3	53.3
88	2168475	2023/6/28 6:38	2023/6/28 8:16	2023/6/29 9:16	2023/6/30 10:22	26.6	51.7	51.7
89	2600478	2023/7/30 0:38	2023/7/30 8:19	2023/7/31 16:43	2023/8/2 9:50	40.1	81.2	81.2
90	2615964	2023/8/30 8:52	2023/8/30 11:18	2023/9/1 9:14	2023/9/2 10:27	48.4	73.6	73.6
91	2575080	2023/6/10 12:20	2023/6/10 16:20	2023/6/13 10:51	2023/6/15 15:47	70.5	123.4	123.4
92	1256506	2023/8/12 15:27	2023/8/12 16:22	2023/8/13 10:08	2023/8/14 14:23	18.7	46.9	46.9
93	2571625	2023/6/2 16:27	2023/6/2 16:47	2023/6/4 10:08	2023/6/6 14:44	41.7	94.3	94.3
94	2600529	2023/8/7 15:01	2023/8/7 16:14	2023/8/8 8:59	2023/8/9 10:57	18.0	43.9	43.9
95	2599494	2023/7/27 14:04	2023/7/27 16:08	2023/7/28 9:41	2023/7/30 10:43	19.6	68.7	68.7
96	835333	2023/6/30 16:29	2023/6/30 17:09	2023/7/1 9:35	2023/7/2 15:04	17.1	46.6	46.6
97	761061	2023/8/7 20:46	2023/8/8 8:02	2023/8/9 9:03	2023/8/10 14:41	36.3	65.9	65.9
98	2577499	2023/6/26 10:09	2023/6/26 11:01	2023/6/27 9:57	2023/6/29 11:14	23.8	73.1	73.1
99	601261	2023/7/24 17:46	2023/7/25 8:56	2023/7/25 15:39	2023/7/27 11:06	21.9	65.3	65.3
100	2585522	2023/7/3 11:18	2023/7/3 16:33	2023/7/4 9:24	2023/7/5 12:15	22.1	48.9	48.9

Table 2 Laboratory endpoints of Phase I Post-op

ID	Admission number	Blood culture collection	Blood culture loading time	Gram staining report	Species identification	Final AST report time	TAT for Gram-staining reports of positive cultures	Time to microbial species identification for positive cultures	Total TAT from specimen collection to clinician receipt of reports
1	2133796	2023/12/25 15:51	2023/12/25 16:40	2023/12/26 9:46	2023/12/26 14:28	2023/12/27 9:53	17.9	22.6	42.0
2	2629821	2023/10/27 9:04	2023/10/27 11:14	2023/10/28 14:15	2023/10/29 9:45	2023/10/29 11:35	29.2	48.7	50.5
3	2643805	2023/11/4 8:45	2023/11/4 10:07	2023/11/5 9:22	2023/11/5 15:04	2023/11/6 14:19	24.6	30.3	53.6
4	2648162	2023/11/13 18:10	2023/11/14 8:04	2023/11/15 10:14	2023/11/16 9:30	2023/11/16 10:29	40.1	63.3	64.3
5	2626285	2023/10/13 23:00	2023/10/14 8:12	2023/10/15 15:09	2023/10/16 9:56	2023/10/17 11:28	40.2	58.9	84.5
6	2629935	2023/10/5 17:10	2023/10/6 7:56	2023/10/6 15:36	2023/10/7 9:25	2023/10/8 11:11	22.4	40.3	66.0
7	2659898	2023/12/10 1:54	2023/12/10 7:57	2023/12/11 9:27	2023/12/11 14:46	2023/12/12 14:43	31.6	36.9	60.8
8	2321424	2023/10/31 10:58	2023/10/31 11:06	2023/11/1 8:24	2023/11/3 13:42	2023/11/3 13:45	21.4	74.7	74.8
9	2650342	2023/11/18 18:57	2023/11/19 7:58	2023/11/19 16:02	2023/11/20 9:12	2023/11/21 14:44	21.1	38.3	67.8
10	885820	2023/10/11 7:46	2023/10/11 8:52	2023/10/12 15:00	2023/10/13 9:05	2023/10/13 14:39	31.2	49.3	54.9
11	1147461	2023/12/19 20:57	2023/12/20 8:34	2023/12/21 16:25	2023/12/22 9:00	2023/12/24 10:17	43.5	60.1	109.3
12	472318	2023/10/5 6:03	2023/10/5 8:10	2023/10/6 9:29	2023/10/7 8:49	2023/10/8 10:36	27.4	50.8	76.6
13	1294946	2023/11/27 14:56	2023/11/27 15:53	2023/11/28 8:55	2023/11/28 14:21	2023/11/30 14:38	18.0	23.4	71.7
14	2591640	2023/12/12 16:32	2023/12/12 16:43	2023/12/14 9:37	2023/12/15 8:57	2023/12/16 11:12	41.1	64.4	90.7
15	2111820	2023/10/4 11:07	2023/10/4 16:06	2023/10/5 15:03	2023/10/6 9:21	2023/10/7 10:05	27.9	46.2	71.0
16	2655692	2023/12/20 19:25	2023/12/21 8:12	2023/12/22 9:45	2023/12/22 14:55	2023/12/23 11:46	38.3	43.5	64.4
17	400634	2023/11/2 3:43	2023/11/2 8:05	2023/11/4 11:27	2023/11/5 8:47	2023/11/6 14:16	55.7	77.1	106.6
18	2200718	2023/11/16 15:07	2023/11/16 16:38	2023/11/17 16:24	2023/11/18 9:35	2023/11/19 11:37	25.3	42.5	68.5
19	1664630	2023/12/20 6:37	2023/12/20 9:47	2023/12/23 9:35	2023/12/23 14:28	2023/12/24 10:30	75.0	79.9	99.9
20	2631981	2023/10/9 13:24	2023/10/9 16:04	2023/10/10 10:56	2023/10/11 9:09	2023/10/12 10:29	21.5	43.8	69.1
21	2360965	2023/12/2 7:20	2023/12/2 9:05	2023/12/3 8:53	2023/12/4 9:02	2023/12/5 11:30	25.6	49.7	76.2
22	1856791	2023/11/28 14:46	2023/11/28 15:48	2023/11/29 14:57	2023/11/30 8:43	2023/12/1 13:51	24.2	42.0	71.1
23	2629312	2023/10/3 16:07	2023/10/4 8:06	2023/10/4 16:37	2023/10/5 9:33	2023/10/6 11:10	24.5	41.4	67.1
24	2665360	2023/12/20 18:02	2023/12/21 8:52	2023/12/22 9:46	2023/12/22 14:59	2023/12/23 11:48	39.7	45.0	65.8
25	2632474	2023/10/10 12:09	2023/10/10 16:01	2023/10/11 8:53	2023/10/11 16:11	2023/10/12 10:36	20.7	28.0	46.5
26	2636564	2023/10/19 13:38	2023/10/19 15:59	2023/10/20 9:52	2023/10/20 14:28	2023/10/21 10:14	20.2	24.8	44.6
27	2626225	2023/11/3 8:33	2023/11/3 10:51	2023/11/4 9:57	2023/11/5 8:51	2023/11/6 14:21	25.4	48.3	77.8
28	1681995	2023/11/3 11:31	2023/11/3 15:43	2023/11/4 10:07	2023/11/5 8:55	2023/11/6 14:22	22.6	45.4	74.9
29	487966	2023/11/27 23:18	2023/11/28 7:49	2023/11/28 16:27	2023/11/29 9:14	2023/11/30 14:59	17.2	33.9	63.7
30	1910503	2023/11/30 16:36	2023/12/1 7:56	2023/12/2 9:53	2023/12/2 14:42	2023/12/3 14:18	41.3	46.1	69.7
31	2644482	2023/11/6 6:05	2023/11/6 7:59	2023/11/7 8:36	2023/11/8 9:20	2023/11/9 14:06	26.5	51.3	80.0
32	2335924	2023/10/7 14:23	2023/10/7 15:56	2023/10/8 9:01	2023/10/8 15:06	2023/10/9 10:29	18.6	24.7	44.1
33	2628903	2023/10/2 9:42	2023/10/2 11:12	2023/10/4 9:47	2023/10/6 9:34	2023/10/6 11:15	48.1	95.9	97.6

34	1313730	2023/11/22 14:42	2023/11/22 15:53	2023/11/23 9:26	2023/11/24 9:09	2023/11/24 13:52	18.7	42.5	47.2
35	2505503	2023/10/6 20:58	2023/10/7 8:54	2023/10/7 7:31	2023/10/8 9:25	2023/10/9 10:31	10.6	36.5	61.6
36	2488902	2023/11/21 21:49	2023/11/22 8:57	2023/11/22 16:58	2023/11/24 10:33	2023/11/24 13:54	19.2	60.7	64.1
37	2631410	2023/10/10 15:20	2023/10/11 7:49	2023/10/12 9:53	2023/10/12 15:18	2023/10/13 14:40	42.6	48.0	71.3
38	2645140	2023/11/30 12:11	2023/11/30 16:13	2023/12/1 8:41	2023/12/1 14:25	2023/12/2 14:28	20.5	26.2	50.3
39	754375	2023/12/13 19:31	2023/12/14 8:04	2023/12/14 16:00	2023/12/16 10:34	2023/12/17 11:01	20.5	63.1	87.5
40	393660	2023/11/13 23:49	2023/11/14 7:57	2023/11/15 10:01	2023/11/16 9:35	2023/11/16 10:39	34.2	57.8	58.8
41	2476586	2023/10/16 11:31	2023/10/16 11:49	2023/10/17 9:00	2023/10/17 15:10	2023/10/18 14:41	21.5	27.7	51.2
42	1755757	2023/11/12 9:06	2023/11/12 10:54	2023/11/13 9:15	2023/11/15 11:10	2023/11/15 14:46	24.2	74.1	77.7
43	2662048	2023/12/12 19:37	2023/12/13 8:14	2023/12/15 14:44	2023/12/16 8:56	2023/12/17 10:58	67.1	85.3	111.4
44	2265042	2023/10/19 6:23	2023/10/19 8:05	2023/10/20 10:10	2023/10/20 14:29	2023/10/21 10:30	27.8	32.1	52.1
45	2654459	2023/11/27 16:42	2023/11/28 7:50	2023/11/28 14:49	2023/11/29 9:12	2023/11/30 14:04	22.1	40.5	69.4
46	1959285	2023/10/7 6:44	2023/10/7 8:09	2023/10/8 9:11	2023/10/8 14:58	2023/10/9 14:29	26.5	32.2	55.8
47	2648276	2023/11/13 22:30	2023/11/14 7:58	2023/11/15 9:33	2023/11/16 9:31	2023/11/16 10:37	35.1	59.0	60.1
48	1386035	2023/12/4 19:28	2023/12/5 8:01	2023/12/5 17:04	2023/12/6 8:41	2023/12/7 15:04	21.6	37.2	67.6
49	2071423	2023/11/6 14:57	2023/11/6 15:53	2023/11/7 8:40	2023/11/7 14:57	2023/11/8 14:20	17.7	24.0	47.4
50	2666250	2023/12/23 21:47	2023/12/24 8:30	2023/12/25 9:07	2023/12/25 13:56	2023/12/26 11:21	35.3	40.2	61.6
51	2660775	2023/12/11 16:48	2023/12/12 8:53	2023/12/12 14:11	2023/12/13 8:55	2023/12/14 10:51	21.4	40.1	66.1
52	2605343	2023/12/30 16:32	2023/12/31 9:02	2023/12/31 14:57	2024/1/1 8:56	2024/1/2 11:07	22.4	40.4	66.6
53	1621670	2023/11/5 17:58	2023/11/6 7:59	2023/11/7 10:30	2023/11/7 14:59	2023/11/8 14:19	40.5	45.0	68.4
54	2633059	2023/10/11 21:11	2023/10/12 8:54	2023/10/13 9:56	2023/10/13 15:09	2023/10/14 14:47	36.8	42.0	65.6
55	2650338	2023/11/18 17:29	2023/11/19 9:08	2023/11/20 10:04	2023/11/20 14:33	2023/11/21 15:01	40.6	45.1	69.5
56	769348	2023/10/13 13:44	2023/10/13 16:03	2023/10/14 9:19	2023/10/14 15:28	2023/10/15 11:35	19.6	25.7	45.9
57	2650323	2023/11/19 5:41	2023/11/19 9:07	2023/11/20 8:58	2023/11/20 14:34	2023/11/21 14:22	27.3	32.9	56.7
58	2651784	2023/11/22 3:00	2023/11/22 7:53	2023/11/23 9:22	2023/11/23 14:08	2023/11/24 14:01	30.4	35.1	59.0
59	2649582	2023/11/16 16:27	2023/11/16 16:50	2023/11/17 14:25	2023/11/18 9:36	2023/11/19 10:38	22.0	41.2	66.2
60	2649448	2023/11/18 8:45	2023/11/18 10:54	2023/11/20 8:30	2023/11/20 9:14	2023/11/21 13:53	47.8	48.5	77.1
61	2592914	2023/10/17 16:12	2023/10/17 16:23	2023/10/18 10:40	2023/10/19 9:17	2023/10/20 11:14	18.5	41.1	67.0
62	2664456	2023/12/18 14:52	2023/12/18 15:34	2023/12/19 8:16	2023/12/19 14:22	2023/12/20 11:06	17.4	23.5	44.2
63	2629111	2023/10/4 14:49	2023/10/4 16:04	2023/10/5 9:20	2023/10/5 14:23	2023/10/6 11:16	18.5	23.6	44.5
64	2136459	2023/10/17 21:20	2023/10/18 8:55	2023/10/19 9:35	2023/10/19 15:05	2023/10/20 10:57	36.2	41.8	61.6
65	2543009	2023/12/11 21:24	2023/12/12 8:52	2023/12/12 16:28	2023/12/13 8:59	2023/12/15 14:01	19.1	35.6	88.6
66	1634988	2023/11/5 14:58	2023/11/5 15:39	2023/11/8 8:36	2023/11/8 9:14	2023/11/11 10:30	65.6	66.3	139.5
67	2649770	2023/11/17 9:42	2023/11/17 10:53	2023/11/18 9:02	2023/11/19 9:39	2023/11/19 11:25	23.3	48.0	49.7
68	2202764	2023/10/29 9:38	2023/10/29 10:55	2023/10/30 8:53	2023/10/30 14:54	2023/10/31 11:33	23.3	29.3	49.9
69	2522996	2023/11/9 20:20	2023/11/10 8:03	2023/11/10 15:26	2023/11/11 9:14	2023/11/13 14:10	19.1	36.9	89.8
70	658510	2023/10/10 14:39	2023/10/10 16:03	2023/10/11 11:24	2023/10/12 9:24	2023/10/13 14:28	20.8	42.8	71.8
71	2637049	2023/10/20 15:15	2023/10/20 16:06	2023/10/21 9:17	2023/10/22 9:17	2023/10/23 10:03	18.0	42.0	66.8
72	2663434	2023/12/18 15:50	2023/12/18 16:29	2023/12/19 8:38	2023/12/19 14:24	2023/12/20 10:57	16.8	22.6	43.1
73	2535399	2023/10/9 8:01	2023/10/9 8:55	2023/10/10 8:44	2023/10/10 15:16	2023/10/11 14:47	24.7	31.3	54.8
74	2536311	2023/12/7 13:17	2023/12/7 13:53	2023/12/8 8:44	2023/12/8 14:04	2023/12/9 10:47	19.5	24.8	45.5
75	2646918	2023/11/15 15:13	2023/11/15 15:36	2023/11/16 8:39	2023/11/19 9:45	2023/11/19 11:30	17.4	90.5	92.3
76	2650643	2023/11/19 19:02	2023/11/20 8:06	2023/11/21 8:49	2023/11/22 9:07	2023/11/23 10:20	37.8	62.1	87.3
77	667481	2023/10/13 1:43	2023/10/13 8:22	2023/10/13 16:44	2023/10/14 9:36	2023/10/15 11:34	15.0	31.9	57.9
78	2650223	2023/12/3 12:17	2023/12/3 15:23	2023/12/5 10:15	2023/12/6 8:39	2023/12/6 9:55	46.0	68.4	69.6
79	2554300	2023/12/1 9:31	2023/12/1 10:00	2023/12/4 9:54	2023/12/6 8:48	2023/12/6 9:51	72.4	119.3	120.3
80	2533844	2023/11/15 14:48	2023/11/15 16:52	2023/11/17 16:53	2023/11/18 9:33	2023/11/19 11:39	50.1	66.8	92.9

81	2319216	2023/11/7 11:34	2023/11/7 11:48	2023/11/12 9:29	2023/11/13 9:00	2023/11/14 11:28	117.9	141.4	167.9
82	2329849	2023/12/11 10:38	2023/12/11 11:41	2023/12/12 14:15	2023/12/13 8:53	2023/12/14 10:45	27.6	46.3	72.1
83	1666952	2023/11/25 21:45	2023/11/26 8:52	2023/11/27 14:23	2023/11/28 8:38	2023/11/29 15:22	40.6	58.9	89.6
84	1775531	2023/10/18 11:51	2023/10/18 16:02	2023/10/19 10:58	2023/10/20 10:15	2023/10/22 10:07	23.1	46.4	94.3
85	2390590	2023/10/26 16:09	2023/10/26 16:51	2023/10/31 9:38	2023/10/31 15:46	2023/11/1 13:44	113.5	119.6	141.6
86	2628828	2023/10/2 0:25	2023/10/2 9:04	2023/10/3 9:38	2023/10/3 14:40	2023/10/4 15:10	33.2	38.3	62.8
87	2629651	2023/10/5 16:59	2023/10/6 8:49	2023/10/7 8:45	2023/10/7 15:17	2023/10/8 10:00	39.8	46.3	65.0
88	2629125	2023/10/17 13:53	2023/10/17 16:00	2023/10/18 10:15	2023/10/18 14:50	2023/10/19 15:06	20.4	25.0	49.2
89	2622400	2023/10/10 6:52	2023/10/10 8:54	2023/10/11 8:47	2023/10/12 9:23	2023/10/13 14:39	25.9	50.5	79.8
90	2641961	2023/11/10 9:36	2023/11/10 11:03	2023/11/11 9:24	2023/11/11 14:13	2023/11/12 10:43	23.8	28.6	49.1
91	2439508	2023/10/2 7:29	2023/10/2 9:03	2023/10/3 10:28	2023/10/4 9:08	2023/10/5 14:30	27.0	49.7	79.0
92	2539361	2023/11/30 17:20	2023/12/1 8:57	2023/12/1 16:22	2023/12/2 8:50	2023/12/3 10:09	23.0	39.5	64.8
93	1066771	2023/11/8 6:24	2023/11/8 7:48	2023/11/9 9:13	2023/11/9 16:32	2023/11/10 15:39	26.8	34.1	57.2
94	2542942	2023/10/30 10:28	2023/10/30	2023/10/31 16:39	2023/11/1 9:32	2023/11/3 13:47	30.2	47.1	99.3
95	2631259	2023/10/9 5:57	2023/10/9 8:04	2023/10/10 17:20	2023/10/11 9:17	2023/10/12 10:58	35.4	51.3	77.0
96	905588	2023/11/17 17:18	2023/11/18 8:17	2023/11/19 9:01	2023/11/19 14:05	2023/11/21 14:10	39.7	44.8	92.9
97	1869874	2023/10/12 14:10	2023/11/12 15:34	2023/10/13 9:01	2023/10/13 15:06	2023/10/14 14:46	18.8	24.9	48.6
98	2663227	2023/12/15 9:16	2023/12/15 10:16	2023/12/17 9:53	2023/12/19 9:41	2023/12/19 10:30	48.6	96.4	97.2
99	2665316	2023/12/21 5:55	2023/12/21 8:11	2023/12/22 9:49	2023/12/22 14:57	2023/12/23 11:46	27.9	33.0	53.8
100	2154602	2023/12/12 21:27	2023/12/13 8:59	2023/12/14 9:32	2023/12/14 14:38	2023/12/15 13:59	36.1	41.2	64.5

Table 3 Laboratory endpoints of Phase II Pre-op

ID	Admission number	Blood culture collection	Blood culture loading time	Gram staining report	Species identification	Final AST report time	TAT for Gram-staining reports of positive cultures	Time to microbial species identification for positive cultures	Total TAT from specimen collection to clinician receipt of reports
1	2659898	2023/12/10 1:54	2023/12/10 7:57	2023/12/11 9:27	2023/12/11 14:46	2023/12/12 14:43	31.5	36.9	60.8
2	2660775	2023/12/11 16:48	2023/12/12 8:53	2023/12/12 14:11	2023/12/13 8:55	2023/12/14 10:51	21.4	40.1	66.0
3	2662694	2023/12/13 19:14	2023/12/14 8:03	2023/12/15 10:50	2023/12/15 14:23	2023/12/17 11:09	39.6	43.2	87.9
4	2665006	2023/12/19 19:49	2023/12/20 8:24	2023/12/22 9:47	2023/12/22 14:54	2023/12/24 10:39	62.0	67.1	110.8
5	2655692	2023/12/20 19:25	2023/12/21 8:12	2023/12/22 9:45	2023/12/22 14:55	2023/12/23 11:46	38.3	43.5	64.3
6	689901	2024/1/1 17:39	2024/1/2 7:45	2024/1/3 13:55	2024/1/4 9:43	2024/1/5 11:08	44.3	64.1	89.5
7	554034	2024/1/1 21:26	2024/1/2 8:57	2024/1/6 9:01	2024/1/6 13:57	2024/1/7 10:53	107.6	112.5	133.5
8	2653098	2024/1/2 22:03	2024/1/3 8:55	2024/1/4 9:08	2024/1/4 13:51	2024/1/5 11:19	35.1	39.8	61.3
9	1264419	2024/1/3 16:55	2024/1/4 8:53	2024/1/5 13:54	NA	2024/1/6 10:15	45.0	NA	65.3
10	1188427	2024/1/6 18:12	2024/1/7 8:01	2024/1/7 16:03	NA	2024/1/9 11:03	21.9	NA	64.9
11	703684	2024/1/12 17:19	2024/1/13 9:04	2024/1/14 9:39	2024/1/15 8:58	2024/1/16 15:30	40.3	NA	94.2
12	2679696	2024/1/13 22:25	2024/1/14 8:02	2024/1/17 10:36	2024/1/18 14:50	2024/1/18 14:50	84.2	112.4	112.4
13	2610503	2024/1/14 19:32	2024/1/15 7:41	2024/1/16 8:17	2024/1/16 14:08	2024/1/18 15:02	36.8	42.6	91.5
14	2681068	2024/1/17 0:11	2024/1/17 8:13	2024/1/18 8:32	2024/1/18 14:00	2024/1/19 10:57	32.4	37.8	58.8
15	1030285	2024/1/18 16:28	2024/1/19 8:02	2024/1/19 16:01	2024/1/20 9:11	2024/1/21 11:03	23.6	40.7	66.6
16	2682990	2024/1/21 19:20	2024/1/22 8:29	2024/1/22 15:18	2024/1/23 8:53	2024/1/24 13:59	20.0	37.6	66.7
17	2417240	2024/1/27 19:04	2024/1/28 9:02	2024/1/29 10:05	2024/1/29 15:14	2024/1/30 10:39	39.0	44.2	63.6
18	935527	2024/1/29 17:51	2024/1/30 7:55	2024/1/31 10:47	2024/2/2 8:44	2024/2/2 10:53	40.9	86.9	89.0
19	464237	2024/2/1 19:20	2024/2/2 7:50	2024/2/5 7:02	2024/2/6 9:59	2024/2/6 10:51	83.7	110.7	111.5
20	2269888	2024/2/1 20:43	2024/2/2 7:52	2024/2/2 14:48	NA	2024/2/4 11:01	18.1	NA	62.3
21	690740	2024/2/3 18:31	2024/2/4 8:04	2024/2/6 10:10	2024/2/6 14:53	2024/2/7 10:32	63.7	68.4	88.0
22	2688790	2024/2/4 17:12	2024/2/5 8:00	2024/2/6 15:24	2024/2/8 8:13	2024/2/8 8:56	46.2	87.0	87.7
23	2688795	2024/2/4 17:17	2024/2/5 8:01	2024/2/6 10:03	2024/2/8 7:29	2024/2/8 8:58	40.8	86.2	87.7
24	2688480	2024/2/4 16:27	2024/2/5 8:51	2024/2/7 9:44	2024/2/7 13:43	2024/2/8 8:58	65.3	69.3	88.5
25	2011299	2024/2/5 21:21	2024/2/6 7:54	2024/2/7 9:47	2024/2/7 13:44	2024/2/8 9:06	36.4	40.4	59.8
26	2666969	2024/2/7 20:19	2024/2/8 8:07	2024/2/9 8:13	2024/2/9 14:16	2024/2/10 10:54	35.9	42.0	62.6
27	2689774	2024/2/8 21:27	2024/2/9 7:57	2024/2/9 16:12	2024/2/10 9:34	2024/2/11 11:37	18.8	36.1	62.2
28	2689867	2024/2/9 17:40	2024/2/10 8:59	2024/2/11 13:29	2024/2/12 9:04	2024/2/14 14:14	43.8	63.4	116.6
29	609743	2024/2/10 19:26	2024/2/11 7:57	2024/2/12 10:09	2024/2/12 13:48	2024/2/13 11:31	38.7	42.4	64.1
30	1746477	2024/2/11 19:24	2024/2/12 9:31	2024/2/13 10:27	2024/2/13 14:41	2024/2/14 14:15	39.1	43.3	66.9
31	2606574	2024/2/14 16:08	2024/2/15 9:07	2024/2/16 15:15	2024/2/18 10:07	2024/2/18 10:09	47.1	90.0	90.0

32	2690856	2024/2/16 23:21	2024/2/17 8:37	2024/2/20 10:17	NA	2024/2/22 10:28	82.9	NA	131.1
33	2689378	2024/2/18 16:52	2024/2/19 7:56	NA	2024/2/21 9:57	2024/2/22 10:38	NA	65.1	89.8
34	2541501	2024/2/18 16:52	2024/2/19 8:58	2024/2/21 9:20	2024/2/22 9:18	2024/2/24 11:17	64.5	88.4	138.4
35	2692911	2024/2/18 17:46	2024/2/19 9:10	2024/2/23 8:39	2024/2/2314:5:21	2024/2/24 11:08	110.9	NA	137.4
36	2693547	2024/2/19 15:10	2024/2/20 8:56	2024/2/20 14:46	2024/2/21 10:24	2024/2/22 9:51	23.6	43.2	66.7
37	947030	2024/2/23 16:24	2024/2/24 8:55	2024/2/24 14:24	2024/2/25 8:55	2024/2/26 8:17	22.0	40.5	63.9
38	2696494	2024/2/26 19:33	2024/2/27 7:53	2024/2/27 16:10	2024/2/28 9:14	2024/2/29 14:18	20.6	37.7	66.8
39	2685308	2024/2/29 17:49	2024/3/1 9:22	2024/3/4 8:29	2024/3/4 14:14	2024/3/5 10:20	86.7	92.4	112.5
40	1598055	2024/3/3 17:58	2024/3/4 7:56	2024/3/4 16:17	2024/3/5 8:24	2024/3/6 10:38	22.3	38.4	64.7
41	2699622	2024/3/4 17:54	2024/3/5 8:17	2024/3/6 16:15	2024/3/7 9:24	2024/3/8 14:23	46.4	63.5	92.5
42	2383592	2024/3/5 15:04	2024/3/6 7:53	2024/3/6 16:17	2024/3/7 9:17	2024/3/8 14:22	25.2	42.2	71.3
43	1006435	2024/3/5 19:22	2024/3/6 7:57	2024/3/7 9:34	2024/3/7 13:46	2024/3/8 14:26	38.2	42.4	67.1
44	2700551	2024/3/6 17:45	2024/3/7 8:46	2024/3/8 16:59	2024/3/11 10:02	2024/3/11 14:10	47.2	112.3	116.4
45	701243	2024/3/6 21:58	2024/3/7 8:59	2024/3/8 9:13	2024/3/8 14:07	2024/3/9 11:01	35.3	40.2	61.1
46	2681024	2024/3/7 20:24	2024/3/8 8:01	2024/3/9 8:52	2024/3/10 8:04	2024/3/11 14:30	36.5	59.7	90.1
47	2699627	2024/3/7 18:08	2024/3/8 8:57	NA	2024/3/12 14:11	2024/3/13 10:22	NA	116.1	136.2
48	2447661	2024/3/8 15:40	2024/3/9 8:07	2024/3/10 8:26	NA	2024/3/12 10:52	40.8	NA	91.2
49	496832	2024/3/8 20:01	2024/3/9 8:08	2024/3/10 16:27	2024/3/11 9:45	2024/3/12 11:44	44.4	61.7	87.7
50	2686687	2024/3/9 22:42	2024/3/10 8:05	2024/3/11 9:39	2024/3/11 15:15	2024/3/12 11:15	35.0	40.6	60.6
51	2699721	2024/3/14 18:17	2024/3/15 8:05	2024/3/15 16:01	2024/3/16 9:00	2024/3/17 10:22	21.7	38.7	64.1
52	1476119	2024/3/16 16:35	2024/3/17 7:53	2024/3/17 14:21	NA	2024/3/19 14:25	21.8	NA	69.8
53	2687832	2024/3/16 23:04	2024/3/17 8:56	2024/3/18 10:08	NA	2024/3/20 11:15	35.1	NA	84.2
54	2705595	2024/3/16 22:01	2024/3/17 8:57	2024/3/18 10:04	2024/3/18 14:24	2024/3/19 14:35	36.1	40.4	64.6
55	2705916	2024/3/17 20:34	2024/3/18 8:32	2024/3/19 9:17	2024/3/20 8:55	2024/3/21 14:46	36.7	60.4	90.2
56	2705825	2024/3/17 16:16	2024/3/18 9:45	2024/3/19 9:23	2024/3/19 15:56	2024/3/20 11:16	41.1	47.7	67.0
57	2706316	2024/3/18 15:53	2024/3/19 8:49	2024/3/20 10:02	NA	2024/3/21 11:23	42.2	NA	67.5
58	2692340	2024/3/19 19:48	2024/3/20 8:02	2024/3/21 9:42	2024/3/21 14:08	2024/3/22 16:26	37.9	42.3	68.6
59	2707798	2024/3/21 19:39	2024/3/22 9:02	2024/3/23 9:26	2024/3/23 14:21	2024/3/24 11:10	37.8	42.7	63.5
60	2250990	2024/3/22 15:16	2024/3/23 7:43	2024/3/23 16:15	2024/3/24 8:43	2024/3/25 11:40	25.0	41.5	68.4
61	2701814	2024/3/22 23:12	2024/3/23 7:44	2024/3/23 16:17	2024/3/24 8:40	2024/3/26 10:53	17.1	33.5	83.7
62	2314177	2024/3/24 3:44	2024/3/24 8:18	2024/3/24 15:14	2024/3/25 8:32	2024/3/26 10:14	11.5	28.8	54.5
63	2709441	2024/3/25 16:41	2024/3/26 7:53	2024/3/26 14:21	2024/3/27 8:57	2024/3/28 10:41	21.7	40.3	66.0
64	2710060	2024/3/26 21:47	2024/3/27 9:00	2024/3/28 16:15	2024/3/29 9:05	2024/3/30 11:14	42.5	59.3	85.5
65	2659720	2024/3/28 20:54	2024/3/29 9:09	2024/3/30 8:40	2024/3/30 8:40	2024/3/31 10:45	35.8	35.8	61.9
66	1556422	2024/3/31 22:43	2024/4/1 8:59	2024/4/2 9:26	2024/4/2 14:50	2024/4/3 16:19	34.7	40.1	65.6
67	1383682	2024/4/1 18:40	2024/4/2 8:07	2024/4/2 16:39	NA	2024/4/4 12:15	22.0	NA	65.6
68	2114109	2024/4/1 22:13	2024/4/2 9:16	2024/4/3 10:07	2024/4/4 9:10	2024/4/5 10:09	35.9	59.0	83.9
69	2708146	2024/4/3 0:18	2024/4/3 8:22	2024/4/3 17:33	2024/4/4 9:05	2024/4/5 10:38	17.3	32.8	58.3
70	1041979	2024/4/3 18:53	2024/4/4 9:03	2024/4/4 16:19	2024/4/5 8:57	2024/4/8 11:30	21.4	38.1	112.6
71	2712765	2024/4/4 17:10	2024/4/5 8:01	2024/4/6 9:48	2024/4/7 9:09	2024/4/7 10:21	40.6	64.0	65.2
72	2636923	2024/4/4 16:44	2024/4/5 8:52	2024/4/6 14:12	2024/4/7 8:59	2024/4/8 11:29	45.5	64.3	90.8
73	2716214	2024/4/6 17:05	2024/4/7 8:17	2024/4/8 8:52	2024/4/8 8:53	2024/4/9 13:56	39.8	39.8	68.9
74	2716190	2024/4/9 21:34	2024/4/10 8:17	2024/4/11 9:37	2024/4/11 13:58	2024/4/12 13:49	36.1	40.4	64.3
75	1588671	2024/4/11 21:27	2024/4/12 8:16	2024/4/13 10:53	2024/4/14 8:35	2024/4/15 13:50	37.4	59.1	88.4
76	2396543	2024/4/12 21:27	2024/4/13 7:53	2024/4/14 8:53	2024/4/14 14:06	2024/4/16 10:42	35.4	40.7	85.3
77	2554070	2024/4/17 17:58	2024/4/18 8:05	2024/4/19 9:40	2024/4/19 14:18	2024/4/20 10:29	39.7	44.3	64.5

78	2719470	2024/4/17 21:22	2024/4/18 9:08	2024/4/19 9:45	2024/4/19 14:18	2024/4/20 10:26	36.4	40.9	61.1
79	2720334	2024/4/18 23:57	2024/4/19 7:59	2024/4/20 9:11	2024/4/20 14:18	2024/4/22 13:48	33.2	38.3	85.8
80	2719976	2024/4/18 18:02	2024/4/19 9:18	2024/4/20 9:15	2024/4/2014:20	2024/4/22 10:47	39.2	NA	88.8
81	2713841	2024/4/20 19:16	2024/4/21 8:39	2024/4/25 8:21	NA	2024/4/27 10:00	109.1	NA	158.7
82	1533443	2024/4/20 20:16	2024/4/21 9:02	2024/4/21 15:56	2024/4/22 9:31	2024/4/23 10:46	19.7	37.2	62.5
83	2720995	2024/4/21 16:34	2024/4/22 8:57	2024/4/23 9:47	2024/4/23 13:41	2024/4/24 10:58	41.2	45.1	66.4
84	668141	2024/4/22 18:35	2024/4/23 8:14	2024/4/24 8:43	2024/4/24 14:18	2024/4/25 11:28	38.1	43.7	64.9
85	2648811	2024/4/22 20:07	2024/4/23 9:13	2024/4/24 8:33	2024/4/24 14:17	2024/4/25 13:43	36.4	42.2	65.6
86	2653445	2024/4/25 21:44	2024/4/26 8:08	2024/4/27 9:03	2024/4/27 13:59	2024/4/28 10:13	35.3	40.3	60.5
87	2723150	2024/4/25 19:09	2024/4/26 8:10	2024/4/26 16:01	2024/4/27 8:19	2024/4/28 11:22	20.9	37.2	64.2
88	2723511	2024/4/28 23:16	2024/4/29 7:58	2024/5/2 8:52	NA	2024/5/4 10:59	81.6	NA	131.7
89	2724210	2024/4/28 22:44	2024/4/29 9:11	2024/5/1 10:14	2024/5/1 14:56	2024/5/2 14:23	59.5	64.2	87.7
90	2721700	2024/4/28 19:15	2024/4/29 9:12	2024/4/30 9:46	2024/5/1 9:30	2024/5/2 14:13	NA	NA	NA

Table 4 Laboratory endpoints of Phase II Post-op

ID	Admission number	Blood culture collection	Blood culture loading time	Gram staining report	Species identification	Final AST report time	TAT for Gram-staining reports of positive cultures	Time to microbial species identification for positive cultures	Total TAT from specimen collection to clinician receipt of reports
1	2727581	2024/5/8 2:22	2024/5/8 3:52	2024/5/9 10:15	2024/5/9 14:52	2024/5/10 15:13	31.9	36.5	60.8
2	1515608	2024/5/10 3:45	2024/5/10 4:31	2024/5/11 16:11	2024/5/12 9:01	2024/5/13 10:15	36.4	53.3	78.5
3	2726402	2024/5/11 18:30	2024/5/11 19:20	2024/5/12 11:03	NA	2024/5/14 14:18	16.6	NA	67.8
4	2068125	2024/5/12 19:16	2024/5/13 8:12	2024/5/13 16:58	2024/5/14 9:05	2024/5/15 14:26	21.7	37.8	67.2
5	2729973	2024/5/13 17:40	2024/5/13 20:17	2024/5/14 15:54	2024/5/15 9:06	2024/5/16 10:17	22.2	39.4	64.6
6	2605903	2024/5/13 20:13	2024/5/13 21:24	2024/5/14 10:04	2024/5/14 14:51	2024/5/15 14:41	13.8	18.6	42.5
7	2114109	2024/5/14 17:41	2024/5/14 18:29	2024/5/16 10:09	NA	2024/5/21 10:54	40.5	NA	161.2
8	2731034	2024/5/15 21:16	2024/5/15 23:08	2024/5/16 14:36	2024/5/16 16:06	2024/5/17 11:18	17.3	18.8	38.0
9	2730233	2024/5/16 17:57	2024/5/16 18:34	NA	2024/5/18 9:36	2024/5/19 11:36	NA	39.7	65.7
10	2098329	2024/5/16 23:22	2024/5/17 1:55	2024/5/17 15:12	2024/5/18 9:27	2024/5/19 10:58	15.8	34.1	59.6
11	2730092	2024/5/17 22:42	2024/5/18 8:00	2024/5/18 11:30	2024/5/19 8:42	2024/5/20 11:38	12.8	34.0	60.9
12	2732113	2024/5/18 18:01	2024/5/18 19:38	2024/5/20 8:40	2024/5/21 10:39	2024/5/21 10:48	38.7	64.6	64.8
13	1674759	2024/5/20 18:39	2024/5/20 19:29	2024/5/23 9:27	2024/5/24 9:42	2024/5/25 11:19	62.8	87.1	112.7
14	2733037	2024/5/21 0:27	2024/5/21 1:21	2024/5/22 9:11	2024/5/24 9:12	2024/5/24 14:36	32.7	80.8	86.2
15	2726465	2024/5/22 17:42	2024/5/22 18:09	2024/5/23 9:44	2024/5/23 14:18	2024/5/24 14:34	16.0	20.6	44.9
16	2733717	2024/5/22 21:33	2024/5/22 21:37	2024/5/23 15:12	2024/5/25 10:36	2024/5/25 10:38	17.7	61.1	61.1
17	677708	2024/5/25 1:28	2024/5/25 2:51	2024/5/26 11:14	2024/5/27 9:22	2024/5/28 10:14	33.8	55.9	80.8
18	2734582	2024/5/25 3:33	2024/5/25 16:04	2024/5/26 8:58	NA	2024/5/27 10:18	29.4	NA	54.8
19	1864353	2024/5/27 19:30	2024/5/27 21:09	2024/5/28 9:22	2024/5/28 14:44	2024/5/29 15:13	13.9	19.2	43.7
20	534939	2024/5/28 3:00	204/5/28 3:49	2024/5/28 15:26	2024/5/29 9:43	2024/5/30 11:30	12.4	30.7	56.5
21	2736301	2024/5/28 23:43	2024/5/29 0:07	2024/5/29 16:51	2024/5/30 8:23	2024/5/31 15:16	17.1	32.7	63.6
22	2737127	2024/5/30 18:01	2024/5/30 19:22	2024/5/31 9:45	2024/6/1 11:32	2024/6/1 11:35	15.7	41.5	41.6
23	2735922	2024/5/31 17:21	2024/5/31 17:47	2024/6/1 17:02	2024/6/2 9:04	2024/6/3 10:25	23.7	39.7	65.1
24	1166970	2024/5/31 19:56	2024/5/31 20:30	2024/6/2 9:33	2024/6/2 15:01	2024/6/3 10:18	37.6	43.1	62.4
25	2476396	2024/6/1 22:01	2024/6/1 22:40	2024/6/2 10:22	2024/6/2 14:58	2024/6/3 10:27	12.4	17.0	36.4
26	1598055	2024/6/4 22:03	2024/6/4 20:24	2024/6/6 9:42	2024/6/6 16:42	2024/6/8 14:54	35.7	42.7	88.9
27	2319636	2024/6/4 22:43	2024/6/5 0:10	2024/6/5 10:25	2024/6/5 15:17	2024/6/6 9:58	11.7	16.6	35.3
28	496832	2024/6/5 3:04	2024/6/5 3:47	2024/6/6 9:35	2024/6/7 8:47	2024/6/8 14:37	30.5	53.7	83.6
29	2739189	2024/6/5 2:34	2024/6/5 3:48	2024/6/5 16:41	2024/6/6 9:16	2024/6/7 14:46	14.1	30.7	60.2
30	1880629	2024/6/5 22:37	2024/6/6 0:34	2024/6/6 10:47	2024/6/6 16:41	2024/6/7 10:05	12.2	18.1	35.5
31	2739955	2024/6/6 18:19	2024/6/6 19:08	2024/6/7 9:24	2024/6/9 9:33	2024/6/9 10:08	15.1	63.2	63.8
32	2736777	2024/6/7 18:57	20244/6/8 8:17	2024/6/8 17:38	2024/6/9 9:11	2024/6/11 10:15	22.7	38.2	87.3
33	2740794	2024/6/10 4:51	2024/6/10 5:56	2024/6/11 9:22	2024/6/11 15:13	2024/6/12 10:39	28.5	34.4	53.8
34	2068125	2024/6/11 3:22	2024/6/11 7:59	2024/6/12 16:07	2024/6/14 9:09	2024/6/14 10:07	36.8	77.8	78.8
35	2742331	2024/6/13 0:14	2024/6/13 1:12	2024/6/13 10:16	2024/6/13 15:01	2024/6/16 8:27	10.0	14.8	80.2
36	2739130	2024/6/14 22:40	2024/6/14 22:54	2024/6/15 11:20	2024/6/16 9:09	2024/6/17 14:47	12.7	34.5	64.1

37	2701751	2024/6/16 17:49	2024/6/16 18:42	2024/6/17 9:08	2024/6/17 14:43	2024/6/18 10:14	15.3	20.9	40.4
38	2678506	2024/6/16 21:43	2024/6/16 23:15	2024/6/17 16:22	2024/6/20 11:11	2024/6/22 9:40	18.6	85.5	132.0
39	2704385	2024/6/17 19:13	2024/6/17 20:19	2024/6/18 15:42	2024/6/20 9:34	2024/6/20 9:37	20.5	62.4	62.4
40	2744294	2024/6/18 1:23	2024/6/18 2:05	2024/6/19 16:10	2024/6/20 9:05	2024/6/23 14:29	38.8	55.7	133.1
41	2744747	2024/6/19 5:36	2024/6/19 9:38	2024/6/23 10:00	2024/6/24 9:43	2024/6/25 14:49	100.4	124.1	153.2
42	1472678	2024/6/19 17:44	2024/6/19 18:31	2024/6/20 9:30	2024/6/20 15:07	2024/6/21 9:09	15.8	21.4	39.4
43	2720521	2024/6/19 21:48	2024/6/19 22:27	2024/6/20 15:59	2024/6/21 9:10	2024/6/22 10:52	18.2	35.4	61.1
44	2744518	2024/6/20 0:42	2024/6/20 1:11	2024/6/21 9:40	2024/6/22 9:02	2024/6/23 11:14	33.0	56.3	82.5
45	2742261	2024/6/20 1:02	2024/6/20 6:31	2024/6/20 15:29	2024/6/21 8:56	2024/6/22 10:53	14.5	31.9	57.9
46	2571720	2024/6/20 20:26	2024/6/20 21:24	2024/6/21 9:23	2024/6/24 11:05	2024/6/24 11:10	13.0	86.7	86.7
47	2068125	2024/6/21 2:29	2024/6/21 2:34	2024/6/22 10:08	2024/6/23 5:52	2024/6/24 10:58	31.7	51.4	80.5
48	839459	2024/6/21 2:01	2024/6/21 2:39	2024/6/21 15:52	2024/6/22 9:03	2024/6/23 11:08	13.9	31.0	57.1
49	2746311	2024/6/22 23:22	2024/6/22 23:55	2024/6/23 17:14	2024/6/24 9:32	2024/6/25 14:08	17.9	34.2	62.8
50	2746324	2024/6/23 0:41	2024/6/23 1:19	2024/6/24 14:24	2024/6/24 14:46	2024/6/25 11:30	37.7	38.1	58.8
51	2746104	2024/6/23 6:50	2024/6/23 4:47	2024/6/23 17:01	2024/6/24 9:31	2024/6/25 14:21	10.2	26.7	55.5
52	2703582	2024/6/23 17:35	2024/6/23 18:01	2024/6/24 11:24	2024/6/24 14:49	2024/6/25 14:24	17.8	21.2	44.8
53	1101486	2024/6/23 18:59	2024/6/23 19:48	2024/6/24 14:41	2024/6/27 11:37	2024/6/27 11:39	19.7	88.6	88.7
54	2086131	2024/6/23 21:12	2024/6/23 22:02	2024/6/24 14:36	2024/6/25 9:05	2024/6/26 15:37	17.4	35.9	66.4
55	1970178	2024/6/23 22:23	2024/6/23 23:22	2024/6/24 11:17	2024/6/24 14:51	2024/6/25 14:24	12.9	16.5	40.0
56	1100968	2024/6/24 20:11	2024/6/24 21:05	2024/6/26 14:52	2024/6/26 14:54	2024/6/27 11:34	42.7	42.7	63.4
57	2747567	2024/6/25 18:51	2024/6/25 19:26	2024/6/26 9:25	2024/6/26 14:47	2024/6/27 11:28	14.6	19.9	40.6
58	1693385	2024/6/26 17:48	2024/6/26 18:24	2024/6/28 8:50	2024/6/28 14:46	2024/6/29 17:46	39.0	45.0	72.0
59	2619593	2024/6/26 17:59	2024/6/26 19:34	2024/6/27 10:21	2024/6/28 9:26	2024/6/30 11:49	16.4	39.5	89.8
60	2747539	2024/6/26 20:49	2024/6/26 19:13	2024/6/27 14:39	2024/6/28 9:07	2024/6/30 11:40	17.8	36.3	86.9
61	2739928	2024/6/26 22:23	2024/6/26 22:59	2024/6/28 14:48	2024/6/28 10:00	2024/6/30 11:09	40.4	35.6	84.8
62	2748115	2024/6/26 22:49	2024/6/27 0:05	2024/6/27 10:23	2024/6/28 9:27	2024/6/29 17:40	11.6	34.6	66.9
63	2583334	2024/6/28 22:00	2024/6/29 7:51	2024/6/30 16:18	2024/7/1 9:01	2024/7/2 10:10	42.3	59.0	84.2
64	2748874	2024/6/29 2:33	2024/6/29 7:53	2024/6/30 9:40	2024/7/1 8:57	2024/7/2 10:14	31.1	54.4	79.7
65	1960381	2024/6/29 19:35	2024/6/30 7:12	2024/6/30 17:24	2024/7/1 8:59	2024/7/2 9:45	21.8	37.4	62.2
66	2748780	2024/6/30 0:07	2024/6/30 7:16	2024/7/1 9:19	2024/7/1 14:34	2024/7/2 10:56	33.2	38.5	58.8
67	2749142	2024/6/29 20:53	2024/6/30 7:18	2024/6/30 16:09	2024/7/1 9:01	2024/7/2 10:06	19.3	36.1	61.2
68	1693385	2024/6/30 19:45	2024/6/30 21:26	2024/7/2 8:43	2024/7/2 14:55	2024/7/3 10:11	37.0	43.2	62.4
69	2751005	2024/7/3 18:20	2024/7/3 18:38	2024/7/4 9:24	2024/7/4 14:30	2024/7/5 14:10	15.1	20.2	43.8
70	2071098	2024/7/7 20:45	2024/7/8 7:57	2024/7/8 15:17	2024/7/9 9:06	2024/7/11 14:25	18.5	36.4	89.7
71	2752958	2024/7/8 18:32	2024/7/8 19:06	2024/7/9 10:39	2024/7/9 14:42	2024/7/10 10:31	16.1	20.2	40.0
72	2753019	2024/7/8 23:08	2024/7/8 23:59	2024/7/9 11:20	2024/7/914:41	2024/7/10 9:52	12.2	NA	34.7
73	2385072	2024/7/10 18:50	2024/7/10 20:26	2024/7/14 9:16	2024/7/15 8:52	2024/7/16 10:27	86.4	110.0	135.6
74	654420	2024/7/11 0:54	2024/7/11 1:42	2024/7/11 16:14	2024/7/12 9:17	2024/7/14 14:41	15.3	32.4	85.8
75	1064137	2024/7/12 20:43	2024/7/12 21:34	2024/7/13 15:39	2024/7/14 8:49	2024/7/15 14:54	18.9	36.1	66.2
76	2755007	2024/7/13 21:09	2024/7/13 22:04	2024/7/15 10:17	2024/7/16 9:26	2024/7/17 14:16	37.1	60.3	89.1
77	2755324	2024/7/14 19:17	2024/7/14 19:41	2024/7/15 9:24	2024/7/15 15:00	2024/7/16 10:28	14.1	19.7	39.2
78	2449336	2024/7/14 19:04	2024/7/14 19:52	2024/7/15 9:36	2024/7/15 14:59	2024/7/16 10:27	14.5	19.9	39.4
79	2757741	2024/7/19 19:43	2024/7/19 21:11	2024/7/20 10:49	2024/7/20 14:24	2024/7/21 10:51	15.1	18.7	39.1
80	2758078	2024/7/21 1:42	2024/7/21 3:16	2024/7/21 9:56	2024/7/21 14:45	2024/7/22 11:45	8.2	13.1	34.1
81	2756282	2024/7/21 19:41	2024/7/21 20:33	2024/7/22 15:03	2024/7/23 8:55	2024/7/24 14:36	19.4	37.2	66.9
82	2378914	2024/7/21 22:37	2024/7/21 23:34	2024/7/22 11:10	2024/7/23 10:37	2024/7/26 8:13	12.6	36.0	105.6
83	2587431	2024/7/24 22:58	2024/7/24 23:56	2024/7/25 15:07	2024/7/26 9:26	2024/7/27 11:18	16.2	34.5	60.3

84	2634722	2024/7/25 18:22	2024/7/25 19:02	2024/7/26 8:53	2024/7/26 14:14	2024/7/27 15:26	14.5	19.9	45.1
85	2760399	2024/7/25 19:53	2024/7/25 20:53	2024/7/26 17:37	2024/7/27 9:11	2024/7/28 11:35	21.7	37.3	63.7
86	2710535	2024/7/27 18:08	2024/7/27 18:57	2024/7/28 9:33	2024/7/28 14:22	2024/7/29 10:45	15.4	20.2	40.6
87	2761135	2024/7/27 23:23	2024/7/27 23:37	2024/7/29 9:15	2024/7/31 10:06	2024/7/31 14:22	33.9	82.7	87.0
88	2761975	2024/7/29 18:15	2024/7/29 20:01	2024/7/30 9:29	2024/7/31 9:10	2024/8/1 14:40	15.2	38.9	68.4
89	2760431	2024/7/31 19:21	2024/7/31 19:46	2024/8/1 16:24	2024/8/2 9:00	2024/8/3 10:06	21.0	37.6	62.7

Table 5 Laboratory endpoints of Phase III Pre-op

ID	Admission number	Blood culture collection	Blood culture loading time	Gram staining report	Species identification	Final AST report time	TAT for Gram-staining reports of positive cultures	Time to microbial species identification for positive cultures	Total TAT from specimen collection to clinician receipt of reports
1	2727581	2024/5/8 2:22	2024/5/8 3:52	2024/5/9 10:15	2024/5/9 14:52	2024/5/10 15:13	31.9	36.5	60.8
2	1515608	2024/5/10 3:45	2024/5/10 4:31	2024/5/11 16:11	2024/5/12 9:01	2024/5/13 10:15	36.4	53.3	78.5
3	2726402	2024/5/11 18:30	2024/5/11 19:20	2024/5/12 11:03	NA	2024/5/14 14:18	16.6	NA	67.8
4	2068125	2024/5/12 19:16	2024/5/13 8:12	2024/5/13 16:58	2024/5/14 9:05	2024/5/15 14:26	21.7	37.8	67.2
5	2729973	2024/5/13 17:40	2024/5/13 20:17	2024/5/14 15:54	2024/5/15 9:06	2024/5/16 10:17	22.2	39.4	64.6
6	2605903	2024/5/13 20:13	2024/5/13 21:24	2024/5/14 10:04	2024/5/14 14:51	2024/5/15 14:41	13.8	18.6	42.5
7	2114109	2024/5/14 17:41	2024/5/14 18:29	2024/5/16 10:09	NA	2024/5/21 10:54	40.5	NA	161.2
8	2731034	2024/5/15 21:16	2024/5/15 23:08	2024/5/16 14:36	2024/5/16 16:06	2024/5/17 11:18	17.3	18.8	38.0
9	2730233	2024/5/16 17:57	2024/5/16 18:34	NA	2024/5/18 9:36	2024/5/19 11:36	NA	39.7	65.7
10	2098329	2024/5/16 23:22	2024/5/17 1:55	2024/5/17 15:12	2024/5/18 9:27	2024/5/19 10:58	15.8	34.1	59.6
11	2730092	2024/5/17 22:42	2024/5/18 8:00	2024/5/18 11:30	2024/5/19 8:42	2024/5/20 11:38	12.8	34.0	60.9
12	2732113	2024/5/18 18:01	2024/5/18 19:38	2024/5/20 8:40	2024/5/21 10:39	2024/5/21 10:48	38.7	64.6	64.8
13	1674759	2024/5/20 18:39	2024/5/20 19:29	2024/5/23 9:27	2024/5/24 9:42	2024/5/25 11:19	62.8	87.1	112.7
14	2733037	2024/5/21 0:27	2024/5/21 1:21	2024/5/22 9:11	2024/5/24 9:12	2024/5/24 14:36	32.7	80.8	86.2
15	2726465	2024/5/22 17:42	2024/5/22 18:09	2024/5/23 9:44	2024/5/23 14:18	2024/5/24 14:34	16.0	20.6	44.9
16	2733717	2024/5/22 21:33	2024/5/22 21:37	2024/5/23 15:12	2024/5/25 10:36	2024/5/25 10:38	17.7	61.1	61.1
17	677708	2024/5/25 1:28	2024/5/25 2:51	2024/5/26 11:14	2024/5/27 9:22	2024/5/28 10:14	33.8	55.9	80.8
18	2734582	2024/5/25 3:33	2024/5/25 16:04	2024/5/26 8:58	NA	2024/5/27 10:18	29.4	NA	54.8
19	1864353	2024/5/27 19:30	2024/5/27 21:09	2024/5/28 9:22	2024/5/28 14:44	2024/5/29 15:13	13.9	19.2	43.7
20	534939	2024/5/28 3:00	204/5/28 3:49	2024/5/28 15:26	2024/5/29 9:43	2024/5/30 11:30	12.4	30.7	56.5
21	2736301	2024/5/28 23:43	2024/5/29 0:07	2024/5/29 16:51	2024/5/30 8:23	2024/5/31 15:16	17.1	32.7	63.6
22	2737127	2024/5/30 18:01	2024/5/30 19:22	2024/5/31 9:45	2024/6/1 11:32	2024/6/1 11:35	15.7	41.5	41.6
23	2735922	2024/5/31 17:21	2024/5/31 17:47	2024/6/1 17:02	2024/6/2 9:04	2024/6/3 10:25	23.7	39.7	65.1
24	1166970	2024/5/31 19:56	2024/5/31 20:30	2024/6/2 9:33	2024/6/2 15:01	2024/6/3 10:18	37.6	43.1	62.4
25	2476396	2024/6/1 22:01	2024/6/1 22:40	2024/6/2 10:22	2024/6/2 14:58	2024/6/3 10:27	12.4	17.0	36.4
26	1598055	2024/6/4 22:03	2024/6/4 20:24	2024/6/6 9:42	2024/6/6 16:42	2024/6/8 14:54	35.7	42.7	88.9
27	2319636	2024/6/4 22:43	2024/6/5 0:10	2024/6/5 10:25	2024/6/5 15:17	2024/6/6 9:58	11.7	16.6	35.3
28	496832	2024/6/5 3:04	2024/6/5 3:47	2024/6/6 9:35	2024/6/7 8:47	2024/6/8 14:37	30.5	53.7	83.6
29	2739189	2024/6/5 2:34	2024/6/5 3:48	2024/6/5 16:41	2024/6/6 9:16	2024/6/7 14:46	14.1	30.7	60.2
30	1880629	2024/6/5 22:37	2024/6/6 0:34	2024/6/6 10:47	2024/6/6 16:41	2024/6/7 10:05	12.2	18.1	35.5
31	2739955	2024/6/6 18:19	2024/6/6 19:08	2024/6/7 9:24	2024/6/9 9:33	2024/6/9 10:08	15.1	63.2	63.8
32	2736777	2024/6/7 18:57	20244/6/8 8:17	2024/6/8 17:38	2024/6/9 9:11	2024/6/11 10:15	22.7	38.2	87.3
33	2740794	2024/6/10 4:51	2024/6/10 5:56	2024/6/11 9:22	2024/6/11 15:13	2024/6/12 10:39	28.5	34.4	53.8
34	2068125	2024/6/11 3:22	2024/6/11 7:59	2024/6/12 16:07	2024/6/14 9:09	2024/6/14 10:07	36.8	77.8	78.8
35	2742331	2024/6/13 0:14	2024/6/13 1:12	2024/6/13 10:16	2024/6/13 15:01	2024/6/16 8:27	10.0	14.8	80.2
36	2739130	2024/6/14 22:40	2024/6/14 22:54	2024/6/15 11:20	2024/6/16 9:09	2024/6/17 14:47	12.7	34.5	64.1

37	2701751	2024/6/16 17:49	2024/6/16 18:42	2024/6/17 9:08	2024/6/17 14:43	2024/6/18 10:14	15.3	20.9	40.4
38	2678506	2024/6/16 21:43	2024/6/16 23:15	2024/6/17 16:22	2024/6/20 11:11	2024/6/22 9:40	18.6	85.5	132.0
39	2704385	2024/6/17 19:13	2024/6/17 20:19	2024/6/18 15:42	2024/6/20 9:34	2024/6/20 9:37	20.5	62.4	62.4
40	2744294	2024/6/18 1:23	2024/6/18 2:05	2024/6/19 16:10	2024/6/20 9:05	2024/6/23 14:29	38.8	55.7	133.1
41	2744747	2024/6/19 5:36	2024/6/19 9:38	2024/6/23 10:00	2024/6/24 9:43	2024/6/25 14:49	100.4	124.1	153.2
42	1472678	2024/6/19 17:44	2024/6/19 18:31	2024/6/20 9:30	2024/6/20 15:07	2024/6/21 9:09	15.8	21.4	39.4
43	2720521	2024/6/19 21:48	2024/6/19 22:27	2024/6/20 15:59	2024/6/21 9:10	2024/6/22 10:52	18.2	35.4	61.1
44	2744518	2024/6/20 0:42	2024/6/20 1:11	2024/6/21 9:40	2024/6/22 9:02	2024/6/23 11:14	33.0	56.3	82.5
45	2742261	2024/6/20 1:02	2024/6/20 6:31	2024/6/20 15:29	2024/6/21 8:56	2024/6/22 10:53	14.5	31.9	57.9
46	2571720	2024/6/20 20:26	2024/6/20 21:24	2024/6/21 9:23	2024/6/24 11:05	2024/6/24 11:10	13.0	86.7	86.7
47	2068125	2024/6/21 2:29	2024/6/21 2:34	2024/6/22 10:08	2024/6/23 5:52	2024/6/24 10:58	31.7	51.4	80.5
48	839459	2024/6/21 2:01	2024/6/21 2:39	2024/6/21 15:52	2024/6/22 9:03	2024/6/23 11:08	13.9	31.0	57.1
49	2746311	2024/6/22 23:22	2024/6/22 23:55	2024/6/23 17:14	2024/6/24 9:32	2024/6/25 14:08	17.9	34.2	62.8
50	2746324	2024/6/23 0:41	2024/6/23 1:19	2024/6/24 14:24	2024/6/24 14:46	2024/6/25 11:30	37.7	38.1	58.8
51	2746104	2024/6/23 6:50	2024/6/23 4:47	2024/6/23 17:01	2024/6/24 9:31	2024/6/25 14:21	10.2	26.7	55.5
52	2703582	2024/6/23 17:35	2024/6/23 18:01	2024/6/24 11:24	2024/6/24 14:49	2024/6/25 14:24	17.8	21.2	44.8
53	1101486	2024/6/23 18:59	2024/6/23 19:48	2024/6/24 14:41	2024/6/27 11:37	2024/6/27 11:39	19.7	88.6	88.7
54	2086131	2024/6/23 21:12	2024/6/23 22:02	2024/6/24 14:36	2024/6/25 9:05	2024/6/26 15:37	17.4	35.9	66.4
55	1970178	2024/6/23 22:23	2024/6/23 23:22	2024/6/24 11:17	2024/6/24 14:51	2024/6/25 14:24	12.9	16.5	40.0
56	1100968	2024/6/24 20:11	2024/6/24 21:05	2024/6/26 14:52	2024/6/26 14:54	2024/6/27 11:34	42.7	42.7	63.4
57	2747567	2024/6/25 18:51	2024/6/25 19:26	2024/6/26 9:25	2024/6/26 14:47	2024/6/27 11:28	14.6	19.9	40.6
58	1693385	2024/6/26 17:48	2024/6/26 18:24	2024/6/28 8:50	2024/6/28 14:46	2024/6/29 17:46	39.0	45.0	72.0
59	2619593	2024/6/26 17:59	2024/6/26 19:34	2024/6/27 10:21	2024/6/28 9:26	2024/6/30 11:49	16.4	39.5	89.8
60	2747539	2024/6/26 20:49	2024/6/26 19:13	2024/6/27 14:39	2024/6/28 9:07	2024/6/30 11:40	17.8	36.3	86.9
61	2739928	2024/6/26 22:23	2024/6/26 22:59	2024/6/28 14:48	2024/6/28 10:00	2024/6/30 11:09	40.4	35.6	84.8
62	2748115	2024/6/26 22:49	2024/6/27 0:05	2024/6/27 10:23	2024/6/28 9:27	2024/6/29 17:40	11.6	34.6	66.9
63	2583334	2024/6/28 22:00	2024/6/29 7:51	2024/6/30 16:18	2024/7/1 9:01	2024/7/2 10:10	42.3	59.0	84.2
64	2748874	2024/6/29 2:33	2024/6/29 7:53	2024/6/30 9:40	2024/7/1 8:57	2024/7/2 10:14	31.1	54.4	79.7
65	1960381	2024/6/29 19:35	2024/6/30 7:12	2024/6/30 17:24	2024/7/1 8:59	2024/7/2 9:45	21.8	37.4	62.2
66	2748780	2024/6/30 0:07	2024/6/30 7:16	2024/7/1 9:19	2024/7/1 14:34	2024/7/2 10:56	33.2	38.5	58.8
67	2749142	2024/6/29 20:53	2024/6/30 7:18	2024/6/30 16:09	2024/7/1 9:01	2024/7/2 10:06	19.3	36.1	61.2
68	1693385	2024/6/30 19:45	2024/6/30 21:26	2024/7/2 8:43	2024/7/2 14:55	2024/7/3 10:11	37.0	43.2	62.4
69	2751005	2024/7/3 18:20	2024/7/3 18:38	2024/7/4 9:24	2024/7/4 14:30	2024/7/5 14:10	15.1	20.2	43.8
70	2071098	2024/7/7 20:45	2024/7/8 7:57	2024/7/8 15:17	2024/7/9 9:06	2024/7/11 14:25	18.5	36.4	89.7
71	2752958	2024/7/8 18:32	2024/7/8 19:06	2024/7/9 10:39	2024/7/9 14:42	2024/7/10 10:31	16.1	20.2	40.0
72	2753019	2024/7/8 23:08	2024/7/8 23:59	2024/7/9 11:20	2024/7/914:41	2024/7/10 9:52	12.2	NA	34.7
73	2385072	2024/7/10 18:50	2024/7/10 20:26	2024/7/14 9:16	2024/7/15 8:52	2024/7/16 10:27	86.4	110.0	135.6
74	654420	2024/7/11 0:54	2024/7/11 1:42	2024/7/11 16:14	2024/7/12 9:17	2024/7/14 14:41	15.3	32.4	85.8
75	1064137	2024/7/12 20:43	2024/7/12 21:34	2024/7/13 15:39	2024/7/14 8:49	2024/7/15 14:54	18.9	36.1	66.2
76	2755007	2024/7/13 21:09	2024/7/13 22:04	2024/7/15 10:17	2024/7/16 9:26	2024/7/17 14:16	37.1	60.3	89.1
77	2755324	2024/7/14 19:17	2024/7/14 19:41	2024/7/15 9:24	2024/7/15 15:00	2024/7/16 10:28	14.1	19.7	39.2
78	2449336	2024/7/14 19:04	2024/7/14 19:52	2024/7/15 9:36	2024/7/15 14:59	2024/7/16 10:27	14.5	19.9	39.4
79	2757741	2024/7/19 19:43	2024/7/19 21:11	2024/7/20 10:49	2024/7/20 14:24	2024/7/21 10:51	15.1	18.7	39.1
80	2758078	2024/7/21 1:42	2024/7/21 3:16	2024/7/21 9:56	2024/7/21 14:45	2024/7/22 11:45	8.2	13.1	34.1
81	2756282	2024/7/21 19:41	2024/7/21 20:33	2024/7/22 15:03	2024/7/23 8:55	2024/7/24 14:36	19.4	37.2	66.9
82	2378914	2024/7/21 22:37	2024/7/21 23:34	2024/7/22 11:10	2024/7/23 10:37	2024/7/26 8:13	12.6	36.0	105.6
83	2587431	2024/7/24 22:58	2024/7/24 23:56	2024/7/25 15:07	2024/7/26 9:26	2024/7/27 11:18	16.2	34.5	60.3

84	2634722	2024/7/25 18:22	2024/7/25 19:02	2024/7/26 8:53	2024/7/26 14:14	2024/7/27 15:26	14.5	19.9	45.1
85	2760399	2024/7/25 19:53	2024/7/25 20:53	2024/7/26 17:37	2024/7/27 9:11	2024/7/28 11:35	21.7	37.3	63.7
86	2710535	2024/7/27 18:08	2024/7/27 18:57	2024/7/28 9:33	2024/7/28 14:22	2024/7/29 10:45	15.4	20.2	40.6
87	2761135	2024/7/27 23:23	2024/7/27 23:37	2024/7/29 9:15	2024/7/31 10:06	2024/7/31 14:22	33.9	82.7	87.0
88	2761975	2024/7/29 18:15	2024/7/29 20:01	2024/7/30 9:29	2024/7/31 9:10	2024/8/1 14:40	15.2	38.9	68.4
89	2760431	2024/7/31 19:21	2024/7/31 19:46	2024/8/1 16:24	2024/8/2 9:00	2024/8/3 10:06	21.0	37.6	62.7

Table 6 Laboratory endpoints of Phase III Post-op

ID	Admission number	Blood culture collection	Blood culture loading time	Gram staining report	Species identification	Preliminary AST report time	Final AST report time	TAT for Gram-staining reports of positive cultures	Time to microbial species identification for positive cultures	Total TAT from specimen collection to clinician receipt of reports
1	2759818	2024/7/31 23:45	2024/8/1 0:33	2024/8/1 16:22	2024/8/2 9:15	2024/8/3 9:20	2024/8/3 10:33	16.6	33.5	57.6
2	2763190	2024/8/1 12:37	2024/8/1 14:44	2024/8/3 11:02	2024/8/3 15:00	2024/8/4 2:46	2024/8/4 10:33	46.4	50.4	62.2
3	2761991	2024/8/6 11:07	2024/8/6 11:30	2024/8/7 16:22	2024/8/8 15:36	2024/8/9 8:48	2024/8/9 15:23	29.3	52.5	69.7
4	1332798	2024/8/9 17:42	2024/8/9 18:56	2024/8/10 17:43	2024/8/11 9:22	2024/8/11 21:08	2024/8/12 14:23	24.0	39.7	51.4
5	2766222	2024/8/11 3:47	2024/8/11 5:08	2024/8/11 16:30	2024/8/12 9:01	2024/8/12 20:02	2024/8/13 15:02	12.7	29.2	40.3
6	2763399	2024/8/14 18:40	2024/8/14 20:28	2024/8/15 8:23	2024/8/15 14:26	2024/8/15 22:50	2024/8/16 10:51	13.7	19.8	28.2
7	2771837	2024/8/22 15:18	2024/8/22 15:52	2024/8/24 7:46	2024/8/24 9:01	2024/8/24 22:58	2024/8/25 10:35	40.5	41.7	55.7
8	2777258	2024/9/8 16:01	2024/9/8 16:59	2024/9/9 16:43	2024/9/10 8:57	2024/9/10 19:44	2024/9/11 15:09	24.7	40.9	51.7
9	2758590	2024/9/14 5:44	2024/9/14 9:12	2024/9/15 9:08	2024/9/15 14:30	2024/9/16 2:46	2024/9/16 15:27	27.4	32.8	45.0
10	2763220	2024/9/15 10:52	2024/9/15 11:38	2024/9/16 10:21	2024/9/16 13:50	2024/9/16 21:24	2024/9/17 7:31	23.5	27.0	34.5
11	2778164	2024/9/16 19:22	2024/9/16 19:48	2024/9/17 9:51	2024/9/17 15:09	2024/9/18 15:46	2024/9/18 16:26	14.5	19.8	44.4
12	2781975	2024/9/19 20:02	2024/9/19 21:14	2024/9/20 8:53	2024/9/20 14:26	2024/9/20 22:18	2024/9/21 14:22	12.8	18.4	26.3
13	2285142	2024/9/22 15:21	2024/9/22 16:04	2024/9/23 11:18	2024/9/23 15:00	2024/9/24 4:24	2024/9/24 10:35	20.0	23.7	37.1
14	2784306	2024/9/26 17:55	2024/9/26 18:17	2024/9/28 10:56	2024/9/28 15:05	2024/9/29 4:58	2024/9/29 9:56	41.0	45.2	59.0
15	2264591	2024/8/26 22:04	2024/8/26 22:19	2024/8/28 12:00	2024/8/29 9:01	2024/8/30 0:24	2024/8/30 9:58	37.9	59.0	74.3
16	2778701	2024/9/12 16:08	2024/9/12 16:57	2024/9/13 15:50	2024/9/14 10:13	2024/9/14 19:10	2024/9/15 10:13	23.7	42.1	51.0
17	2766968	2024/8/10 13:40	2024/8/10 15:58	2024/8/11 15:23	2024/8/12 8:49	2024/8/12 19:48	2024/8/13 14:32	25.7	43.1	54.1
18	2767941	2024/8/13 13:38	2024/8/13 15:40	2024/8/14 16:28	2024/8/15 8:58	2024/8/16 0:02	2024/8/16 10:34	26.8	43.3	58.4
19	1355432	2024/8/3 4:10	2024/8/3 9:21	2024/8/4 9:12	2024/8/4 14:48	2024/8/5 8:22	2024/8/5 14:13	29.0	34.6	52.2
20	611745	2024/8/3	2024/8/3	2024/8/5	2024/8/6	2024/8/7 9:46	2024/8/7	49.8	67.8	91.0

		14:45	15:57	16:34	10:36		14:19			
21	2760181	2024/8/1 6:33	2024/8/1 8:16	2024/8/5 16:26	2024/8/6 9:47	2024/8/7 10:48	2024/8/8 15:25	NA	NA	NA
22	777143	2024/8/13 15:14	2024/8/13 15:40	2024/8/14 10:56	2024/8/14 14:52	2024/8/15 9:04	2024/8/15 14:58	19.7	23.6	41.8
23	2703425	2024/8/12 12:24	2024/8/12 15:05	2024/8/13 9:50	2024/8/14 8:24	2024/8/14 18:02	2024/8/15 14:37	21.4	44.0	53.6
24	704880	2024/8/27 10:49	2024/8/27 11:34	2024/8/28 14:49	2024/8/29 9:15	2024/8/30 5:06	2024/8/30 14:45	28.0	46.4	66.3
25	2523736	2024/10/8 11:37	2024/10/8 15:06	2024/10/9 9:11	2024/10/9 13:50	2024/10/10 1:46	2024/10/10 11:12	21.6	26.2	38.2
26	2769623	2024/8/16 20:22	2024/8/17 9:08	2024/8/18 9:24	2024/8/19 9:10	2024/8/20 15:04	2024/8/21 16:05	37.0	60.8	90.7
27	2782322	2024/9/26 10:16	2024/9/26 11:03	2024/9/27 11:08	2024/9/27 14:39	2024/9/28 8:24	2024/9/28 11:17	24.9	28.4	46.1
28	1392545	2024/8/14 10:33	2024/8/14 11:05	2024/8/16 8:44	2024/8/16 15:11	2024/8/17 0:32	2024/8/17 10:46	46.2	52.6	62.0
29	1090243	2024/9/16 9:45	2024/9/16 10:59	2024/9/17 8:59	2024/9/17 15:08	2024/9/18 1:02	2024/9/18 15:46	23.2	29.4	39.3
30	2619921	2024/10/11 15:48	2024/10/11 16:37	2024/10/12 16:36	2024/10/13 9:11	2024/10/14 10:12	2024/10/15 11:28	24.8	41.4	66.4
31	2648439	2024/8/28 14:44	2024/8/28 15:56	2024/8/29 10:12	2024/8/29 15:02	2024/8/30 2:00	2024/8/30 10:16	19.5	24.3	35.3
32	2764553	2024/8/6 19:14	2024/8/6 19:52	2024/8/7 15:58	2024/8/8 8:48	2024/8/8 19:16	2024/8/9 15:29	20.7	37.6	48.0
33	778829	2024/10/5 16:28	2024/10/5 16:47	2024/10/6 9:51	2024/10/6 14:11	2024/10/7 8:12	2024/10/8 14:18	17.4	21.7	39.7
34	2534097	2024/8/9 9:37	2024/8/9 11:08	2024/8/10 8:49	2024/8/10 14:52	2024/8/11 0:22	2024/8/11 11:07	23.2	29.3	38.8
35	2781017	2024/9/17 14:47	2024/9/17 16:00	2024/9/19 9:20	2024/9/19 14:48	2024/9/20 0:52	2024/9/20 14:26	42.6	48.0	58.1
36	2775571	2024/9/1 15:46	2024/9/1 16:46	2024/9/2 16:42	2024/9/3 8:56	2024/9/4 9:14	2024/9/4 14:47	24.9	41.2	65.5
37	2786752	2024/10/4 19:24	2024/10/4 21:19	2024/10/5 8:57	2024/10/5 14:13	2024/10/6 8:39	2024/10/6 10:32	13.5	18.8	37.3
38	2528602	2024/10/8 10:12	2024/10/8 11:10	2024/10/9 8:52	2024/10/9 13:51	2024/10/9 22:32	2024/10/10 14:18	22.7	27.6	36.3
39	2526435	2024/10/6 14:32	2024/10/6 15:36	2024/10/7 15:52	2024/10/8 9:10	2024/10/8 23:08	2024/10/9 15:40	25.3	42.6	56.6
40	2327002	2024/9/29 20:13	2024/9/29 20:36	2024/9/30 10:17	2024/9/30 14:37	2024/9/30 23:34	2024/10/1 11:30	14.1	18.4	27.3
41	2774980	2024/8/31 20:58	2024/8/31 22:33	2024/9/1 14:31	2024/9/1 14:50	2024/9/2 1:46	2024/9/2 10:50	17.5	17.9	28.8
42	2763922	2024/8/17 16:17	2024/8/17 16:47	2024/8/18 11:31	2024/8/18 14:15	2024/8/19 2:40	2024/8/19 14:24	19.2	22.0	34.4
43	2767920	2024/8/12 16:43	2024/8/12 17:09	2024/8/13 9:59	2024/8/14 10:01	2024/8/15 9:00	2024/8/15 14:24	17.3	41.3	64.3
44	2769461	2024/8/16	2024/8/16	2024/8/17	2024/8/17	2024/8/18	2024/8/18	27.5	28.0	46.3

		10:44	15:54	14:12	14:44	9:01	11:19			
45	1046170	2024/9/5 17:14	2024/9/5 18:41	2024/9/6 8:49	2024/9/6 14:56	2024/9/7 2:58	2024/9/7 15:34	15.6	21.7	33.7
46	2166825	2024/8/14 12:47	2024/8/14 14:26	2024/8/15 14:37	2024/8/16 8:46	2024/8/17 8:40	2024/8/17 1:57	25.8	44.0	67.9
47	2726904	2024/8/4 10:07	2024/8/4 11:11	2024/8/5 9:47	2024/8/5 15:31	2024/8/6 0:10	2024/8/6 15:41	23.7	29.4	38.1
48	2767854	2024/8/26 2:53	2024/8/26 5:05	2024/8/26 16:56	2024/8/27 9:01	2024/8/28 8:19	2024/8/28 11:12	14.1	30.1	53.4
49	2778178	2024/10/8 15:11	2024/10/8 15:50	2024/10/9 16:44	2024/10/10 9:49	2024/10/10 22:20	2024/10/12 13:42	25.5	42.6	55.1
50	2772022	2024/9/7 9:54	2024/9/7 10:59	2024/9/8 14:29	2024/9/8 15:06	2024/9/9 9:08	2024/9/9 14:09	28.6	29.2	47.2
51	730119	2024/9/16 10:05	2024/9/16 11:00	2024/9/17 8:51	2024/9/17 15:21	2024/9/17 23:20	2024/9/19 9:15	22.8	29.3	37.2
52	857117	2024/8/12 10:02	2024/8/12 11:14	2024/8/13 9:12	2024/8/13 16:25	2024/8/14 10:02	2024/8/14 14:11	23.2	30.4	48.0
53	1387457	2024/10/8 10:00	2024/10/8 11:11	2024/10/9 9:20	2024/10/9 13:53	2024/10/10 3:40	2024/10/10 14:13	23.3	27.9	41.7
54	2765528	2024/8/7 9:11	2024/8/7 11:05	2024/8/8 9:16	2024/8/10 9:15	2024/8/10 10:54	2024/8/11 11:07	24.1	72.1	73.7
55	2701751	2024/9/12 14:49	2024/9/12 15:57	2024/9/14 10:39	2024/9/14 14:42	2024/9/14 23:32	2024/9/15 10:37	43.8	47.9	56.7
56	590168	2024/9/12 17:04	2024/9/12 17:22	2024/9/14 11:00	2024/9/14 14:44	2024/9/15 0:16	2024/9/15 10:50	41.9	45.7	55.2
57	2783509	2024/9/24 15:44	2024/9/24 16:45	2024/9/25 9:23	2024/9/25 14:33	2024/9/26 0:14	2024/9/26 9:59	17.7	22.8	32.5
58	2786629	2024/10/6 15:04	2024/10/6 15:25	2024/10/7 8:48	2024/10/7 14:02	2024/10/7 23:36	2024/10/8 13:38	17.7	23.0	32.5
59	2760611	2024/8/5 12:31	2024/8/5 16:02	2024/8/8 10:59	2024/8/8 14:43	2024/8/9 8:46	2024/8/9 14:43	70.5	74.2	92.3
60	2764260	2024/8/4 10:10	2024/8/4 11:13	2024/8/5 16:27	2024/8/7 9:31	2024/8/7 20:16	2024/8/8 11:05	30.3	71.3	82.1
61	906072	2024/9/21 13:30	2024/9/21 16:07	2024/9/22 9:58	2024/9/22 14:56	2024/9/23 0:30	2024/9/23 10:43	20.5	25.4	35.0
62	2167849	2024/8/25 14:49	2024/8/25 15:52	2024/8/26 9:31	2024/8/27 8:53	2024/8/27 21:20	2024/8/28 11:08	18.7	42.1	54.5
63	2767039	2024/9/17 0:04	2024/9/17 0:53	2024/9/17 11:28	2024/9/17 15:08	2024/9/18 0:32	2024/9/18 15:32	11.4	15.1	24.5
64	2376632	2024/10/6 17:56	2024/10/6 18:26	2024/10/7 8:44	2024/10/7 14:08	2024/10/8 9:06	2024/10/8 13:44	14.8	20.2	39.2
65	2752918	2024/8/1 15:54	2024/8/1 17:06	2024/8/2 16:59	2024/8/3 10:04	2024/8/3 20:16	2024/8/4 10:19	25.1	42.2	52.4
66	864272	2024/8/2 12:26	2024/8/2 14:25	2024/8/4 9:13	2024/8/5 8:57	2024/8/6 8:12	2024/8/7 11:24	44.8	68.5	91.8
67	2761707	2024/8/2 9:58	2024/8/2 11:10	2024/8/4 15:09	2024/8/5 8:43	2024/8/5 19:16	2024/8/6 11:24	53.2	70.7	81.3
68	2783778	2024/9/29	2024/9/29	2024/9/30	2024/9/30	2024/10/1	2024/10/1	19.2	23.4	34.5

		15:13	16:00	10:27	14:37	1:46	11:29			
69	2764085	2024/8/3 19:48	2024/8/3 20:58	2024/8/4 15:13	2024/8/5 8:59	2024/8/6 10:01	2024/8/7 11:25	19.4	37.2	62.2
70	2765827	2024/8/7 16:06	2024/8/7 16:55	2024/8/9 11:46	2024/8/9 15:06	2024/8/10 0:30	2024/8/10 11:07	43.7	47.0	56.4
71	1493671	2024/8/31 16:26	2024/8/31 16:55	2024/9/1 9:07	2024/9/1 14:46	2024/9/1 23:24	2024/9/2 11:21	16.7	22.3	31.0
72	2736203	2024/9/9 17:33	2024/9/9 17:44	2024/9/10 11:00	2024/9/10 15:18	2024/9/11 1:46	2024/9/11 15:17	17.5	21.8	32.2
73	2088734	2024/10/11 12:36	2024/10/11 15:00	2024/10/12 9:05	2024/10/12 14:12	2024/10/13 9:28	2024/10/13 14:21	20.5	25.6	44.9
74	1268042	2024/9/4 8:11	2024/9/4 9:37	2024/9/5 9:29	2024/9/5 14:28	2024/9/8 9:32	2024/9/8 10:43	25.3	30.3	97.4
75	2606574	2024/9/15 10:02	2024/9/15 10:50	2024/9/16 10:41	2024/9/16 13:52	2024/9/17 0:56	2024/9/17 10:17	24.7	27.8	38.9
76	1862461	2024/8/19 14:56	2024/8/19 15:58	2024/8/20 8:29	2024/8/20 15:23	2024/8/21 9:16	2024/8/22 11:35	17.6	24.5	42.3
77	2713090	2024/8/11 15:57	2024/8/11 16:44	2024/8/12 9:39	2024/8/12 14:46	2024/8/12 21:52	2024/8/13 15:08	17.7	22.8	29.9
78	2738031	2024/9/16 16:40	2024/9/16 17:21	2024/9/17 8:46	2024/9/17 15:08	2024/9/17 23:22	2024/9/18 15:48	16.1	22.5	30.7
79	1665049	2024/8/19 10:25	2024/8/19 11:15	2024/8/22 10:08	2024/8/22 15:45	2024/8/23 8:26	2024/8/26 10:05	71.7	77.3	94.0
80	1148238	2024/10/10 14:08	2024/10/10 15:58	2024/10/11 15:40	2024/10/12 8:14	2024/10/12 17:04	2024/10/13 13:54	25.5	42.1	50.9
81	2488372	2024/9/19 10:29	2024/9/19 15:53	2024/9/20 10:38	2024/9/20 14:29	2024/9/21 2:12	2024/9/21 14:22	24.2	28.0	39.7
82	2785350	2024/9/30 13:07	2024/9/30 16:04	2024/10/1 10:35	2024/10/1 15:04	2024/10/2 8:48	2024/10/2 10:43	21.5	26.0	43.7
83	563229	2024/9/13 17:25	2024/9/13 18:11	2024/9/16 7:25	2024/9/17 9:13	2024/9/17 20:48	2024/9/18 15:49	62.0	87.8	99.4
84	2017592	2024/8/15 13:35	2024/8/15 14:10	2024/8/17 10:34	2024/8/17 14:12	2024/8/18 9:01	2024/8/18 10:54	45.0	48.6	67.4
85	2785890	2024/10/1 10:34	2024/10/1 11:37	2024/10/2 9:19	2024/10/2 14:49	2024/10/2 22:58	2024/10/3 10:50	22.7	28.2	36.4
86	2786019	2024/10/11 9:28	2024/10/11 11:12	2024/10/12 8:59	2024/10/12 14:10	2024/10/12 22:42	2024/10/13 14:20	23.5	28.7	37.2
87	2782142	2024/9/20 11:18	2024/9/20 11:59	2024/9/21 9:17	2024/9/21 14:32	2024/9/21 22:48	2024/9/22 15:42	22.0	27.2	35.5
88	2598710	2024/9/11 18:04	2024/9/11 19:01	2024/9/12 9:59	2024/9/12 14:55	2024/9/13 9:30	2024/9/13 17:03	15.9	20.9	39.4
89	2764105	2024/8/3 20:58	2024/8/3 21:54	2024/8/4 9:06	2024/8/4 14:48	2024/8/5 8:20	2024/8/5 14:11	12.1	17.8	35.4

NA = not applicable
